# Exposure to false cardiac feedback alters pain perception and anticipatory cardiac frequency

**DOI:** 10.7554/eLife.90013

**Published:** 2026-06-15

**Authors:** Eleonora Parrotta, Patric Bach, Giovanni Pezzulo, Andrea Zaccaro, Mauro Gianni Perrucci, Marcello Costantini, Francesca Ferri

**Affiliations:** 1 Department of Psychology, Sapienza University of Rome Rome Italy; 2 School of Psychology, University of Aberdeen Aberdeen United Kingdom; 3 Department of Neuroscience, Imaging and Clinical Sciences, “G. d'Annunzio” University of Chieti-Pescara Chieti Italy; 4 Institute of Cognitive Sciences and Technologies, National Research Council Rome Italy; 5 Institute for Advanced Biomedical Technologies ‑ ITAB, “G. d'Annunzio” University of Chieti-Pescara Chieti Italy; 6 UdA-TechLab, Research Center, University “G. d’Annunzio” of Chieti-Pescara Chieti Italy; 7 Department of Psychology, “G. d’Annunzio” University of Chieti-Pescara Chieti Italy; National Scientific and Technical Research Council (CONICET), National University of Entre Ríos (UNER) Argentina; University Medical Center Hamburg-Eppendorf Germany

**Keywords:** predictive processing, interoceptive illusion, pain perception, bayesian inference, cardiac feedback, Human

## Abstract

The experience of pain, like other interoceptive processes, has recently been conceptualized in terms of predictive coding and free energy frameworks. In these views, the brain integrates sensory, proprioceptive, and interoceptive signals to generate probabilistic inferences about upcoming events, which shape both the state and the perception of our inner body. Here, we ask whether it is possible to induce pain expectations by providing false faster (vs. slower) acoustic cardiac feedback before administering electrical cutaneous shocks. We test whether these expectations will shape both the perception of pain and the body’s physiological state toward prior predictions. Results confirmed that faster cardiac feedback elicited pain expectations that affected both perceptual pain judgments and the body’s physiological response. Perceptual pain judgments were biased toward the expected level of pain, such that participants illusorily perceived identical noxious stimuli as more intense and unpleasant. Physiological changes mirrored the predicted level of pain, such that participants’ actual cardiac response in anticipation of pain stimuli showed a deceleration in heart rate, in line with the well-known orienting cardiac response in anticipation of threatening stimuli (Experiment 1). In a control experiment, such perceptual and cardiac modulations were dramatically reduced when the feedback reproduced an exteroceptive, instead of interoceptive, cardiac feedback (Experiment 2). These findings show that cardiac perception can be understood as interoceptive inference that modulates both our perception and the physiological state of the body, thereby actively generating the interoceptive and autonomic consequences that have been predicted.

## Introduction

Far more complex than the mere transmission of nociceptive inputs, pain is a component of the interoceptive system (Craig, 2003b). It is defined as an unpleasant sensory and emotional experience associated with, or resembling, actual or potential tissue damage (International Association for the Study of Pain, IASP, 2020). It can occur in the absence of physical harm (Loeser and Treede, 2008) and is permeated by all the available sources of information, including sensory, emotional, cognitive, and social components, as well as prior information and expectations (Atlas and Wager, 2012; Tracey, 2010; de C Williams and Craig, 2016). While the last decades provided important advances in the understanding of pain, developing an overarching theory that accounts for all the dimensions of pain still remains challenging (for a full discussion, see Moayedi and Davis, 2013).

Current views propose predictive processing models as a suitable candidate to account for the multifaceted experience of pain (Büchel et al., 2014; Kiverstein et al., 2022; Song et al., 2019; Song et al., 2021). Interoceptive inference and the Embodied Predictive Coding frameworks (e.g. Barrett, 2017; Barrett and Simmons, 2015; Pezzulo, 2014; Seth, 2013; Seth and Friston, 2016) conceptualize the brain as an active generator of inferences, which maintains an internal model of both the internal and the external worlds and attempts to fit it with incoming inputs through a process of Bayesian hypothesis testing and revision. Interoceptive sensations are thought to be derived from the integration of different sources of information into an expectation of the body’s upcoming changes, which are kept in check by the actual state of the body (Barrett and Simmons, 2015). However, people’s predictions do not always mirror reality, but can be inaccurate, leading to a gap between the expected and the actual present state. The goal of inferential processes is to reduce this difference between one’s internal models and the actual interoceptive input, thus converging toward the *brain’s best guess* about the body’s state (Ainley et al., 2016; Seth et al., 2011; Seth and Friston, 2016).

An important feature of these accounts is that the difference between one’s internal models and the sensory data can be minimized not only by updating internal models to better fit the data (i.e. perceptual inference) but also by performing actions, changing bodily states themselves (i.e. active inference, see Friston, 2010; Friston, 2005; Parr et al., 2022). For example, an expectation of a threatening, painful stimulus could be realized both by making the painful stimulus *appear* more painful than it really is, thereby fitting one’s internal model to bodily reality, or by *actually* modulating the state of the body and shaping the physiologic response to pain towards previous predictions, for example by engaging autonomic reflexes (e.g. typical decrease in the heart rate in relation to pain anticipation, e.g. Bradley et al., 2008, Bradley et al., 2005; Colloca et al., 2006; Lykken et al., 1972; Taggart et al., 1976; Tracy et al., 2017).

There is suggestive evidence for both components in the extant literature on pain. The assumption that prior information modulates pain perception underlines decades of work on placebo and nocebo effects (i.e. the expectation-effect; Crombez et al., 1998; Keltner et al., 2006; Petrovic and Ingvar, 2002; Price et al., 1999; Wager, 2005; Wager et al., 2004). On a neuronal level, expecting pain alters the neural mechanisms in pain-processing regions, thought to reflect a combination of nociceptive inputs and top-down information (Atlas et al., 2010; Keltner et al., 2006; Koyama et al., 2005; Wiech et al., 2008), even at a very early stage of processing (Eippert et al., 2009; Geuter and Büchel, 2013). Moreover, such changes can persist - or even grow over time - in the absence of disconfirming evidence (Atlas et al., 2010; Colloca et al., 2010; Craggs et al., 2008; Jepma and Wager, 2015; Koban and Wager, 2016; Montgomery and Kirsch, 1997; Vase et al., 2005; Vase et al., 2011), turning pain experience into potentially a self-fulfilling prophecy (Jepma et al., 2018).

As for the assumption that unfulfilled predictions can be resolved by engaging autonomic reactions, there is evidence that prior pain expectations, and pain itself, modulate physiological responses. Expecting pain induces augmented activity in the sympathetic system (e.g. increases in blood pressure and skin conductance) to prepare potential avoidance responses (Barlow et al., 1996; Oka et al., 2007; Tousignant‐Laflamme and Marchand, 2006; Yang et al., 2003), as well as a characteristic decrease in heart rate to promote orienting responses, attention, and sensory processing (Bradley et al., 2008; Bradley et al., 2005; Colloca et al., 2006; Lykken et al., 1972; Taggart et al., 1976; Tracy et al., 2017; for a full discussion, see Skora et al., 2022). Once pain is actually experienced, however, heart rate rises, reflecting robust sympathetic activation and a well-documented autonomic response to nociceptive input (Tousignant-Laflamme et al., 2005; Terkelsen et al., 2005; for a review, see Forte et al., 2022).

Together, these findings support the idea that the experience of pain may rely on top-down predictive mechanisms, which shape not only the subjective perception of pain but also its neural and bodily correlates, both when pain is experienced and merely expected. However, to our knowledge, no study exists that tracks both the perceptual and physiological responses to mismatches between pain expectation and bodily reality. Moreover, in previous work, pain expectations were typically induced by exteroceptive cues (e.g. visual, Wiech et al., 2014; Jepma et al., 2018; auditory, Colloca et al., 2006; Atlas et al., 2010). Although interoceptive processes may have contributed to the observed effects, these studies did not specifically target interoceptive sources of information within the inferential process.

If one assumes that pain and bodily states are integrated within a unified interoceptive system, then information about the internal condition of the body should be tightly linked to the experience of pain. Among such interoceptive streams, cardiac activity appears to be closely intertwined with the experience of pain, likely due to a close integration between the pain system and the neural circuits involved in cardiovascular regulation (Craig, 2003b; Craig, 2003a; Craig, 2008). For example, pain-related evoked potentials and nociception are modulated across the cardiac cycle (Edwards et al., 2001; Edwards et al., 2002; Edwards et al., 2008; Martins et al., 2009; McIntyre et al., 2006), and both the anticipation of and the response to pain are associated with changes in heart rates (Colloca et al., 2006; Mischkowski et al., 2018; for a review, see Kyle and McNeil, 2014). Moreover, when estimating their own cardiac frequency, people’s estimates integrate the level of threat (i.e. pain) associated with upcoming events (Parrotta et al., 2024), reflecting the (fictitious) belief that anticipating threat increases heart rates. Even though anticipating pain typically decreases real heart rates (the well-known orienting response, Sokolov et al., 2002), people therefore illusorily perceive a higher cardiac frequency when expecting painful stimulation (Parrotta et al., 2024).

The tight coupling between heart rate and pain experience suggests that it should be possible to modulate the experience of pain – and the anticipatory bodily response – by manipulating *interoceptive cardiac* feedback, that is, by misleading people into believing that their heart rate is increasing in anticipation of a noxious stimulus. Under predictive architectures, persuading one’s internal model towards the fictitious evidence of increased heart rates should then induce changes both in *pain perception* (i.e. perceptual inference) and in the *actual state of the body* (i.e. active inference). Specifically, the former process would correspond to an interoceptive illusion of increased pain, whereas the latter would correspond to physiological and autonomic adaptations to the predicted level of pain. We hypothesized that a cardiac feedback manipulation that renders interoceptive streams more similar to those expected during pain would produce an interoceptive illusion of pain as well as ensuing autonomic adjustments – as hypothesized by interoceptive inference and Embodied Predictive Coding theories. To test this idea, Experiment 1 provided participants with auditory cardiac feedback, via headphones, that could diverge from their actual heart rate by being either faster (as when experiencing pain or threat) or slower (as in relaxed states), before the administration of a noxious electrical stimulation, while their ECG was recorded. Once the pain stimulus was administered, subjects were asked to rate its intensity and unpleasantness.

We predicted, first, that the exposure to faster (vs. slower) cardiac feedback would induce expectations of increased levels of pain, such that noxious stimuli are felt and reported as more unpleasant and intense. Accelerated heart rate is a salient, evolutionarily conserved signal of arousal, threat, and nociception, a mapping continually reinforced physiologically when responding to *actual* pain (e.g. for a review, see Forte et al., 2022). If these expectations are integrated with interoceptive inference, they should therefore heighten assessment of pain.

Second, expectations of threat induced by the faster feedback should be fulfilled through anticipatory parasympathetic reflexes, such that participants’ real heart rates decrease when exposed to the faster, compared to the slower cardiac feedback, in line with the well-known orienting cardiac response when expecting threat or pain (Bradley et al., 2008; Bradley et al., 2005; Colloca et al., 2006; Lykken et al., 1972; Taggart et al., 1976; Tracy et al., 2017; for a full discussion, see Skora et al., 2022). This anticipatory cardiac deceleration reflects an allostatic adjustment implemented by the brain in response to predicted (instead of experienced) threat.

Our hypotheses were theoretically derived from Embodied Predictive Coding accounts of interoception. Our previous findings (Parrotta et al., 2024) suggest that when the brain estimates cardiac activity, it may assign special relevance to pain and anticipated threat, implying that cardiac signals are weighted within the interoceptive schema of pain (Schoeller et al., 2024). In particular, we previously demonstrated that the mere expectation of pain can induce a cardiac interoceptive illusion: participants reported increased heart rate when anticipating pain, even in the absence of any actual physiological change (Parrotta et al., 2024). Here, we tested the counterpart of this illusion: if pain expectations can bias cardiac perception, then manipulating cardiac feedback should, in turn, influence pain perception. Presenting participants with artificially accelerated heartbeat feedback may act as a misleading prior, enhancing perceived pain intensity despite unchanged nociceptive input. Evidence for such a mechanism is further suggested by the evidence that providing mismatched cardiac feedback in contexts where a faster heart rate is normally expected (i.e. sexual arousal, Rupp and Wallen, 2008; Valins, 1966; physical exercise, Iodice et al., 2019) can enhance the perception of physiological states associated with those experiences (Crucian et al., 2000; Iodice et al., 2019).

To rule out that potential modulations in participants’ pain perception and cardiac state were merely associated with the frequency of the feedback, rather than a heartbeat, Experiment 2 replicated the design in a second group of participants, with the only difference being that the congruent or incongruent feedback would not be a heartbeat tone, but an unrelated exteroceptive stimulus. We predicted that, as participants would have no expectation that such an external stimulus signals a preparation for a threatening, painful stimulus, variations in the rate of this exteroceptive stimulus should lead to no (or reduced) perceptual and cardiac changes.

If confirmed, the results would provide the first evidence that predictive processes are generated by the simulation of interoceptive streams, influencing not only our perception of pain but also its heart-related physiological response: two changes that jointly minimize the discrepancy between the expected and the actual homeostatic (interoceptive) state. This evidence would corroborate the assumption that predictions originate along the embodied multisensory sphere by integrating multiple sources of information (i.e. interoceptive) within the inference. Ultimately, the results may provide important insights into how the experience of pain is actively built through predictive processes.

## Results

### Experiment 1

**Figure 1.**
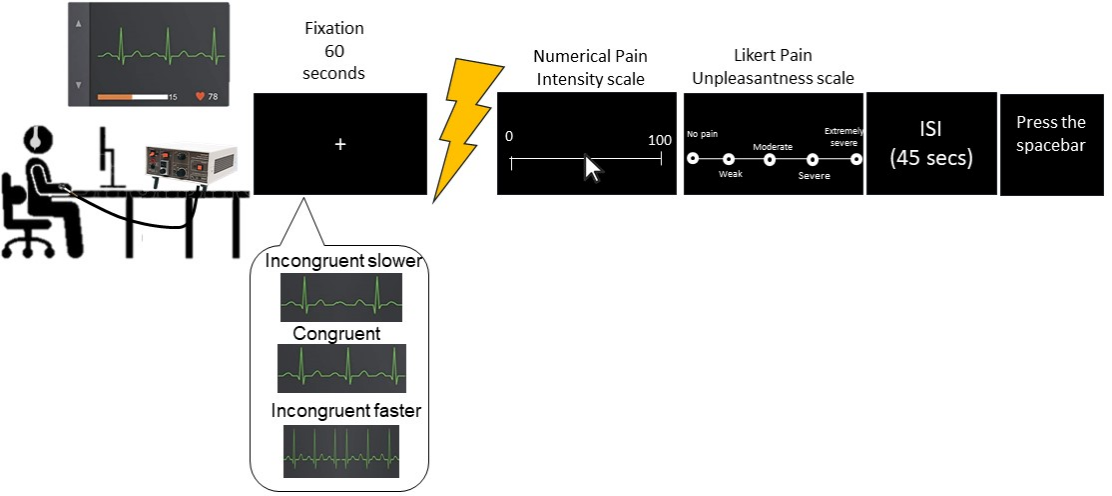
Trial timeline of the experiment. Participants are seated with the electrodes of both the electrical stimulation and the ECG attached. Each trial starts with a fixation cross (60 s), after which the noxious stimulation is administered and the pain intensity (i.e. Numerical Rating Scale) and unpleasantness (i.e. Likert scale) ratings are collected. After each trial, a pause screen of 45 s was shown (i.e. ISI, Inter Stimulus Interval), after which participants proceeded to the next trial by pressing the spacebar. The trial timeline was identical for both the no-feedback and feedback phases, with the only exception that in the feedback phase participants were provided with the acoustic feedback (i.e. slower, faster, congruent) about their HR, reproduced for the whole fixation period (60 s) preceding the shock.

Experiment 1 tests whether false cardiac feedback of an accelerated (i.e. faster), relative to a decelerated (i.e. slower) heart rate, alters both pain perception and the cardiac anticipatory response to noxious stimuli (Figure 1).

#### ECG heart rate measurement

**Figure 2.**
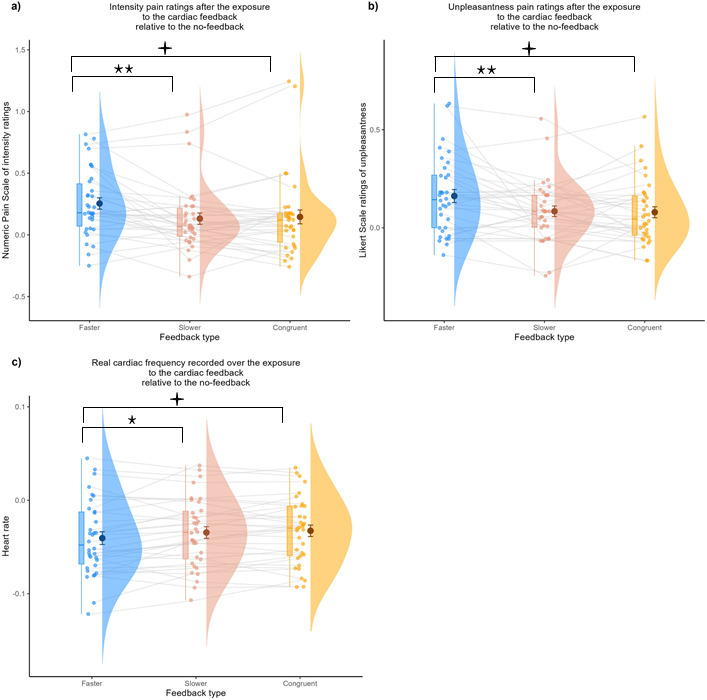
Interoceptive cardiac feedback shapes subjective pain ratings and anticipatory heart-rate responses. (**a**) Intensity pain ratings (Numeric Pain Scale), (**b**) Unpleasantness ratings (5-points Likert Scale), (**c**) Real cardiac frequency. Values consist of the central tendency of change of pain intensity (**a**), unpleasantness (**b**), and heart rate (**c**) associated with the exposure to the interoceptive faster, slower, and congruent cardiac feedback, relative to the no-feedback. Values of zero on the vertical axis would represent no change relative to the exposure to the no-feedback. Positive and negative values would represent an increase and a decrease, respectively. Brackets denote significance: = significant one-way RM-ANOVA; short bracket = planned Faster–Slower contrast. Asterisks indicate two-tailed *p* values (*<0.05, **<0.01, ***<0.001, “n.s.”=not significant).

We first tested whether the heart rate feedback was treated as an interoceptive stimulus and induced a compensatory adjustment of the real heart rate. If so, faster feedback should elicit a comparative slowdown of real heart rates compared to slower feedback (see Figure 2c). An initial ANOVA confirmed that the different types of heart rate feedback (faster, congruent, slower) affected heart rates, *F*(2, 66) = 4.071, p=0.022, *ηp²*=0.110. A planned paired sample t-test showed that heart rates decreased during faster cardiac feedback compared to slower heart rate feedback, *t*(33) = 2.074, p=0.046, *d*=0.36 (Figure 2c). When compared with congruent feedback, significant heart rate differences emerged for faster feedback, *t*(33) = 2.228, p=0.033, *d*=0.38, but not for slower feedback, *t*(33) = 0.92, p=0.364, *d*=0.16.

#### Subjective pain intensity ratings

Participants’ pain intensity ratings were analyzed analogously. If faster heart rates are treated as an interoceptive signal of threat, then faster cardiac feedback should lead to increased pain intensity ratings compared to slower feedback. A repeated measures ANOVA with three levels (faster feedback, congruent feedback, slower feedback) revealed that the types of heart rate feedback affected pain intensity ratings, *F*(2, 66) = 7.017, p=0.002, *ηp²*=0.175. A planned t-test then revealed that participants’ perception of pain intensity increased after faster relative to slower cardiac feedback, *t*(33) = 3.339, p=0.002, *d*=0.573 (Figure 2a). Additional t-tests showed that, like in the heart rate recordings, this difference was largely driven by the faster feedback. When compared to congruent feedback, significant differences emerged for faster feedback, *t*(33) = 2.66, p=0.012, *d*=0.457, but not for the slower feedback, which showed a slight numerical decrease, *t*(33) = 0.477, p=0.636, *d*=0.082.

#### Subjective pain unpleasantness ratings

Analogous tests run on pain unpleasantness ratings replicated all findings. The initial ANOVA confirmed that the different types of cardiac feedback (faster, congruent, slower) affected pain unpleasantness ratings, F(2, 66) = 6.092, p=0.004, *ηp²*=0.156. A paired sample t-test showed that pain unpleasantness ratings were higher after faster feedback than slower cardiac feedback, *t*(33) = 2.771, p=0.009, *d*=0.48 (Figure 2b). When compared to congruent feedback, significant differences emerged for faster feedback, *t*(33) = 3.045, p=0.005, *d*=0.522, but not for the slower feedback, which showed a slight numerical decrease, *t*(33) = 0.206, p=0.838, *d*=0.035.

#### Body Perception Questionnaire

The Short form Body Perception Questionnaire (Poli et al., 2021), administered at the end of the experiment, allowed us to explore whether individual differences in body awareness and supradiaphragmatic reactivity were related to the extent to which participants developed (1) the perceptual illusion of pain (2) the physiological changes in their cardiac frequency. Overall, the average score obtained at the Body awareness subscale and Supradiaphragmatic subscale was 5.22 (SD = 1.62) and 2.05 (SD = 1.96), respectively.

We examined Pearson’s correlations between BPQ scores and changes in heart rate, pain intensity, and pain unpleasantness in response to the *faster vs. slower* feedback conditions - our main contrast of interest which was a key driver of effects. No significant associations were found between either BPQ subscale and the change in heart rate (all p>0.05). Likewise, no correlations were observed between the BPQ subscales and changes in pain unpleasantness or pain intensity across these two conditions (all p>0.05).

Overall, these findings do not suggest a reliable association between individual differences in self-reported body perception and the perceptual or physiological effects of the experimental manipulation. Please note that the administration of this questionnaire was fully exploratory, as the current sample size is likely underpowered for robust individual difference analyses, which typically require substantially larger samples to detect meaningful associations (Hedge et al., 2018; Schönbrodt and Perugini, 2013).

### Experiment 2

Experiment 1 established that false accelerated cardiac feedback induced both an illusory increase in the perception of pain and a modulation in the pain-anticipatory related cardiac state (i.e. heart rate) towards the expected level of the noxious stimulus, based on the frequency rate of the cardiac feedback. Experiment 2 tests whether the same perceptual and physiological changes are observed if participants are exposed to non-interoceptive feedback (i.e. unrelated to biological human sounds), which should be less likely to induce expectations of threat. To address this question, we substituted the interoceptive cardiac feedback with an exteroceptive tone but kept the experiment otherwise identical. Thus, as in Experiment 1, participants’ pain intensity (i.e. Numeric Pain Scale), unpleasantness (i.e. Likert scale) ratings, and cardiac frequency (i.e. ECG) were collected both in a no-feedback as well as in a feedback phase, which could be either congruent, faster, or slower than participants’ individual heart rate recorded over the no-feedback phase. We reasoned that participants would have no expectation that an external acoustic sound signals a preparation of their body to a threatening, highly painful stimulus. Consequently, perceptual and cardiac modulations associated with the feedback manipulation should be reduced or eliminated with exteroceptive feedback.

#### ECG heart rate measurement

As in Experiment 1, we first tested whether participants’ heart rate was affected by the feedback manipulation, now using non-interoceptive stimuli, using a 1-factor repeated measures ANOVA with three feedback levels (faster, congruent, slower). In contrast to Experiment 1, and as hypothesized, no robust differences between the three conditions emerged, *F*(2, 56)=2.54, p=0.088, *ηp²*=0.083. Our main hypothesis test, which directly compares cardiac frequency in trials with faster and slower feedback revealed no robust differences either, *t*(28) = 1.48, p=0.317, *d*=0.19. A TOST equivalence test was conducted to determine whether the difference in heart rate between the *Incongruent Faster* and *Incongruent Slower* conditions was statistically equivalent within a range of *d*=–0.57 to *d*=0.57. The observed effect size was *d*=−0.19 (90% CI [-0.36,–0.03]). Both one-sided tests were significant, *t*(28) = 2.05, p=0.025; *t*(28) = –3.71, p<0.001, indicating statistical equivalence between conditions, relative to the smallest detectable differences in our design.

Note that, if anything, the induced difference in heart rate went in the numerically opposite direction than in Experiment 1, showing a heart rate *increase* after faster compared to slower exteroceptive feedback, in contrast to the decrease induced by interoceptive feedback cues. Indeed, comparing this increase to the equivalent change in Experiment 1 with a between-groups t-test revealed robust differences, *t*(61) = 2.187, p=0.033, *d*=0.553. Thus, while faster vs. slower interoceptive heart rate feedback led to compensatory changes in participants’ own heart rate; no such effects were obtained for exteroceptive feedback.

As in Experiment 1, we also investigated whether any differences were apparent when faster and slower feedback were compared to congruent feedback. No significant differences emerged for faster feedback *t*(28) = 1.202, p=0.239, *d*=0.22. However, the slower feedback induced a decrease, *t*(28) = 2.291, p=0.030, *d*=0.43, again showing the opposite pattern than in Experiment 1.

Finally, we checked whether the interoceptive and exteroceptive stimuli in Experiments 1 and 2 induced overall changes in heart rate response, but no such differences were apparent, *t*(61) = 0.140, p=0.889, *d*=0.035.

#### Subjective pain intensity ratings

Data were analyzed as in Experiment 1. We first tested whether pain intensity ratings were generally affected by the feedback manipulation, using a 1-factor repeated measures ANOVA with the levels (faster feedback, congruent feedback, slower feedback). Consistent with the results of the heart rate data, there was no robust difference, *F*(2, 56) = 2.54, p=0.088, *ηp²*=0.083. In particular, there was no difference in pain ratings between trials with faster and slower feedback, *t*(28) = 0.64, p=0.53, *d*=0.12, and a A TOST equivalence test was conducted to examine whether the difference in external NPS ratings between the *faster* and *slower* conditions was statistically equivalent within bounds of Cohen’s d = ±0.57. The observed effect size was *d*=−0.12 (89% CI [–0.34, 0.10]). Both one-sided tests were significant, *t*(28) = 2.43, p=0.011; *t*(28) = –3.33, p=0.001, indicating that the effect was statistically equivalent within the bounds of our experimental design.

When the difference in pain intensity ratings induced by faster and slower exteroceptive feedback was compared to that induced by interoceptive feedback in Experiment 1, a significant difference emerged, *t*(61) = 2.052, p=0.044, *d*=0.519. Thus, while faster (relative to slower) heart rate feedback increased pain ratings, delivering the same feedback through an exteroceptive stimulus induced no such changes.

Relative to congruent feedback, intensity ratings were increased for both faster and slower feedback, even though the difference was robust only for the faster feedback, *t*(28) = 2.12, p=0.043, *d*=0.39, not the slower cardiac feedback, *t*(28) = 1.58, p=0.13, *d*=0.29.

Finally, we checked whether the interoceptive and exteroceptive stimuli in Experiments 1 and 2 induced overall changes in pain intensity ratings, but no such differences were apparent, *t*(61) = 0.538, p=0.593, *d*=0.136.

#### Subjective pain unpleasantness ratings

**Figure 3.**
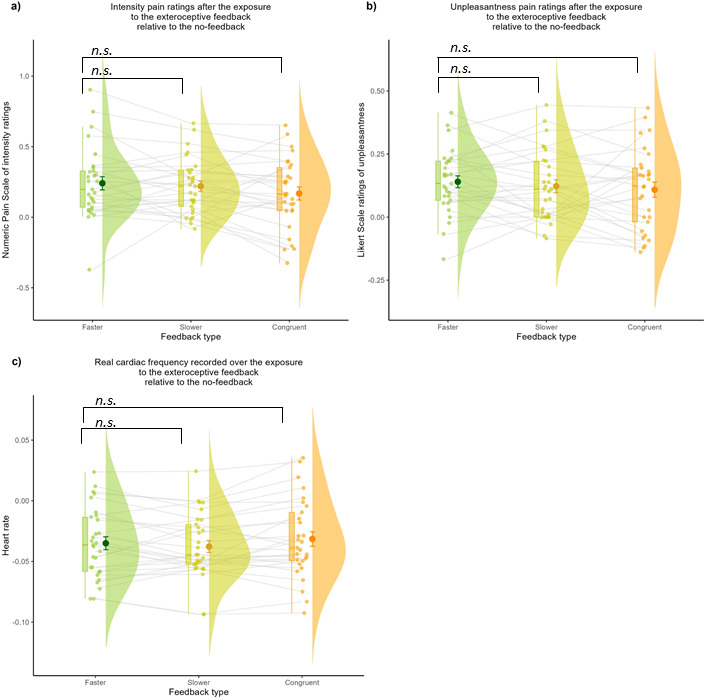
Exteroceptive feedback frequency does not induce interoceptive-like modulation of pain perception and anticipatory cardiac activity. (**a**) Intensity pain ratings (Numeric Pain Scale), (**b**) Unpleasantness ratings (5-point Likert Scale), (**c**) Real cardiac frequency. Values consist of the central tendency of change of pain intensity (**a**), unpleasantness (**b**), and heart rate (**c**) associated with the exposure to the faster, slower, and congruent exteroceptive feedback, relative to the no-feedback. Values of zero on the vertical axis would represent no change relative to the exposure to the no-feedback. Positive and negative values would represent an increase and a decrease, respectively. Brackets denote significance:=significant one-way RM-ANOVA; short bracket = planned Faster–Slower contrast. Asterisks indicate two-tailed p values (*<0.05, **<0.01, ***<0.001, “n.s.”=not significant).

Pain unpleasantness ratings showed the same pattern as pain intensity ratings. First, a 1-factor repeated measures ANOVA with the levels faster feedback, congruent feedback, and slower feedback revealed no robust differences, *F*(2, 56) = 1.01, p=0.371, *ηp²*=0.035. Moreover, there was no difference between trials with faster and slower feedback, *t*(28) = 0.864, p=0.395, *d*=0.16 (Figure 3b), and the A TOST procedure (Lakens, 2017) was conducted to test whether the difference in external liking ratings between the *faster* and *slower* conditions was statistically equivalent within bounds of Cohen’s d = ±0.57. The observed effect size was d=−0.02 (89% CI [–0.05, 0.02]). Both one-sided tests were significant, t(28) = 2.21, p=0.018; t(28) = –3.56, p<0.001, indicating statistical equivalence between the two conditions within the bounds of our experimental design.

Comparing these effects of exteroceptive feedback to the same stimulation delivered interoceptively in Experiment 1 revealed suggestive evidence for a difference in line with the previous measures, but it was not significant, *t*(61) = 1.679, p=0.098, *d*=0.424.

Further simple t-tests showed that, relative to the congruent feedback, differences did neither emerge for faster feedback, *t*(28) = 1.428, p=0.164, *d*=0.27, nor for the slower feedback, *t*(28) = 0.571, p=0.573, *d*=0.06.

Finally, we checked whether the interoceptive and exteroceptive stimuli in Experiments 1 and 2 induced overall changes in pain unpleasantness ratings, but no such differences were apparent, *t*(61) = 0.435, p=0.665, *d*=0.110.

#### Supplementary analyses

To rule out potential confounds and confirm the robustness of our findings, we conducted additional analyses on the raw data (see *Supplementary materials*), using the mixed linear models approach, which offer increased power and reliability with limited trials and conditions (Kliegl et al., 2010; Harald Baayen and Milin, 2010; Ambrosini et al., 2015). These analyses included all levels of feedback for heart rate and pain ratings (unpleasantness and intensity), stimulus intensity levels, as well as trial number (i.e. the rank order of each trial) as a variable to assess potential time-related effects such as learning, adaptation, or fatigue (e.g. Möckel et al., 2015). In addition, we incorporated the initial no-feedback phases as a condition to test for potential baseline differences, and we analyzed all stimulus intensities separately to test whether feedback effects were specific to particular nociceptive levels or generalized across them. These analyses fully replicate the findings of the main analysis reported above, but offer more detailed insights for interested readers into other, not theoretically relevant, factors that may affect the results. Due to the large number of comparisons, and the associated alpha inflation, these additional, not a priori predicted, results should be interpreted with caution, however, before being replicated in further studies.

## Discussion

Interoceptive inference and the Embodied Predictive Coding frameworks (Barrett, 2017; Barrett and Simmons, 2015; Pezzulo, 2014; Seth, 2013; Seth and Friston, 2016) propose that interoception is not merely a passive mirror of the internal milieu but rather the outcome of active, probabilistic inferences. Through this mechanism, the brain constantly estimates and regulates homeostatic states by integrating prior expectations with incoming interoceptive, proprioceptive, and exteroceptive signals. To test this framework in the context of pain, participants were given auditory cardiac feedback, which they believed reflected their actual physiological state, before the administration of pain stimuli. Unbeknownst to them, we varied the frequency of this heart rate feedback, making it either faster or slower. This manipulation aimed to create a false interoceptive prior, suggesting an increase or decrease in heart rate just before they received a painful stimulus.

In predictive models of interoception, such differences between expected and actual interoceptive information can be reduced by both changing one’s perceptual state to encompass the new information and by actively changing the body in response to the unexpected input, by engaging autonomous reflexes (i.e. perceptual and active inference, Parr et al., 2022; Friston, 2005; Friston, 2010; Ainley et al., 2016; Paulus et al., 2019). If so, then sensing an accelerated heart rate (relative to a decelerated one) should have two consequences: first, it may falsely lead participants to expect, and then perceive, a more intense pain stimulus than is really delivered. Second, it may induce changes in autonomic states themselves, compensating for the perceived changes in heart rate by actually down-regulating the real heart rate, towards the expected decreasing orienting cardiac response when expecting threat (Lykken et al., 1972; Taggart et al., 1976; Bradley et al., 2005; Bradley et al., 2008; Colloca et al., 2006; Tracy et al., 2017).

Experiment 1 provides evidence for both components of the hypothesized response. It showed, first, that faster cardiac feedback induced an illusory perception of increased unpleasantness and intensity of subsequent pain stimuli compared to slower cardiac feedback. Thus, as predicted, the mismatch between people’s expectations and the actual input led to changes in the perception of pain, towards the *expected* input associated with increased heart rates, in line with Bayesian/predictive processing accounts of pain perception. Second, false cardiac feedback also changed participants’ real heart rate. When hearing faster (relative to slower) feedback, the real heart rate decreased, assuming the pattern of anticipatory compensatory response typically enacted when humans prepare to face a threatening stimulus (Lykken et al., 1972; Taggart et al., 1976; Bradley et al., 2005; Bradley et al., 2008; Colloca et al., 2006; Tracy et al., 2017).

Importantly, both perceptual and cardiac frequency changes after faster (vs. slower) feedback were not observed when the heartbeat tones were replaced by exteroceptive sounds in Experiment 2. This finding supports the hypothesis that the observed perceptual and physiological modulations are specifically tied to interoceptive feedback, which provides evidence of the current state of the body in preparation for a potential upcoming (threatening) stimulus. In contrast, as the frequency of an external sound is not informative about the current state of the body, it should not induce any nociceptive stimulus expectations. Indeed, no discrepancy in the pain unpleasantness and intensity ratings, as well as in participants’ actual heart rates, were observed in Experiment 2. Importantly, the change from interoceptive to exteroceptive feedback did not induce any overall changes in pain perception or heart rate between experiments, only affecting the sensitivity to its rate (i.e. faster or slower). This suggests that the two types of cues were otherwise similarly processed, and did not vary substantially in overall arousal or sensitivity to pain they induced by themselves.

To our knowledge, this is the first study to successfully demonstrate that both *perceptual* and *active inference* components of pain are modulated through false interoceptive feedback. This provides evidence for the proposed mechanism through which, based on prior knowledge and expectations, the organism infers and actively implements the interoceptive consequences that have been predicted (Barrett and Simmons, 2015; Pezzulo, 2014; Seth and Friston, 2016). Our findings therefore support predictive coding accounts of interoception, which propose such effects on perception and bodily state, and extend them beyond exteroceptive cues (visual, Wiech et al., 2014; Colloca et al., 2006; Jepma et al., 2018; auditory, Colloca et al., 2006; Atlas et al., 2010). They indicate that cardiac signals are weighted in the brain’s generative model of pain and serve as key variables in allostatic regulation (Barrett et al., 2016), driving both the perception of further interoceptive signals and the regulation of adaptive bodily states.

A conceptual parallel can be drawn with the Rubber Hand Illusion (RHI), where mismatches between seen and felt touch are resolved both perceptually and through autonomic adjustments (Botvinick and Cohen, 1998; Moseley et al., 2008). Crucially, these responses can emerge from expectations alone (Ferri et al., 2013). Similarly, here, faster cardiac feedback created a prediction of threat that was resolved perceptually as higher pain ratings and actively as a parasympathetic deceleration of heart rate. Thus, just as the RHI illustrates predictive integration of visual and proprioceptive signals, the present findings reveal similar mechanisms in the visceral domain, showing that interoceptive predictions about cardiac state can reshape both the subjective and autonomic dimensions of pain.

An interesting observation was that, when compared to congruent cardiac feedback, the current effects on pain perception and autonomic responses were mainly driven by accelerated, not decelerated, feedback. Similar asymmetries of faster cardiac feedback have been reported in other domains. For instance, Iodice et al., 2019 showed that participants overestimate the effort they exerted when provided with faster cardiac feedback, whereas slower feedback had no effect. We speculate that, here, the asymmetry arises because increased heart rates are a biologically conserved (and culturally reinforced) cue for arousal, threat, and pain, which occurs for a wide range of stressors. Faster feedback therefore conveys the expectation of pain by mimicking the bodily response that follows its actual experience (i.e. heart-rate acceleration). In contrast, a slowed heart rate is the body’s intended autonomic response when *expecting* (but not yet experiencing) pain in both human and non-human species, to prepare for the forthcoming stressor (for reviews, see Livermore et al., 2021; Roelofs, 2017; Vila et al., 2007). It is considered an evolutionarily conserved, adaptive response that allows the organism to orient attention, enhance information processing, and action preparation (Hashemi et al., 2019; Klaassen et al., 2021; Lojowska et al., 2015). In our case, therefore, decelerated cardiac feedback signals that this intended autonomic response for dealing with the anticipated pain stimulus is already achieved. An accelerated heart rate, in contrast, signals the opposite, providing interoceptive evidence of a misaligned bodily state that mandates immediate allostatic responses, in terms of both pain perception and cardiac regulation (Barrett and Simmons, 2015; Pezzulo, 2014; Seth and Friston, 2016). Our findings therefore blend well with recent proposals that frame cardiac deceleration under a Bayesian inference approach. Under these views, the cardiac deceleration mechanism allows to adjust precision of sensory evidence accumulation relative to precision of bodily information, optimizing perception and action (Skora et al., 2022). Thus, decelerated cardiac activity when exposed to the faster feedback may reflect an autonomic adjustment to prioritize incoming noxious input information in light of the expectation of the threatening painful event.

Together, our evidence therefore supports the hypothesis that not only interoceptive states modulate interoceptive inference and perception, but the converse is also true: inference dynamics can modify or produce new internal states, to the extent that the actual cardiac frequency rate decreased as it would have done over the anticipation of a real threat. More broadly, our findings are in line with recent proposals of *interoceptive schemas* (Barca and Pezzulo, 2020; Iodice et al., 2019; Schoeller et al., 2024; Tschantz et al., 2022), internal models akin to the body schema (Head and Holmes, 1911), which consist of a central representation of interoceptive variables (e.g. body temperature, cardiac activity etc.) along with prior assumptions or ‘set points’ for these variables. This internal model would support homeostatic and allostatic regulation by optimally weighting multiple (i.e. proprioceptive, interoceptive, exteroceptive) streams of information to predict incoming interoceptive signals (Barca and Pezzulo, 2020; Iodice et al., 2019; Schoeller et al., 2024; Tschantz et al., 2022). Interoceptive illusions, that is contextual manipulations that shift the precision (i.e. estimated reliability) of interoceptive expectations (Owens et al., 2018; Schoeller et al., 2024), offer a direct probe of this schema. Our paradigm is one of such manipulation, showing how a transient change in cardiac evidence reveals how the precision weighting of priors and their prediction errors reshapes both the perceptual and autonomic dimensions of pain. Such illusions open a window onto the internal generative models that guide perception and action. In this regard, the contribution of the study of illusions offers important insights not only for a converging model of brain functioning under the principle of optimal predictive architectures, but it also provides implications for understanding psychopathology in terms of aberrant predictions and compounding allostatic consequences (for a full discussion, see Barrett and Simmons, 2015). Because manipulating contextual cues selectively alters the precision-weighting of interoceptive expectations, such illusions constitute the kind of diagnostic ‘stress tests’ that reveal how much confidence the brain assigns to priors versus bodily evidence, a parameter now believed to underlie dysfunctional conditions, such as anxiPTSD, and chronic pain (Schoeller et al., 2024). Our experimental paradigm provides a probe of this mechanism: the coupled shift in pain ratings and heart-rate deceleration indexes the degree to which threat-related priors dominate over incoming cardiac signals. Emerging perspectives (Schoeller et al., 2024) frame precisely this imbalance (i.e. excessively precise priors or under-weighted sensory channels) as a form of *false inference* that underlies psychopathology, and identify interoceptive illusions as tools both for phenotyping such aberrant precision control and for retraining it through repeated, controlled exposure.

### Accounting for general unspecific contributions

One possibility is that the different effects in Experiments 1 and 2 may not reflect interoceptive vs exteroceptive processing, but more general features of the sounds used in both experiments. For example, the heartbeat sounds’ cultural meaning and salience (e.g. as signals of anxiety or distress), or their acoustic properties (e.g. loomingness, roughness), could have increased arousal, alertness, or attentional engagement more generally. Several aspects of our data argue against this proposal, however. First, if heartbeat sounds had such general alerting/arousing effects, they should have induced robust between-experiment differences in pain ratings or heart rates when such heartbeat (Experiment 1) or non-heart related sounds were presented (Experiment 2). However, no such differences were obtained for any of our measures (see between-experiment t-tests in the main analyses; cross-experiment analysis in supplementary analyses). In contrast, all effects we obtained reflected not the overall presence or absence of cardiac feedback, but responses to a *change* in cardiac feedback (faster or slower), which was absent for identical changes in exteroceptive feedback.

Second, one may suspect that the acceleration of cardiac feedback, rather than cardiac feedback more generally, had simply increased arousal, instead of functioning as a misaligned interoceptive signal. However, if so, one would expect to see an overall *acceleration* in participants’ actual heart rate, as this is a typical physiological marker of heightened arousal (Azarbarzin et al., 2014; Wascher, 2021; Yang et al., 2017), not the slowing of the heart rate we had predicted and observed. Thus, our findings are *opposite* to what would be expected under a generic arousal-based explanation but consistent with the well-known anticipatory physiological regulatory response predicted by interoceptive inference frameworks. Note also that for such changes in heart rate to be obtained, very strong manipulations of threat or arousal are required. For example, in Parrotta et al., 2024, even highly salient and reliable threat cues (i.e. images that predicted upcoming pain with 100% certainty) did not produce any measurable change in heart rate. Our pattern of results is therefore not readily accounted for by general arousal or salience mechanisms, but is fully in line with the intrinsic capacity of cardiac feedback to engage autonomic regulation.

Finally, the idea that the interoceptive relevance of the cue, rather than its low-level acoustic properties or general human-related salience, is the key driver of the observed effects is also reflected in neurophysiological studies. For example, fMRI studies have shown that heartbeat sounds selectively activate interoceptive regions (e.g. anterior insula, frontal operculum; Kleint et al., 2015), while EEG work (Vicentin et al., 2024) has demonstrated enhanced cortical activation to faster heartbeats, particularly over frontocentral regions, suggesting enhanced processing in networks associated with interoceptive attention. Moreover, heartbeat sounds have been shown to attenuate the auditory N1 component (van Elk et al., 2014), a neural signature typically linked to self-generated or predicted bodily signals.

### Limitations and future directions

The current results provide a platform for further investigation of the link between perceptual and active inference in interoception. As this was the first study using our paradigm, it was optimized to detect robust within-subject effects of pain expectation. By definition, such robust within-subject effects minimize between-participant differences and are therefore difficult to link to cross-participant variability (i.e. the ‘reliability paradox’, Hedge et al., 2018), requiring substantially larger samples than used here and in the literature (i.e. N>200). Potentially informative associations, such as those between anticipatory heart-rate dynamics and pain ratings, could not be tested here. Similarly, the absence of a robust relationship between pain modulation and BPQ subscales of body awareness and supradiaphragmatic reactivity should be interpreted with caution, given that these measures were included for exploratory purposes and not sufficiently powered to draw strong conclusions, especially about the absence of relationships. Future work designed and powered for individual differences should determine how interoceptive awareness and cardiac attention shape pain experience. Such studies could also investigate how the current findings depend on sex/gender. In our sample, like in most psychology research, women were somewhat over-represented. Prior research suggests that men and women differ in pain processing and interoceptive sensitivity (Mogil, 2012; Prentice and Murphy, 2022). Future studies with larger samples could investigate whether such differences modulate the integration of interoceptive signals and pain perception, especially as predictive processing accounts assume that the precision of sensory information determines how much it is weighted against prior expectations (Yon and Frith, 2021).

A promising avenue is integrating perceptual, neuroimaging and physiological measures (e.g. the Heartbeat Evoked Potential, Petzschner et al., 2019) into computational models of interoceptive inference (e.g. Allen and Friston, 2018; Owens et al., 2018; Allen et al., 2022; Eckert et al., 2022). Similar inferential frameworks as proposed here have been implemented in computational models of cardiac (Smith et al., 2020) and gastrointestinal (Smith et al., 2021) interoception, thermoregulation (Tschantz et al., 2022), and psychopathological conditions, such as anorexia nervosa (Barca and Pezzulo, 2020), panic disorder (Maisto et al., 2021), self-injury (Barca et al., 2023), depression (Barrett et al., 2016; Stephan et al., 2016), and others (Paulus et al., 2019). Such frameworks would allow latent parameters like prior precision and sensory gain to be estimated for each participant (Unal et al., 2021), allowing us to test whether individuals who rely more heavily on cardiac priors also exhibit larger pain illusions and autonomic adjustments, further enabling individual differences research and extending it to clinical applications.

Model comparison could also establish whether faster cardiac feedback shifts the mean of the pain prior or instead alters the precision of nociceptive evidence. Systematically manipulating the precision of priors or prediction errors, or explicitly measuring participants’ interoceptive beliefs and their precision, offers a powerful means to uncover the mechanisms that facilitate the development of the interoceptive illusion. They may reveal whether perceptual biases in the experience of pain emerge from overlearned priors that do not update to incoming sensory data, or whether it is possible to directly act on interoceptive illusions and their autonomic consequences by changing prior knowledge.

The latter would have far-reaching implications, making it possible to both modify the perception of our internal milieu and impact autonomic states by simply reconfiguring individuals' beliefs and knowledge. Such findings would have profound consequences for understanding and potentially intervening in psychopathological conditions. On this basis, manipulating contextual cues can selectively up- or down-weight interoceptive expectations. In our paradigm, the coupled shift in pain ratings and heart-rate deceleration quantifies the extent to which threat-related priors override incoming bodily signals (i.e. cardiac input), a precision parameter that recent accounts frame as the very locus of dysfunctional predictive processes in anxiety, PTSD, and chronic pain.

From this perspective, the pain illusion documented here, together with other interoceptive illusions, provides a direct window into the brain’s predictive architecture (Barrett and Simmons, 2015), offering a mechanistic framework for understanding psychopathology as a failure to revise inaccurate interoceptive priors, leading to maladaptive regulation and compounding allostatic consequences. At the same time, they serve as diagnostic ‘stress tests’, revealing how much confidence the brain assigns to priors versus bodily evidence and thereby offering a mechanistic foothold for targeted intervention. Finally, we note that in our unpleasantness scale, the lowest anchor was labeled ‘no pain’. Although this label was not ideal, as unpleasantness and pain can in principle be dissociated, the effect of this choice is likely minimal given that unpleasantness ratings were always provided after intensity ratings, and our main findings rely on the interplay between intensity measures and physiological cardiac modulations.

### Conclusions

This study is the first to show that false cardiac signals reshape both pain perception and anticipatory autonomic responses. False cardiac feedback amplified pain intensity and unpleasantness while inducing parasympathetic deceleration of heart rate, revealing how expectations actively construct interoceptive experiences and drive allostatic adjustments. These findings support predictive models by emphasizing the pivotal role of interoceptive schemas in predictive architectures and highlight cardiac signals as key variables in the generative models governing pain and autonomic regulation. Importantly, the lack of effects with non-interoceptive feedback highlights the specificity of this mechanism to cardiac interoceptive signals, providing a platform to explore whether other visceral channels, such as respiratory cues, similarly recalibrate pain perception and physiological states. Beyond advancing theoretical models of interoception, our paradigm offers a novel tool for probing the precision-weighting of priors and sensory evidence in healthy and clinical populations, which is consistent with proposals that interoception provides actionable targets for mental-health interventions (Nord and Garfinkel, 2022). Future work may explore its translational potential in understanding and modulating maladaptive pain experiences, where aberrant interoceptive predictions contribute to chronic pain and related disorders.

## Methods

### Experiment 1

#### Participants

Thirty-five participants (mean age 25.11, SD = 2.94, 21 women) took part in the experiment, recruited from Gabriele D’Annunzio University and the wider community. All were right-handed with normal or corrected-to-normal vision. Exclusion criteria for taking part were self-reported chronic and acute pain, neurological disease, serious cardiovascular disease (i.e. any type of disease involving the heart or blood vessels that might result in life-threatening medical emergencies, e.g. arrhythmias, infarction, stroke), or conditions that could potentially interfere with pain sensitivity (e.g. drug intake or skin diseases). Participants were instructed not to drink coffee or smoke cigarettes. After taking part in the experiment, participants were excluded if the correlation between the desired five levels of the nociceptive stimulus intensity and their ratings in the no-feedback condition was below *r*=0.75, suggesting insensitivity to the varying nociceptive degrees of the electrical stimulus. No participants were excluded based on this criterion. All gave written informed consent, were unaware of the purposes of the study, and were fully debriefed about it at the end of the experiment.

The study was approved by the Ethical Committee of the Pescara and Chieti, Protocol Number 20016. All participants provided written informed consent to participate. Consent to publish was not separately required because no identifiable personal data was reported.

A sensitivity analysis with G*Power 3.1 (Faul et al., 2007) showed that the final sample size of 34 provides 0.90 power to detect effects with Cohen’s *d*=0.57 (SESOI of *δ*=0.34). Following recommendations, we do not estimate power based on previously reported effect sizes as this neglects uncertainty around reported effect size measurements, especially for new effects for which no reliable effect sizes can be estimated across studies (Albers and Lakens, 2018; Anderson et al., 2017). Instead, we report sensitivity analyses that reveal the effect sizes that the crucial comparison between faster and slower feedback is well-powered to detect, given our experimental parameters (i.e. target power, sample size, and type of test), as well as the smallest effect size of interest it can, in principle, reveal (SESOI, Lakens, 2022). Note that these values are well below the observed top-down effects on pain ratings of *d*=0.7 reported before (e.g. Iodice et al., 2019).

#### Apparatus

Painful stimuli were electrical pulses delivered using a constant-current electrical stimulator (Digitimer DS7A) controlling a pair of neurological electrodes attached to the phalanx of the middle finger of the participant’s left hand, which provide a precise constant current, isolated stimulus, controllable in pulse duration and amplitude. The duration of the electrical stimuli (2 ms) was kept fixed over the experiment and it was established during a calibration phase before the experiment.

Cardiac recording was performed by a Biopac MP 160 system (Biopac Systems Inc, USA). ECG was recorded continuously from two electrodes attached to the lower ribs and one over the right mid-clavicle bone (reference electrode). The ECG signal was sampled at 2 kHz with the Biopac Acqknowledge 3.7.1 software (Biopac Systems Inc, USA) according to the manufacturer’s guidelines. The ECG signal was then fully analyzed in MATLAB (R2020a). The tone used for the creation of the feedback was the sound of a single heartbeat, gathered from https://freesound.org and manipulated in Audacity. The feedback audio was then created in MATLAB (R2020a), repeating the single heartbeat sound according to the desired frequency (see *Procedure*).

Stimulus presentation was controlled through E-Prime (Psychology Software Tools Inc, Pittsburgh, USA), which interfaced with the pain stimulators and the Biopac system via a parallel port.

#### Procedure

Upon arrival at the lab, participants were briefed by the experimenter. After providing consent, they were placed in a comfortable chair with their left hand placed on a table, and the ECG electrodes were applied after cleaning the skin. To increase the ambiguity and thus predictive influences acting on the pain stimulus (Yon and Frith, 2021), the intensity of the noxious input varied between five intensities, which were identified for each participant in an initial calibration session. To do so, participants were informed that they would undergo a psychophysical calibration procedure to determine their subjective response to increasing stimulus intensities. The first stimulus was delivered at a low intensity, which is below the threshold for pain perception in most people. The intensity increased in a ramping procedure up to a maximum of five volts. Participants verbally rated the pain intensity for each stimulus on a 0–100 Numerical Pain Scale (NPS).

Following previous research (Atlas et al., 2014; Colloca et al., 2006; Hird et al., 2019), a pain intensity rating of NPS 20 denoted ‘just painful’, NPS 50 denoted ‘medium pain’, and NPS 80 marked the point at which the stimulus was ‘just tolerable’. We identified a ‘low’ pain level of 10, a ‘low-medium’ pain level of 30, a ‘medium’ pain level of 50, a ‘medium-high’ pain level of 70, and a ‘high’ pain level of 90. Level 100 was considered the point where the participant did not wish to experience a higher stimulation level in the experimental session and was not used. We repeated this procedure three times and computed the average stimulus intensities over these three repetitions corresponding to NPSs 10, 30, 50, 70, and 90.

Participants then underwent a pre-experiment test procedure: stimulus intensities corresponding to their pain intensity ratings NPS 10–90 were delivered in a pseudo-randomized order four times and participants were instructed to identify the intensity of each pulse. Participants had to correctly identify 75% of stimulus intensities to proceed to the main experiment. If they did not achieve this in the test procedure, the intensities were adjusted, and the test was repeated until participants correctly identified 75% of stimulus intensities (Hird et al., 2019). Participants were excluded if the correlation between their ratings of the five levels of the nociceptive stimulus intensities and the actual stimulation intensities within the baseline phase was below *r*=0.75, suggesting insensitivity to the varying nociceptive degrees of the electrical stimulus, and an essentially flat response profile. No participants were excluded with this criterion. This procedure was needed because, although the paradigm introduced ambiguity by using different levels of actual stimulus intensity, it was important to ensure that participants developed a stable internal representation of the pain scale and to ensure that illusion effects were not due to an inability to reliably distinguish between intensities. Note that this correlation criterion only requires that all participants’ ratings generally increase with actual stimulus intensities; participants are still free to vary in absolute values and the range they use on the pain scale. Appendix 1—figure 11 shows the simulation intensities corresponding to each of the five pain levels obtained through this calibration.

Having established the individual levels of intensity for each stimulation, participants were informed that their task was to rate the intensity and unpleasantness of nociceptive stimulations. We carefully described the distinction between intensity and unpleasantness ratings using the standard language developed by Price et al., 1989, emphasizing that pain intensity and unpleasantness should be rated independently.

The experiment started with a no-feedback session in which participants’ heart rate in anticipation of the shock (measured with ECG) and perceived level of pain (measured with Numeric Pain Scale and Likert scale) were assessed. These measures were used to normalize our dependent variables for our analysis (see *Data analysis*).

Participants were seated in front of the computer, with the ECG electrodes attached, their left hand placed on the table with the electrodes of the cutaneous electrical stimulation fixed on the phalanx of the middle finger, and their right hand placed on the mouse, ready to start the task. The ECG was recorded for the entire session.

Each trial of the no-feedback phase started with the presentation of a fixation cross appearing on the screen. After 60 s, a single electrical shock was administered, randomly varying the level of intensity in each trial - as established in the previous calibration phase (i.e. NPS 10, 30, 50, 70, and 90). After each stimulation, participants first rated the intensity of the painful stimulus (instruction: ‘How intense was the painful stimulation?’) on a continuous Numerical Pain Scale (NPS) appearing on the screen, from 0 (‘not at all painful’) to 100 (‘extremely painful’). They then rated the unpleasantness of the painful stimulus (instruction: ‘How unpleasant was the painful stimulation?’) on a 5-point Likert scale (1=no pain, 2=weak, 3=moderate, 4=severe, 5=extremely severe). The two scales were presented on the screen in succession, and participants made their judgments through a mouse click on the scales. Between trials, a pause screen was shown for 45 s, in order to avoid the subsequent trial to be contaminated by the heart rate response to the shock. Then, a new screen appeared with the instructions to press the spacebar to start the new trial.

The no-feedback phase comprised 10 trials, after which participants were asked to wait and rest for 15 min before participating in the second experimental feedback phase. This time was used by the experimenter to prepare the acoustic stimuli for use in the second session for each participant. For this purpose, we assessed the individual mean heart rate during the anticipation of the painful stimulus by measuring the mean R-R intervals of the ECG for the whole 1 min interval preceding the shock, and by transforming them into frequency (1/R-R, Colloca et al., 2006). The congruent feedback consisted of tones of heartbeats reproduced at the same individual HR frequency recorded in the no feedback phase. The incongruent faster and slower feedback consisted of tones of heartbeats reproduced at a frequency corresponding to an R-R interval obtained by either reducing or increasing the length of the original mean R-R interval over the no-feedback phase of 25%, respectively. The choice of a±25% variation in heart rate feedback was guided by prior research using false cardiac feedback paradigms. Studies in this field have used a range of modulation strategies, from fixed-frequency symbolic feedback (e.g. Valins, 1966; Tajadura-Jiménez et al., 2008) to real-time individualized adjustments without specific percentage values (e.g. Iodice et al., 2019). More recent work has adopted proportional manipulations of instantaneous heart rate, using variations such as –20% (Azevedo et al., 2017), ±30% (Dey et al., 2018), and ± 50% (Gray et al., 2007). Our choice of ±25% falls within this established range, aiming to strike a balance between producing a salient modulation and preserving the plausibility of the feedback as a reflection of one’s actual physiology. Specifically, the generation of feedback was tailored to each participant. The feedback was set to be 25% above or below their baseline heart rate, with the feedback gradually increasing or decreasing. This individualized approach ensured that each participant experienced feedback relative to their own baseline heart rate. Moreover, to make the incongruent feedback manipulation credible, the incongruent acoustic feedback always started at a frequency rate that reproduced the individual’s heart rate recorded over the no feedback phase, to then gradually increase or decrease the R-R length according to whether the feedback was slower or faster, respectively. In the feedback phase, participants were informed that their task was similar to the previous no-feedback procedure, namely, to rate the intensity and unpleasantness of pain stimuli. They were also informed that in the 60 s preceding the administration of the shock, they would hear acoustic feedback, which was equivalent to their ongoing heart rate. This phase consisted of 18 trials (six trials per experimental condition) of 60 s (i.e. fixation) each, after which the electrical shock was administered, randomly varying its intensity in each trial (i.e. NPS 30, 50, and 70), either congruent, slower, or faster than the participant’s heart rate recorded in the no-feedback phase.

This relatively low number of repetitions was a deliberate design choice based on both theoretical and practical considerations. As observed in other body illusion paradigms (e.g. Botvinick and Cohen, 1998; Ehrsson, 2007; Pratviel et al., 2022), repeated exposures can reduce illusion strength due to habituation, decreased plausibility, or increased awareness of the manipulation. Moreover, sessions lasted approximately 1.5–2 hr and involved cognitively demanding tasks, limiting the feasibility of increasing trial numbers without introducing participant fatigue or disengagement. Finally, as we used explicit pain ratings - measures typically less noisy than implicit physiological indices - fewer trials were sufficient to detect reliable effects.

As for the use of only these three pain intensities in the test phase, the rationale was to focus on a manageable subset that still covered a meaningful portion of the stimulus spectrum. Following the approach of Iodice et al., 2019, PNAS, we deliberately excluded the extreme calibrated levels (NPS 10 and NPS 90) to avoid floor- and ceiling-effects. Moreover, by restricting the range we ensured that every test intensity could be paired with both a ‘slower’ and a ‘faster’ cardiac-feedback condition derived from an adjacent level - pairings that would have been impossible at the extremes where no neighbouring level exists.

Specifically, each of the three levels of intensity of the nociceptive stimulus was associated with one of the three different types of acoustic feedback from the previous no-feedback phase. As in the previous no-feedback phase, participants’ individual mean heart rate before the painful stimulus (i.e. ECG) and the perceived intensity and unpleasantness of pain (i.e. NPS and Likert scale) were assessed. The order of the experimental conditions (i.e. congruent, slower, and faster) was randomly generated for each participant by a web-based computer program (www.randomization.com).

#### Body Perception Questionnaire

After the experiment, all participants completed the Body Perception Questionnaire (Short form BPQ-SF; Poli et al., 2021) to investigate whether either perceptual or autonomic modulation as induced by our task would be predictive of self-reported measures of bodily awareness and reactivity. The Body Perception Questionnaire is a 22-item self-administered questionnaire that assesses awareness and reactivity of the autonomic nervous system, that is, the subjective ability to perceive bodily states and bodily reactions to stress. High scores on the BPQ reflect high awareness of internal bodily signals (i.e. high interoceptive sensibility) and high perceived reactivity of the visceral nervous system. Items ask participants to rate, on a 5-point scale (from 1=never to 5=always), the frequency with which they feel aware of bodily sensations (e.g. body awareness subscale ‘My mouth being dry’), experience supradiaphragmatic reactivity (e.g. supradiaphragmatic reactivity subscale ‘I feel shortness of breath’), and subdiaphragmatic reactivity (e.g. subdiaphragmatic reactivity subscale reactivity subscale ‘I have indigestion’). In this work, we focus on the body awareness and supradiaphragmatic reactivity subscales, following prior research (Petzschner et al., 2019). Body awareness plays a critical role in how individuals perceive and interpret bodily signals, which in turn affects emotional regulation and self-awareness. Supradiaphragmatic reactivity refers specifically to organs located or occurring above the diaphragm - such as the heart - compared to subdiaphragmatic reactivity subscales further down. Our decision to include these subscales was further supported by recent work from Unal et al., 2021, showing that supradiaphragmatic reactivity predicts attentional modulation of the heartbeat-evoked potential (HEP), an electroencephalographic signature of the heartbeat. Thus, this subscale, and the more general body awareness scale, most closely reflect the interplay between cardiac attention, bodily awareness, and the development of the interoceptive illusion.

#### Data analysis

Measures of the heart rate recorded with the ECG (beats per minute) in the feedback phase were normalized relative to the baseline (i.e. no-feedback phase) by subtracting and then dividing by the baseline (Lambert et al., 2024; Mirmoosavi et al., 2024; Sundararaj et al., 2025) using the formula: normalized value = (X - bX)/bX, where X represents the mean value of each measure assessed in the experimental feedback phase and bX the mean value of the measure calculated over the no-feedback phase. As in prior research (e.g. Bartolo et al., 2013; Cecchini et al., 2020; Riello et al., 2019), the same normalization was also applied to pain intensity and unpleasantness measures. We did not have specific hypotheses about how the different levels of noxious stimulus intensity affect the perceptual illusion.

For all three dependent variables (heart rate, pain intensity, and pain unpleasantness), our main hypothesis is tested by comparing the effects of faster against the effects of slower feedback. Statistically, this comparison collapses our prediction onto a single and most powerful test of our hypothesis for each of the dependent measures, as faster and slower feedback are assumed to emerge from changes in opposite directions, in contrast to separate comparisons against the congruent feedback for which effect sizes will by necessity be on average half of the combined influence of the faster vs slower comparison. Moreover, as the slower and faster feedback manipulation reflect analogous deviations from the real heart rate, this comparison keeps any effect of a perceived absolute deviation (in either direction) from the real heart rate constant. Assume, for example, that in one participant, heart rates generally decrease as soon as a deviation from the real heart rate is noticed (i.e. in either direction). The comparison between faster and slower feedback will still measure whether this decrease is larger for faster than slower feedback, just as in a participant who does not show such a general decrease or one who shows an increase.

The data from each of our dependent measures (heart rate, pain intensity ratings, pain unpleasantness ratings) were analyzed similarly. First, to confirm that the relevant measure of interest was affected by the different types of heart rate feedback, the data were entered into a 1-factor repeated measures ANOVA (Greenhouse-Geisser corrected) with the levels faster feedback, congruent feedback, and slower feedback.

Second, once such an influence was confirmed, a planned paired t-test tested our main prediction: that there would be a difference in objective heart rate as well as subjective measures of pain intensity and pain unpleasantness between trials with faster and slower feedback.

Finally, we tested whether any such effect was mostly driven by the faster instead of the slower feedback, using separate t-tests comparing the faster feedback against the congruent feedback and the slower feedback against the congruent feedback.

Please note that, as noted above, this latter comparison is not necessary to test our hypothesis, which only requires a difference between faster and slower feedback. Indeed, many influential studies omitted such a control entirely (Hill et al., 2024; Makkar and Grisham, 2013; Phillips et al., 1999; Valins, 1966), or if included, typically treat it as a baseline control (e.g. Patchitt et al., 2025). Nevertheless, we included the congruent condition here to provide an informative baseline reference, and situating our findings clearly within the broader experimental context, which suggests a specific role of heart rate deviations upwards (i.e. faster) than downwards (slower; Iodice et al., 2019). For all analyses, the assumption of normality was systematically assessed using the Kolmogorov-Smirnov (K-S) test.

### Experiment 2

#### Participants

Thirty-five participants (mean age 25.17, SD = 4.02, 23 women) were recruited from Gabriele D’Annunzio University and the wider community. All were right-handed with normal or corrected-to-normal vision. Exclusion criteria were identical to Experiment 1. All gave written informed consent, were unaware of the purposes of the study, and were fully debriefed at the end of the experiment. Ethical approval from the local ethics board was obtained.

As in Experiment 1, participants were excluded if the correlation between the desired five levels of the nociceptive stimulus intensity and their ratings in the no-feedback condition was below *r*=0.75, suggesting insensitivity to the varying nociceptive degrees of the electrical stimulus. Five participants were excluded with this criterion. Moreover, participants were excluded if, for any reason, they linked the exteroceptive sound to a simulation of an interoceptive, cardiac acoustic feedback, in their post-experiment debriefing. One additional participant was excluded with this criterion. Hence, the final sample size was 29. A sensitivity analysis with G*Power 3.1 (Faul et al., 2007) showed that a sample size of 29 provides 0.90 power to detect effects with Cohen’s *d*>0.62 (SESOI of *δ*=0.37).

#### Apparatus and stimuli

The apparatus and stimuli were the same as Experiment 1. The only difference was the selection of the tone used to present the heart rate feedback. Instead of using a heartbeat, the sound was a single percussion obtained by knocking two woods, gathered from https://freesound.org and manipulated in Audacity. The feedback audio was then created in MATLAB (R2020a), repeating the single tone according to the desired frequency (see *Procedure*).

#### Procedure

The procedure used in Experiment 2 was the same as Experiment 1, with the only exception that the sound used for the feedback was an exteroceptive, instead of interoceptive (i.e. cardiac) tone. Unlike in Experiment 1, where participants were told they would hear their own heartbeats in real time, in Experiment 2 participants were not informed that the sound was meant to represent their heart rate.

#### Data analysis

Data analysis was identical to Experiment 1. Note that we predicted an absence/reduction of effects here, compared to the findings of Experiment 1. In standard analyses, a non-significant result does not allow one to conclude that there is no effect; it simply indicates a lack of evidence for a statistically detectable difference. The predicted reduction/absence of effects with exteroceptive cues here relative to the interoceptive cues in Experiment 1 were tested in two ways.

First, to test for the predicted absence of effects with exteroceptive cues, equivalence tests (i.e. TOST ‘two one-sided t-tests’ procedure; Lakens, 2017; Lakens et al., 2018) were performed for the differences between each dependent (i.e. pain unpleasantness, pain intensity, heart rate). The TOST procedure addresses the above limitation by assessing whether a given (typically non-significant) effect is smaller than the effective smallest effect size of interest (SESOI) that the current design has power to detect, using two one-sided t-tests against positive and negative equivalence bounds. If both tests are statistically significant, it confirms that the differences in heart rate, pain intensity, and pain unpleasantness induced by the target conditions (faster and slower feedback) are smaller than the smallest effect size of interest detectable in the current study.

Second, to confirm that the exteroceptively induced changes in heart rate and pain perception differ from those with interoceptive stimuli in Experiment 1, we compared our main contrast of interest - each participant’s difference between faster and slower feedback in each of the three dependent measures - between the two experiments, with independent-samples t-tests. A significant result from this t-test shows that the changes induced by faster vs slower exteroceptive feedback here differ from the analogous changes induced by interoceptive feedback in Experiment 1.

Finally, we checked whether the different sounds (interoceptive heartbeat sounds in Experiment 1, exteroceptive control sounds in Experiment 2) induced any overall changes in heart rates, painfulness, or unpleasantness, which would be expected if these sounds differ systematically in the amount of arousal or alertness they induce, using between-group t-tests on overall heart rate, painfulness, or unpleasantness averaged across all feedback conditions (equivalent to a main effect of Experiment in an ANOVA).

## Figures and Tables

**Appendix 1—figure 1.**
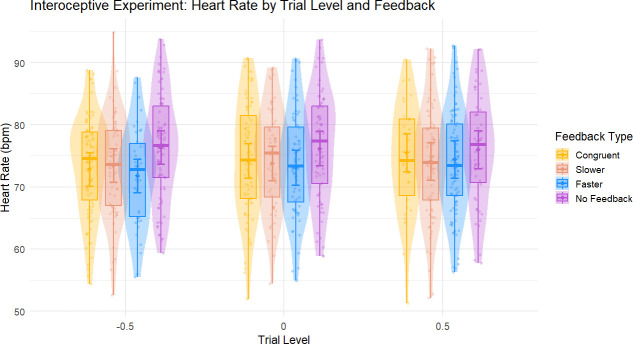
Interoceptive experiment: Model-predicted heart rate (HR) responses as a function of standardized trial progression (Trial) across feedback conditions (Feedback ×Trial was significant, F(3, 711.19) = 6.16, p<0.001). The Trial variable is modeled as a continuous, mean-centered predictor capturing temporal dynamics within the session (from –0.5=early trials to +0.5 = late trials). Violin plots show the distribution and density of predicted HR values at early, middle, and late trial points.

**Appendix 1—figure 2.**
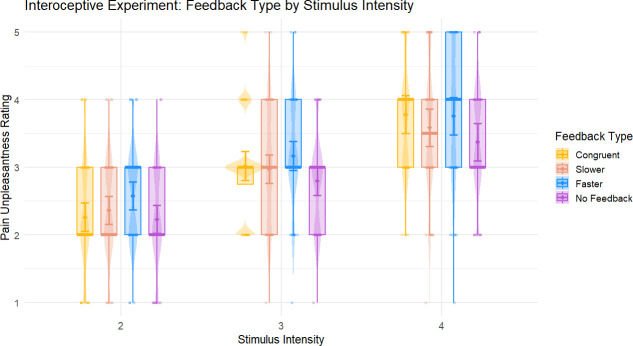
Interoceptive experiment: Model-predicted Pain Unpleasantness ratings for each stimulation intensity (StimInt 2–4), across feedback conditions (Stimulus Intensity × Feedback was significant, F(3, 672.32) = 3.90, p=0.0088). Violin plots and overlaid boxplots depict the distribution (violin) and interquartile range with median (box) of the observed data for each condition. Boxes are centered on the x-axis categories because they summarize the data within each stimulation intensity and feedback condition. Large colored dots and error bars show the model-predicted estimated marginal means (EMMs)± standard errors for each condition.

**Appendix 1—figure 3.**
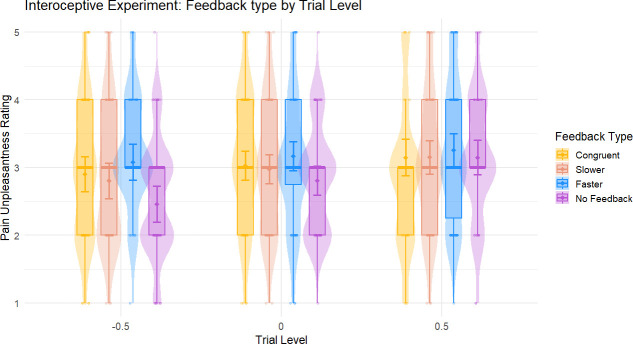
Interoceptive experiment: Model-predicted pain unpleasantness ratings as a function of standardized trial progression (Trial) across feedback conditions (Feedback × Trial, *F*(3, 675.33)=3.44, p=0.0165). The Trial variable is modeled as a continuous, mean-centered predictor reflecting the temporal progression of the task (from –0.5=early trials to +0.5 = late trials). Violin plots display the distribution and density of predicted ratings at early, middle, and late stages of the session.

**Appendix 1—figure 4.**
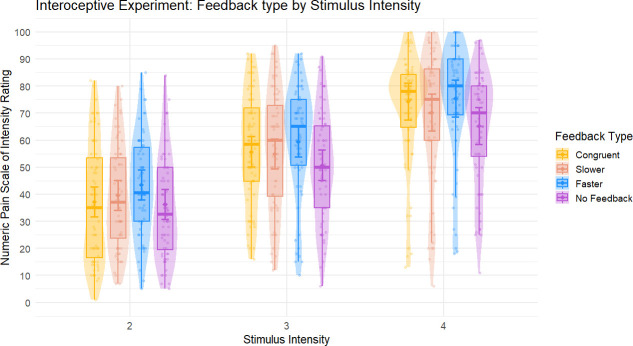
Interoceptive experiment: Model-predicted Numeric Pain Scale of intensity ratings (NPS) for each stimulation intensity (StimInt 2–4), across feedback conditions (Feedback × Stimulus Intensity, F(3, 667.07) = 3.4631, p=0.016). Violin plots and overlaid boxplots depict the distribution (violin) and interquartile range with median (box) of the observed data for each condition. Boxes are centered on the x-axis categories because they summarize the data within each stimulation intensity and feedback condition. Large colored dots and error bars show the model-predicted estimated marginal means (EMMs)± standard errors for each condition.

**Appendix 1—figure 5.**
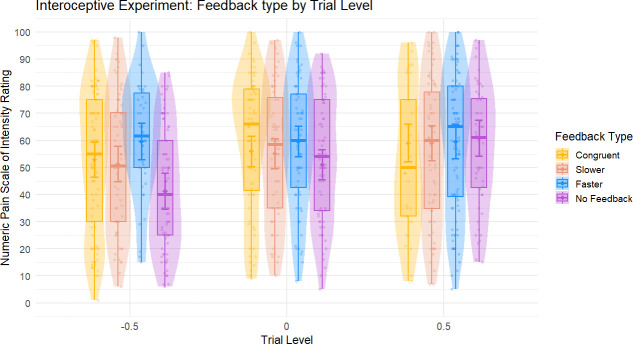
Interoceptive experiment: Model-predicted Numeric Pain Scale of intensity ratings (NPS) as a function of standardized trial progression (Trial) across feedback conditions (Feedback × Trial, *F*(3, 669.15)=9.86, p<0.001). The Trial variable is modeled as a continuous, mean-centered predictor reflecting the temporal progression of the task (from –0.5=early trials to +0.5 = late trials). Violin plots display the distribution and density of predicted ratings at early, middle, and late stages of the session.

**Appendix 1—figure 6.**
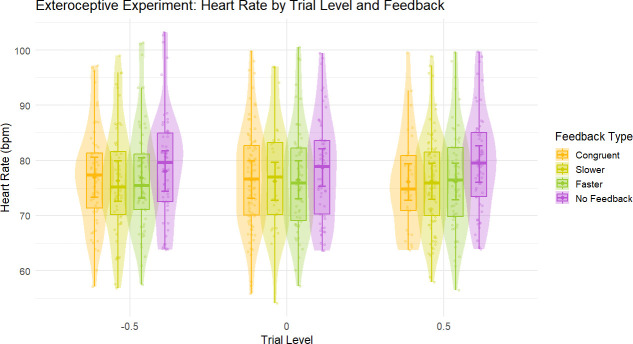
Exteroceptive experiment: Model-predicted heart rate (HR) responses as a function of standardized trial progression (Trial) across feedback conditions in the Exteroceptive condition. The Trial variable is modeled as a continuous, mean-centered predictor capturing temporal dynamics within the session (from –0.5=early trials to +0.5 = late trials). Violin plots show the distribution and density of predicted HR values at early, middle, and late trial points. Feedback x Trial was not significant; values are shown for consistency with the interoceptive experiment.

**Appendix 1—figure 7.**
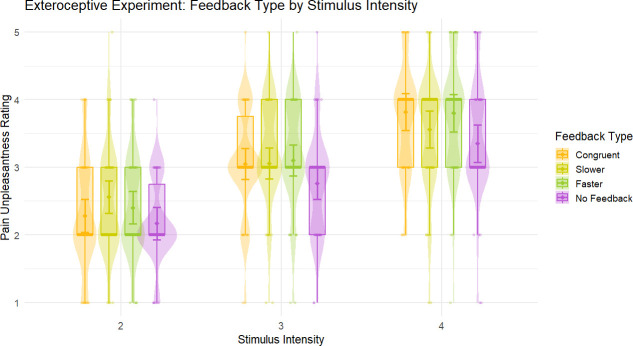
Exteroceptive experiment: Model-predicted pain unpleasantness ratings for each stimulation intensity (StimInt 2–4), across feedback conditions (Stimulus Intensity ×Feedback, F(3, 569.16) = 8.21, p<0.0001). Violin plots and overlaid boxplots depict the distribution (violin) and interquartile range with median (box) of the observed data for each condition. Boxes are centered on the x-axis categories because they summarize the data within each stimulation intensity and feedback condition. Large colored dots and error bars show the model-predicted estimated marginal means (EMMs)± standard errors for each condition*.*

**Appendix 1—figure 8.**
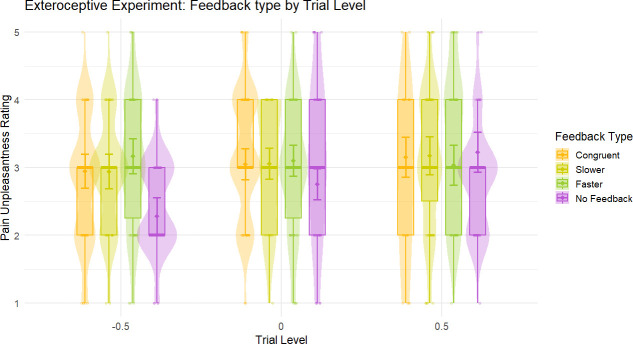
Exteroceptive experiment: Model-predicted pain unpleasantness ratings as a function of standardized trial progression (Trial) across feedback conditions (Feedback ×Trial, *F*(3, 568.85)=11.47, p<0.0001). The Trial variable is modeled as a continuous, mean-centered predictor reflecting the temporal progression of the task (from –0.5=early trials to +0.5 = late trials). Violin plots display the distribution and density of predicted ratings at early, middle, and late stages of the session.

**Appendix 1—figure 9.**
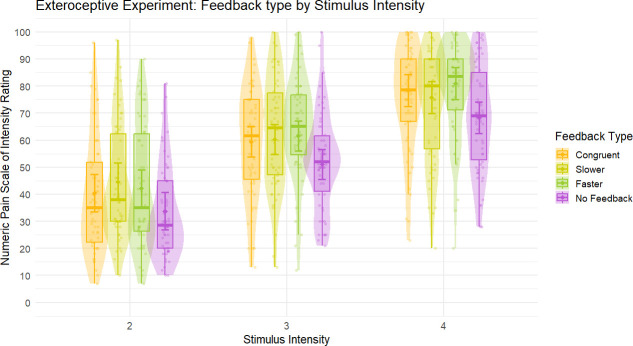
Exteroceptive experiment: Model-predicted Numeric Pain Scale of intensity ratings (NPS) for each stimulation intensity (StimInt 2–4), across feedback conditions (Feedback × Stimulus Intensity, F(3, 570.65) = 3.00, p=0.0301). Violin plots and overlaid boxplots depict the distribution (violin) and interquartile range with median (box) of the observed data for each condition. Boxes are centered on the x-axis categories because they summarize the data within each stimulation intensity and feedback condition. Large colored dots and error bars show the model-predicted estimated marginal means (EMMs)± standard errors for each condition.

**Appendix 1—figure 10.**
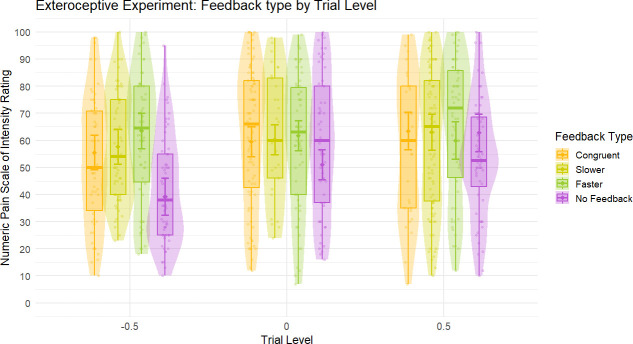
Exteroceptive experiment: Model-predicted Numeric Pain Scale of intensity ratings (NPS) as a function of standardized trial progression (Trial) across feedback conditions (Feedback ×Trial, *F*(3, 569.80)=12.13, p<0.001). The Trial variable is modeled as a continuous, mean-centered predictor reflecting the temporal progression of the task (from –0.5=early trials to +0.5 = late trials). Violin plots display the distribution and density of predicted ratings at early, middle, and late stages of the session.

**Appendix 1—figure 11.**
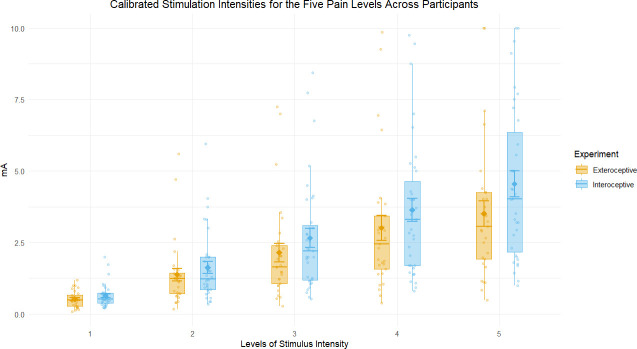
Individually calibrated stimulation intensities (in mA) associated with five subjectively defined pain levels (NPS 10, 30, 50, 70, and 90), shown separately for the Exteroceptive and Interoceptive experiments. Each violin plot includes the distribution of individual values, boxplots with interquartile range and median, and the mean ± standard error.

## Data Availability

Anonymized raw ECG recordings underlying the results reported in this study are publicly available on OSF at: https://doi.org/10.17605/OSF.IO/5SW3M. Anonymized trial-level behavioural data, ECG-derived physiological measures, and analysis code are publicly available at: https://github.com/EP171993/painperception (copy archived at Parrotta, 2026). The following dataset was generated: ParrottaE 2026Exposure to false cardiac feedback alters pain perception and anticipatory cardiac frequencyOpen Science Framework10.17605/OSF.IO/5SW3MPMC1326864642294597

## Additional files

MDAR checklist

## Appendix 1

### Supplementary material

#### Linear mixed-effects model

We used linear mixed-effects models to investigate the effects of Feedback, Stimulus Intensity, and Trial on our dependent measures (Numeric Pain Scale intensity ratings, Likert ratings, and heart rate). In all models, the variable Trial was included as a continuous predictor reflecting the relative position of each trial within the session. Trial was linearly rescaled within each participant to range from –0.5 (first trial) to +0.5 (last trial), using min–max normalization. This transformation enables consistent interpretation of temporal effects across sessions of different lengths and aligns with best practices in linear mixed-effects modeling (Meteyard and Davies, 2020; Schad et al., 2020).

Stimulus Intensity (StimInt) was originally derived from three discrete stimulation levels (3, 4, and 5), corresponding to subjective pain ratings of NPS 30, 50, and 70, respectively. These levels were selected to induce variability in pain perception while avoiding floor and ceiling effects, in line with previous work on pain illusions (Atlas et al., 2014; Colloca et al., 2006; Hird et al., 2019). For statistical modeling, these levels were recoded as a continuous centered variable: –0.5 (low = NPS 30), 0 (medium = NPS 50), and +0.5 (high = NPS 70), allowing for linear trend analysis.

Feedback was modeled as a categorical factor with four levels (Congruent, Slower, Faster, No Feedback), and Experiment (Exp) was included as a fixed factor in between-subject models comparing the interoceptive and exteroceptive conditions (Exteroceptive, Interoceptive).

To increase the robustness of our analyses, we excluded outliers based on standardized residuals: observations with residuals exceeding ±2 SDs were removed prior to model fitting (Harald Baayen and Milin, 2010). Residual diagnostics were then performed by visually inspecting quantile–quantile (Q–Q) plots to assess the normality assumption.

Random effects structures included by-subject random intercepts and random slopes for both Trial and Stimulus Intensity (StimInt), following recommendations for maximizing model generalizability (Barr et al., 2013). For each model, we compared the full random-effects structure to simpler models using likelihood ratio tests, and retained random slopes only when they significantly improved model fit (see also Matuschek et al., 2017).

We did not include *Experiment* (Exp) or *Feedback* as random effects in our models. *Experiment* was a between-subjects factor reflecting two distinct, theoretically defined groups (Interoceptive vs. Exteroceptive). Because participants were nested within a single experiment, this factor lacked within-subject variability and was therefore modeled as a fixed effect. This approach is consistent with standard practices for modeling between-subject manipulations that represent specific experimental conditions rather than a sample from a broader population (Barr et al., 2013; Brauer and Curtin, 2018).

Similarly, *Feedback* was modeled as a fixed effect rather than a random factor. This within-subject manipulation comprised four experimentally designed levels (Congruent, Slower, Faster, No Feedback), each corresponding to a distinct prediction condition derived from our theoretical framework. These levels do not represent a random sample from a larger population of feedback types, but rather a set of conditions defined a priori based on predictive coding and interoceptive inference models. As such, modeling *Feedback* as a random factor would violate key assumptions of hierarchical modeling, while also obscuring the interpretation of fixed effects and their interactions. In line with best practices in mixed-effects modeling (Barr et al., 2013; Matuschek et al., 2017), random effects should be specified only for factors whose levels are sampled from a larger population, which was not the case in our design.

While some modeling approaches adopt a stepwise procedure, adding or removing predictors to assess incremental improvements in model fit, our models were not constructed in this way. Instead, we specified a full-factorial fixed-effects structure a priori, in order to comprehensively test whether the originally observed effects of feedback on pain ratings and heart rate remained robust when including all levels of Feedback (including No Feedback and Congruent) and all levels of the effective stimulus intensity. Although these analyses go beyond the scope of the original confirmatory models, they were conducted in part in response to a reviewer’s request for a more detailed examination of variability across trials and conditions. Their structure, however, was determined by theoretical considerations rather than by data-driven model selection. This approach aligns with best practices in mixed modeling that prioritize hypothesis-driven model specification and theoretical transparency over purely exploratory, stepwise procedures (Gelman and Hill, 2007; Barr et al., 2013; Harrell, 2015). Moreover, stepwise model building can be useful in exploratory contexts, but it has also been criticized for inflating Type I error rates, encouraging overfitting, and obscuring interpretability (Harrell, 2015). In contrast, a priori model specification allows for clearer hypothesis testing, maintains the integrity of confirmatory analyses, and ensures that all theoretically relevant terms, including interactions, are directly modeled. Accordingly, our models included the full factorial structure of fixed effects necessary to test the hypothesized interactions (e.g. Exp × Feedback × Trial), and random effects were tested and retained based on improvements in model fit via likelihood ratio tests (Matuschek et al., 2017).

All models were fitted using restricted maximum likelihood estimation (REML), and p-values were computed using Satterthwaite’s approximation of degrees of freedom. For each fixed effect, we report the estimated coefficient (b), SE, t-statistic (t), and p-value (p).

### 1. Cross-experiment analysis (between-subjects model)

#### 1.1. Heart rate

To examine heart rate (HR) variations across experimental conditions, we applied general linear mixed-effects modeling (GLMM), which accounts for both between- and within-participant variability by estimating fixed effects while allowing subject-specific random variations. The model was fitted using **R** (lme4 package; Bates et al., 2015) and was specified as follows:

$$\displaystyle HR\sim Experiment \times Feedback\times Trial+(Trial|Subject)$$

where Experiment, Feedback, and Trial were included as fixed effects. A random slope for Trial was included at the subject level (Trial | Subject) to account for individual differences in temporal HR changes. Model comparison confirmed that including the random slope for Trial significantly improved model fit (χ²=11.462, p=0.003), indicating that the trajectory of HR changes over time varied across participants.

#### 1.1.2 Results

##### 1.1.2.1 Effects of interest

The results of the Type III ANOVA with Satterthwaite’s approximation revealed a significant *main effect of Feedback*, *F* (3,1305.28) = 102.68, p<0.001, indicating that HR varied across feedback conditions. Compared to the reference condition (i.e. Congruent feedback), HR was significantly higher in the No Feedback condition (*b*=1.97, SE = 0.24, *t*=8.21, p<0.001), significantly lower in the Faster feedback condition (*b*=−1.10, SE = 0.24, *t*=−4.55, p<0.001), and showed a non-significant decrease in the Slower feedback condition (*b*=−0.42, SE = 0.24, *t*=−1.76, p=0.079). *Post hoc comparisons* further clarified these effects. HR was significantly lower in the Faster feedback condition compared to Congruent feedback (p=0.0008), and significantly higher in the No Feedback condition compared to all other conditions (p<0.0001 for all comparisons). The Slower feedback condition did not differ significantly from the Faster condition (p=0.216), but showed a significant reduction in HR compared to the No Feedback condition (p<0.0001). These results suggest that the absence of feedback elicited the highest HR, while faster feedback was associated with the lowest HR.

Crucially, the *Feedback × Experiment* interaction was also significant, F(3, 1305.28) = 3.58, p=0.013, indicating that the effect of Feedback on HR differed between the Interoceptive and Exteroceptive experiments.

Post hoc comparisons confirmed that in the *Interoceptive experiment*, heart rate (HR) in the No Feedback condition (*M*=76.2, SE = 1.45) was significantly higher than in all other feedback conditions. Specifically, HR was significantly higher than in the Faster condition (*M*=73.1, SE = 1.45; estimate = –3.07, SE = 0.24, *t*(1310.3) = –12.94, p<0.0001), in the Slower condition (*M*=73.8, SE = 1.45; estimate = –2.38, SE = 0.23, *t*(1305.5)=–10.31, p<0.0001), and in the Congruent condition (*M*=74.2, SE = 1.45; estimate = –1.96, SE = 0.24, *t*(1307.1) = –8.16, p<0.0001). Importantly, HR in the Faster condition was significantly lower than in the Slower condition (estimate = 0.68, SE = 0.23, *t*(1309.2) = 2.92, p=0.0043) and than in the Congruent condition (estimate = 1.10, SE = 0.24, *t*(1307.6) = 4.55, p<0.0001).

In the *Exteroceptive experiment*, HR in the No Feedback condition (*M*=78.8, SE = 1.57) was also significantly higher than in all other feedback conditions. It was significantly higher than in the Congruent condition (*M*=76.6, SE = 1.57; estimate = –2.22, SE = 0.25, *t*(1305.9) = –8.74, p<0.0001), the Slower condition (*M*=76.2, SE = 1.57; estimate = –2.58, SE = 0.25, *t*(1306.4) = –10.26, p<0.0001), and the Faster condition (*M*=76.5, SE = 1.57; estimate = –2.33, SE = 0.25, *t*(1306.0)=–9.25, p<0.0001). However, in contrast to the Interoceptive experiment, no significant differences emerged between the Congruent, Slower, and Faster feedback conditions (Congruent vs. Slower: estimate = 0.36, SE = 0.25, *t*(1302.4)=1.45, p = 0.2204; Congruent vs. Faster: estimate = 0.11, SE = 0.25, *t*(1302.5)=0.42, p=0.674; Slower vs. Faster: estimate = –0.26, SE = 0.25, *t*(1301.6) = –1.04, p = 0.3566).

Importantly, the crucial difference in HR between the Slower and Faster feedback conditions also differed significantly across experiments (*b*=0.941, SE = 0.34, *t*=2.76, p=0.005). Specifically, the Slower–Faster contrast was larger in the Interoceptive experiment, indicating a more pronounced reduction in HR from the Slower to the Faster condition when participants were exposed to the interoceptive relative to the exteroceptive feedback. Moreover, interaction contrasts also revealed that the difference in HR between the Faster and No feedback conditions varied significantly across experiments, with a greater HR reduction in the Interoceptive experiment compared to the Exteroceptive one (b=–0.7411, SE = 0.346, t=–2.145, p=0.032). No other between-experiment differences reached significance. Finally, interaction contrasts revealed that the difference in HR between the Faster and Congruent feedback conditions varied significantly across experiments (*b*=0.998, SE = 0.35, *t*=2.86, p=0.004), with a greater HR reduction in the Interoceptive experiment compared to the Exteroceptive one.

##### 1.1.2.2 Additional unpredicted effects

Due to the large number of comparisons the results here should be treated with caution before being replicated.

Although the *main effect of Trial* did not reach significance, F(1, 64.44) = 3.51, p=0.066, the fixed-effect estimate indicated a general increase in HR across trials (b=2.89, SE = 0.71, t=4.08, p<0.001).

A significant interaction between *Trial × Experiment* emerged, *F*(1, 64.44) = 5.65, p=0.020, indicating that the trajectory of heart rate (HR) over time differed between the two experiments. HR increased significantly across trials in the Interoceptive experiment, *b*=1.40, SE = 0.45, 95% CI [0.51, 2.29]. In contrast, no significant change in HR over time was observed in the Exteroceptive experiment, *b* = –0.17, SE = 0.48, 95% CI [–1.13, 0.80]. The difference between these temporal trends was statistically significant, *b*=1.57, SE = 0.66, *t*(64.8) = 2.38, p=0.020, with a more positive HR slope in the Interoceptive than in the Exteroceptive experiment.

Moreover, the three-way interaction between *Trial × Feedback × Experiment* was significant, *F*(3, 1309.10) = 9.44, p<0.001, indicating that the trajectory of HR over time depended on both feedback type and experimental condition.

In the *Interoceptive experiment*, HR significantly increased over trials in the Congruent feedback condition (*b*=2.89, SE = 0.71, 95% CI [1.50, 4.29], *t*=4.07, p=0.0001), as well as in the Faster condition (*b*=2.66, SE = 0.64, 95% CI [1.41, 3.92], *t*=4.171, p<0.0001). In contrast, no significant increase was observed in the Slower condition (*b*=0.66, SE = 0.61, 95% CI [–0.55, 1.86], *t*=1.072, p=0.285), and HR even showed a non-significant decreasing trend in the No Feedback condition (*b* = –0.61, SE = 0.72, 95% CI [–2.03, 0.80], *t*=–0.853, *p=*0.3940). Post hoc contrasts confirmed that the HR increase in the Congruent condition was significantly greater than in both No Feedback (estimate = 3.51, SE = 0.89, *t*(1320)=3.95, p=0.0003) and Slower feedback (estimate = 2.24, SE = 0.80, *t*(1307) = 2.79, p=0.0103), but did not differ significantly from Faster feedback (estimate = 0.23, SE = 0.82, *t*(1313) = 0.28, p=0.78).

In the *Exteroceptive experiment*, HR showed a non-significant decreasing trend over trials in the Congruent condition (*b* = –1.13, SE = 0.68, 95% CI [–2.46, 0.20]), and remained flat in the Slower condition (*b* = –0.001, SE = 0.65, 95% CI [–1.29, 1.29]). Similarly, HR did not significantly change over time in the Faster condition (*b* = –0.82, SE = 0.74, 95% CI [–2.28, 0.64]) nor in the No Feedback condition (*b*=1.28, SE = 0.81, 95% CI [–0.30, 2.87]). However, post hoc contrasts showed that the No Feedback condition was associated with a significantly different HR trajectory compared to Congruent feedback (*estimate* = –2.42, SE = 0.91, *t*(1310) = –2.65, p=0.049), while no other significant differences emerged between Congruent and Slower (p=0.227) or Faster feedback (p=0.714).

Finally, interaction contrasts on the HR slope revealed several significant differences in feedback-related trajectories across the two experimental contexts.

The difference in HR slope between Congruent and No Feedback conditions was significantly larger in the Interoceptive experiment than in the Exteroceptive one (*b*=5.92, SE = 1.27, *t*=4.65, p<0.0001). Specifically, HR increased over time in the Congruent condition but remained flat or even decreased in the No Feedback condition, and this contrast was markedly stronger in the Interoceptive context.

A similar interaction was found for the comparison between Faster and No Feedback (*b*=5.38, SE = 1.27, *t*=4.23, p<0.0001), indicating that the presence of temporally accelerated feedback boosted HR increase significantly more in the Interoceptive experiment than in the Exteroceptive one.

Additionally, the contrast between Slower and No Feedback was also significantly greater in the Interoceptive context (*b*=2.56, SE = 1.21, *t*=2.12, p=0.0345). Beyond these comparisons with No Feedback, additional interaction effects were observed among active feedback conditions.

The contrast between Slower and Faster feedback differed significantly across experiments (*b*=–2.82, SE = 1.12, *t*=–2.52, p=0.0117), reflecting a stronger HR increase under Faster than under Slower feedback in the Interoceptive experiment, a pattern not observed in the Exteroceptive experiment.

The contrast between Congruent and Slower was also significantly greater in Interoceptive compared to Exteroceptive experiment (*b*=3.37, SE = 1.12, *t*=3.01, p=0.0027), while no significant difference emerged between Congruent and Faster feedback conditions across experiments (*b*=0.54, SE = 1.19, *t*=0.46, p=0.647).

#### 1.2 Likert Pain Unpleasantness Ratings Scale

To examine variations in pain unpleasantness ratings across experimental conditions, we applied general linear mixed-effects modeling (GLMM), which accounts for both between- and within-participant variability. The model was fitted using the lmer function from the *lme4* package (Bates et al., 2015) in R and specified as follows:

$$\displaystyle \begin{array}{ll}\text{LIKERT PAIN UNPLEASENTNESS RATINGS} \sim \\{\rm Experiment}\times {\rm Feedback}\times {\rm StimInt} \times{\rm Trial}+(\rm StimInt+\,Trial\,|Subject) \end{array}$$

Experiment, Feedback, StimInt, and Trial were included as fixed effects. Random slopes for Stimulus Intensity and Trial were specified at the subject level to account for individual variability. Model comparison confirmed that including these random slopes significantly improved model fit (χ²=117.73, p<0.001).

##### 1.2.1 Results

###### 1.2.1.1 Effects of interest

The Type III ANOVA revealed a significant *main effect of Feedback*, F(3, 1228.10) = 40.30, p<0.001, indicating that unpleasantness ratings differed across feedback conditions. Compared to the Congruent condition (reference), unpleasantness ratings were significantly higher in the Faster feedback condition (b=0.14, SE = 0.048, t(1231.07)=2.87, p=0.004), and significantly lower in the No Feedback condition (b = –0.23, SE = 0.047, t(1228.15) = –4.97, p<0.0001). The Slower condition did not differ significantly from the Congruent condition (p=0.17). Post hoc comparisons further clarified these effects. Likert pain unpleasantness ratings were significantly higher in the Faster feedback condition compared to Congruent feedback (p=0.0096), Slower feedback (p=0.0006), and No Feedback (p<0.001). The Slower feedback condition did not differ significantly from the Congruent condition (p=0.415). Moreover, the No Feedback yielded a reduction in Numeric Pain Unpleasantness ratings compared to all other feedback conditions (all p<0.0001).

Although the *Feedback × Experiment interaction* did not reach conventional significance in the omnibus ANOVA (F(3, 1228.10)=2.32, p=0.074), the results suggested a trend indicating that the effect of feedback on pain unpleasantness may differ between the Interoceptive and Exteroceptive experiments. Given that this was one of the main hypotheses of the study, we conducted follow-up analyses to further this interaction.

In the *Interoceptive experiment*, unpleasantness ratings in the No Feedback condition (M=2.80, SE = 0.105) were significantly lower than in all other feedback conditions. Specifically, ratings were significantly lower than in the Faster condition (M=3.17, SE = 0.105; estimate = –0.37, SE = 0.046, t(1232) = –7.97, p<0.0001), the Slower condition (M=2.97, SE = 0.105; estimate = –0.17, SE = 0.046, t(1232) = –3.66, p=0.0004), and the Congruent condition (M=3.03, SE = 0.105; estimate = –0.23, SE = 0.047, t(1229) = –4.95, p<0.0001). Importantly, unpleasantness ratings in the Faster condition were significantly higher than in the Slower conditions (p<0.0001), as well as in the Congruent (p=0.0051), and, while no significant difference emerged between the Congruent and Slower conditions (p=0.17).

In the *Exteroceptive experiment*, unpleasantness ratings in the No Feedback condition (M=2.75, SE = 0.113) were again significantly lower than in all other feedback conditions. Ratings in the No Feedback condition were significantly lower than in the Faster (M=3.10, SE = 0.113; estimate = –0.34, SE = 0.049, p<0.0001), Slower (M=3.06, SE = 0.113; estimate = –0.30, SE = 0.049, p<0.0001), and Congruent conditions (M=3.05, SE = 0.113; estimate = –0.29, SE = 0.050, p<0.0001). In contrast to the Interoceptive experiment, no significant differences emerged between the Congruent, Slower, and Faster feedback conditions (all ps >0.52).

To directly compare the feedback-related differences across experiments, interaction contrasts were computed.

Importantly, the contrast between Slower and Faster feedback was significantly more pronounced in the Interoceptive than in the Exteroceptive experiment (b = –0.16, SE = 0.067, t = –2.43, p=0.015), indicating a greater differentiation in unpleasantness between these conditions when the feedback was interoceptive. Similarly, the contrast between Slower and No Feedback was also significantly larger in the Interoceptive context (b = –0.14, SE = 0.067, t = –2.01, p=0.0445). No other feedback comparisons showed significant interaction effects between experiments (all ps >0.18).

###### 1.2.1.2 Additional unpredicted effects

A Type III ANOVA revealed a significant *main effect of Trial* on pain unpleasantness ratings, F(1, 64.50) = 19.63, p<0.001, suggesting that unpleasantness ratings varied throughout the experimental session. However, the fixed-effect estimate of the Trial coefficient did not reach statistical significance (b=0.27, SE = 0.15, t(269.04) = 1.76, p=0.079), indicating a non-significant trend toward increasing unpleasantness ratings over time. This apparent discrepancy reflects the fact that Trial was involved in several higher-order interactions, particularly with Stimulus Intensity and Feedback, that modulated its effect on unpleasantness ratings. These interactions are reported in detail below.

There was also a significant *main effect of Stimulus Intensity (StimInt)* on unpleasantness ratings, F(1, 62.30) = 352.15, p<0.001. Specifically, unpleasantness ratings increased significantly with higher stimulation levels, as confirmed by a strong and significant positive coefficient (b=1.52, SE = 0.12, t(172.73) = 12.82, p<0.0001), indicating that higher stimulus intensity reliably evoked greater unpleasantness.

Additionally, the effect of trial on pain unpleasantness ratings varied as a function of the actual intensity of the nociceptive stimulation, as indicated by a significant *Stimulus Intensity × Trial* interaction, F(1, 1253.50) = 17.84, p<0.001. To further investigate this interaction, estimated marginal trends of unpleasantness ratings across trials were computed at three levels of stimulus intensity: low (–0.5), medium (0), and high (+0.5). Unpleasantness ratings increased progressively over trials at all intensity levels, but the rate of increase became steeper as stimulus intensity increased.

At low intensity, the slope was positive but did not reach statistical significance (b=0.12, SE = 0.09, 95% CI [–0.06, 0.30], t(123.4) = 1.32, p=0.191). A significant positive trend emerged at medium intensity (b=0.34, SE = 0.08, 95% CI [0.19, 0.49], t(64.6) = 4.43, p<0.0001) and was even more pronounced at high intensity (b=0.56, SE = 0.09, 95% CI [0.37, 0.74], t(147.4) = 5.90, p<0.0001).

Pairwise comparisons between slopes (FDR-corrected) confirmed that the increase in unpleasantness ratings over trials was significantly steeper at higher stimulus intensity levels. Specifically, the slope at low intensity was significantly lower than both the medium (estimate = –0.22, SE = 0.052, t(1254) = –4.22, p<0.0001) and high-intensity levels (estimate = –0.44, SE = 0.10, t(1254) = –4.22, p<0.0001). Moreover, the slope at medium intensity was also significantly lower than at high intensity (estimate = –0.22, SE = 0.052, t(1254) = –4.22, p<0.0001).

The *Feedback × Stimulus Intensity* interaction was also significant, F(3, 1237.96)=10.50, p<0.001, indicating that the effect of stimulus intensity on pain unpleasantness ratings varied across feedback conditions. To further this interaction, estimated marginal means (EMMs) of unpleasantness ratings were computed at the three levels of stimulus intensity: low (–0.5), medium (0), and high (+0.5).

At *low stimulus intensity* (–0.5), the Faster feedback condition elicited the highest unpleasantness ratings (M=2.49, SE = 0.08), followed closely by Slower (M=2.47, SE = 0.08), Congruent (M=2.28, SE = 0.08), and No Feedback (M=2.20, SE = 0.08). Ratings in the Faster condition were significantly higher than those in the Congruent (estimate = –0.22, SE = 0.06, t(1238) = –3.92, p=0.0002) and No Feedback condition (estimate = 0.29, SE = 0.05, t(1235)=5.39, p<0.0001). Likewise, Slower feedback elicited significantly higher ratings than both Congruent (estimate = –0.19, SE = 0.05, t(1236) = –3.50, p=0.0007) and No Feedback (estimate = 0.26, SE = 0.05, t(1233)=4.98, p<0.0001). However, the difference between Congruent and No Feedback did not reach significance (p=0.2213)

At *medium intensity* (0), the Faster feedback produced the highest unpleasantness ratings (M=3.13, SE = 0.08), followed by Congruent (M=3.04, SE = 0.08), Slower (M=3.01, SE = 0.08), and No Feedback (M=2.78, SE = 0.08). Post hoc comparisons revealed that ratings were significantly higher in the Faster condition compared to both Congruent (estimate = –0.09, SE = 0.03, t(1229) = –2.66, p=0.0096) and Slower (estimate = –0.12, SE = 0.03, t(1228) = –3.53, p=0.0006). All three active feedback conditions also elicited significantly higher ratings than No Feedback (all ps <0.0001).

At *high intensity* (+0.5), unpleasantness ratings were highest under the Congruent feedback (M=3.80, SE = 0.10), followed by the Faster (M=3.77, SE = 0.10), Slower (M=3.56, SE = 0.10), and No Feedback (M=3.35, SE = 0.10). Ratings in the No Feedback condition were significantly lower than those in all other conditions (all ps <0.0001), and ratings in the Faster condition were significantly higher than Slower (estimate = –0.21, SE = 0.05, t(1232) = –3.99, p=0.0001). Congruent also differed significantly from Slower (estimate = 0.25, SE = 0.05, t(1232)=4.54, p<0.0001), whereas no significant difference emerged between Congruent and Faster (p=0.5377).

Unpleasantness ratings increased significantly as a function of stimulus intensity in all feedback conditions. However, the rate of increase (slope) varied depending on feedback type. The steepest increase was observed in the Congruent condition (b=1.53, SE = 0.09, t(164) = 17.76, p<0.0001), followed by Faster (b=1.28, SE = 0.08, t(154) = 15.10, p<0.0001), No Feedback (b=1.14, SE = 0.08, t(148) = 13.68, p<0.0001), and Slower (b=1.09, SE = 0.08, t(149) = 12.99, p<0.0001). Pairwise comparisons between slopes revealed that the slope was significantly steeper in the Congruent condition compared to Slower (estimate = 0.44, SE = 0.08, t(1239)=5.17, p<0.0001), Faster (estimate = 0.25, SE = 0.09, t(1239)=2.93, p=0.0069), and No Feedback (estimate = 0.38, SE = 0.08, t(1237)=4.52, p<0.0001). The difference between Faster and Slower also reached significance (estimate = 0.19, SE = 0.08, t(1239)=2.25, p=0.0367), whereas other comparisons did not (all ps >0.13).

The interaction between *Feedback × Trial* was significant, F(3, 1239.19)=14.39, p<0.0001, indicating that the trajectory of unpleasantness ratings across trials varied depending on the type of feedback received.

To further unpack this interaction, the estimated slopes of Trial were computed for each feedback condition. Unpleasantness ratings increased significantly across trials in both the Congruent (b=0.24, SE = 0.11, 95% CI [0.03, 0.45], t(235) = 2.22, p=0.0272) and Slower feedback conditions (b=0.29, SE = 0.10, 95% CI [0.10, 0.48], t(168) = 2.96, p=0.0036), indicating a moderate but consistent increase in perceived unpleasantness over time. In contrast, no significant change was observed in the Faster condition (b=0.01, SE = 0.10, 95% CI [–0.20, 0.22], t(221) = 0.08, p=0.9339). The steepest and most robust increase in unpleasantness ratings over time was found in the No Feedback condition (b=0.82, SE = 0.11, 95% CI [0.59, 1.04], t(286) = 7.22, p<0.0001).

Post hoc pairwise comparisons of slopes (FDR-corrected) confirmed that the rate of increase in unpleasantness over trials was significantly greater in the No Feedback condition compared to all other feedback types (all ps <0.0001). Specifically, the slope in the No Feedback condition was significantly greater than in the Congruent (estimate = –0.58, SE = 0.13, t(1245) = –4.56, p<0.0001), Slower (estimate = –0.53, SE = 0.12, t(1240) = –4.45, p<0.0001), and Faster conditions (estimate = –0.81, SE = 0.13, t(1242) = –6.44, p<0.0001). Additionally, the slope in the Faster condition was significantly lower than both the Congruent (p=0.0682, marginal) and Slower conditions (estimate = –0.28, SE = 0.11, t(1236) = –2.50, p=0.0186), while no significant difference emerged between the Congruent and Slower conditions (p=0.6562).

To further explore how the effect of feedback on unpleasantness evolved over time, estimated marginal means were computed at three levels of the standardized Trial variable (–0.5, 0, +0.5), representing early, middle, and late stages of the session, respectively. This analysis allowed us to assess whether the differences between feedback conditions were stable or changed over time.

At *early trials (*Trial = –0.5), unpleasantness ratings were significantly higher in the Faster condition (M=3.13, SE = 0.09) compared to both the Congruent (M=2.92, SE = 0.09; estimate = –0.21, SE = 0.07, t(1234) = –3.10, p=0.0024) and Slower conditions (M=2.87, SE = 0.09; estimate = –0.26, SE = 0.07, t(1231) = –3.81, p=0.0002). The No Feedback condition elicited the lowest ratings (M=2.37, SE = 0.10), significantly lower than all other feedback conditions (all ps <0.0001).

At *middle trials* (Trial = 0), the pattern remained consistent: Faster feedback yielded significantly higher ratings (M=3.13, SE = 0.08) compared to Congruent (M=3.04, SE = 0.08; estimate = –0.09, SE = 0.03, t(1229) = –2.66, p=0.0094) and Slower (M=3.01, SE = 0.08; estimate = –0.12, SE = 0.03, t(1227) = –3.55, p=0.0006), while the No Feedback condition (M=2.77, SE = 0.08) was again significantly lower than all other conditions (all ps <0.0001).

By *late trials* (Trial = 0.5), feedback-related differences in unpleasantness ratings had disappeared. No significant differences were found among any of the feedback conditions (all ps >0.90), and ratings converged: Congruent (M=3.16, SE = 0.10), Slower (M=3.15, SE = 0.09), Faster (M=3.14, SE = 0.09), and No Feedback (M=3.18, SE = 0.10). This pattern suggests that, as the session progressed, the modulatory effect of feedback on unpleasantness diminished.

#### 1.3 Numeric Pain Scale of intensity ratings

To examine variations in pain intensity rcatings (Numeric pain scale of intensity ratings) across experimental conditions, we applied general linear mixed-effects modeling (GLMM), which accounts for both between- and within-participant variability by estimating fixed effects while allowing subject-specific random variations. The model was fitted using R (lme4 package; Bates et al., 2015) and was specified as follows:

$$\displaystyle \begin{array}{ll}\text{NUMERIC PAIN SCALE OF INTENSITY RATINGS} \sim\\ \mathrm{Experiment} \times \mathrm{Feedback} \times \mathrm{StimInt} \times \mathrm{Trial} + (\mathrm{StimInt} + \mathrm{Trial} \mid \mathrm{Subject})\end{array}$$

where Experiment, Feedback. StimInt, and Trial were included as fixed effects. A random slope for StimInt and Trial was included at the subject level (StimInt +Trial | Subject) to account for individual differences in pain perception across stimulus intensities and over time. Model comparison confirmed that including these random slopes significantly improved model fit (χ²=134.14, p<0.001). The statistical significance of predictors was assessed using t-tests with Satterthwaite’s approximation to degrees of freedom. For each predictor, we report the estimated coefficient (b), standard error (SE), t-statistic (t), and p-value (p).

##### 1.3.1 Results

###### 1.3.1.1 Effects of interest

The results of the Type III ANOVA with Satterthwaite’s approximation revealed a significant *main effect of Feedback*, F(3,1228.44)=54.41, p<0.001, indicating that pain intensity ratings varied across feedback conditions. Compared to the reference condition (i.e. Congruent feedback), pain ratings were significantly lower in the No Feedback condition (b=–4.67, SE = 1.08, t=–4.31, p<0.001) and significantly higher in the Faster feedback condition (b=3.88, SE = 1.10, t=3.53, p<0.001). The Slower feedback condition showed no significant difference compared to the Congruent (p=0.53). Post hoc comparisons further clarified these effects. Numeric pain scale of intensity ratings were significantly higher in the Faster feedback condition compared to Congruent feedback (p=0.0002), Slower feedback (p=0.0002), and No Feedback (p<0.001). The Slower feedback condition did not differ significantly from the Congruent condition (p=0.8722). Moreover, the No Feedback yielded a reduction in Numeric pain scale of intensity ratings compared to all other feedback conditions (all p<0.0001).

Crucially, the *Feedback × Experiment interaction* was also significant, F(3, 1228.44)=4.48, p=0.004, indicating that the effect of Feedback on pain ratings differed between the Interoceptive and Exteroceptive experiments.

In the *Interoceptive experiment*, pain ratings in the No Feedback condition (*M*=51.1, SE = 2.65) were significantly lower than in all other feedback conditions. Specifically, pain ratings were significantly lower than in the Faster condition (*M*=59.6, SE = 2.66; *estimate* = –8.55, SE = 1.07, *t*(1234) = –7.99, p<0.0001), the Slower condition (*M*=55.1, SE = 2.65; *estimate* = –3.99, SE = 1.05, *t*(1231) = –3.79, p=0.0002), and the Congruent condition (*M*=55.8, SE = 2.66; *estimate* = –4.70, SE = 1.08, *t*(1230) = –4.34, p<0.0001). Importantly, pain ratings in the Faster condition were significantly higher than in the Slower condition (*estimate* = –4.56, SE = 1.07, *t*(1233) = –4.26, p<0.0001), and in the Congruent condition (*estimate* = –3.86, SE = 1.10, *t*(1234) = –3.51, p=0.0006). No significant difference was observed between the Slower and Congruent feedback conditions (estimate = 0.71, SE = 1.08, *t*(1230)=0.65, p=0.515).

In the *Exteroceptive experiment*, pain ratings in the No Feedback condition (*M*=51.0, SE = 2.87) were also significantly lower than in all other feedback conditions. Specifically, pain ratings were lower than in the Congruent condition (*M*=59.5, SE = 2.87; *estimate* = –8.48, SE = 1.15, *t*(1231) = –7.35, p<0.0001), the Slower condition (*M*=60.5, SE = 2.87; *estimate* = –9.44, SE = 1.14, *t*(1230) = –8.24, p<0.0001), and the Faster condition (*M*=61.8, SE = 2.87; *estimate* = –10.73, SE = 1.14, *t*(1230) = –9.41, p<0.0001). In contrast to the Interoceptive experiment, no significant differences emerged between the Congruent, Slower, and Faster feedback conditions (Congruent vs. Slower: *estimate* = –0.96, SE = 1.15, *t*(1229) = –0.83, p=0.406; Congruent vs. Faster: *estimate* = –2.25, SE = 1.15, *t*(1229) = –1.96, p=0.075; Slower vs. Faster: *estimate* = –1.29, SE = 1.14, *t*(1230) = –1.13, p=0.309).

Importantly, interaction contrasts revealed a significant difference in the effect of Slower vs. Faster feedback across experiments (b=–3.27, SE = 1.56, t=–2.09, p=0.0368). This effect was strongest in the Interoceptive experiment, where Faster feedback led to a markedly greater increase in pain intensity. The effect of Slower vs. No feedback differed significantly between experiments, with a stronger difference in the Interoceptive experiment (b=–5.45, SE = 1.56, t=–3.501, p=0.0005). Likewise, the Congruent vs. No feedback contrast was more pronounced in the Interoceptive experiment (b=–3.78, SE = 1.58, t=–2.390, p=0.0170). No other between-experiment differences were significant.

###### 1.3.1.2 Additional unpredicted effects

A Type III ANOVA revealed a significant *main effect of Trial* on Numeric pain scale of intensity ratings, F(1, 61.94)=17.56, p<0.001, suggesting that pain ratings varied throughout the experimental session. However, the fixed-effect estimate of the Trial coefficient did not reach statistical significance (b=5.30, SE = 3.67, t(210.37)=1.45, p=0.15), indicating a non-significant trend toward increasing intensity ratings over time. These interactions are reported in detail below.

There was also a significant main effect of *Stimulus Intensity (StimInt)* on pain ratings, F(1, 62.32)=386.62, p<0.001. Specifically, pain ratings increased significantly as the intensity of the stimulus increased, with a coefficient estimate of 37.54 (SE = 2.90), t=12.94, p<0.001. This indicates a strong and significant positive relationship between stimulus intensity and reported pain levels.

Additionally, the effect of trial on pain ratings varied as a function of the actual intensity of the nociceptive stimulation, as indicated by a significant *Stimulus Intensity × Trial interaction*, *F*(1, 1249.76)=14.07, p<0.001.

To further investigate this interaction, estimated marginal trends of the Numeric pain scale of intensity ratings across trials were computed at three levels of stimulus intensity: low (–0.5), medium (0), and high (+0.5). Pain ratings increased progressively over trials at all intensity levels, but the rate of increase became steeper as stimulus intensity increased. At low intensity, the slope was positive but only marginally significant (*b*=3.77, SE = 2.26, 95% CI [–0.72, 8.25], *t*(109.3)=1.67, p=0.099). A significant positive trend emerged at medium intensity (*b*=8.26, SE = 1.97, 95% CI [4.32, 12.20], *t*(63.8)=4.19, p=0.0001) and was even more pronounced at high intensity (*b*=12.75, SE = 2.35, 95% CI [8.10, 17.40], *t*(126.6)=5.43, p<0.0001).

Pairwise comparisons between slopes (FDR-corrected) confirmed that the increase in Numeric pain scale of intensity ratings over trials was significantly steeper at higher levels of stimulus intensity. Specifically, the slope at low intensity was significantly lower than both the medium (*estimate* = –4.49, SE = 1.20, *t*(1251) = –3.75, p=0.0002) and high-intensity levels (*estimate* = –8.99, SE = 2.40, *t*(1251) = –3.75, p=0.0002). Moreover, the slope at medium intensity was significantly lower than at high intensity (*estimate* = –4.49, SE = 1.20, *t*(1251) = –3.75, p=0.0002).

The *Feedback × Stimulus Intensity interaction* was also significant, *F*(3, 1237.89)=5.27, p=0.001, indicating that the effect of stimulus intensity on pain ratings varied across feedback conditions. To further unpack this interaction, estimated marginal means (EMMs) of Numeric pain scale of intensity ratings were computed at the three levels of stimulus intensity: low (–0.5), medium (0), and high (+0.5).

At *low stimulus intensity* (–0.5), the Faster feedback condition elicited the highest pain ratings (M=43.5, SE = 2.09), followed by Slower (M=42.5, SE = 2.09), Congruent (M=39.0, SE = 2.11), and No Feedback (M=34.6, SE = 2.09). Ratings in the Faster condition were significantly higher than those in the Congruent condition (estimate = –4.42, SE = 1.50, t(1309) = –2.94, p=0.0050), and also significantly higher than those in the No Feedback condition (estimate = 8.85, SE = 1.48, t(1308)=5.99, p<0.0001). Likewise, the Slower condition elicited significantly higher ratings than Congruent, t(1306)=–2.284, p=0.0271, and No Feedback, t(1307)=5.346, p<0.0001. Ratings in the No Feedback condition were significantly lower than in all other feedback conditions (all ps <0.0006).

At *medium intensity* (0), Faster again produced the highest ratings (M=60.5, SE = 1.93), followed by Slower (M=57.3, SE = 1.92), Congruent (M=57.3, SE = 1.93), and No Feedback (M=50.5, SE = 1.92). Post hoc comparisons confirmed that Faster was significantly higher than both Congruent (estimate = –3.25, SE = 0.95, t(1301) = –3.44, p=0.0007) and Slower (estimate = –3.20, SE = 0.93, t(1300) = –3.45, p=0.0007), while all three active feedback conditions yielded significantly higher ratings than No Feedback (all ps <0.0001).

At *high intensity* (+0.5), Faster again elicited the highest ratings (M=77.6, SE = 2.32), followed by Congruent (M=75.6, SE = 2.34), Slower (M=72.2, SE = 2.31), and No Feedback (M=66.3, SE = 2.31). The No Feedback condition was again significantly lower than all others (all ps <0.0002), and Faster was significantly higher than Slower (estimate = –5.40, SE = 1.47, t(1306) = –3.67, p=0.0004). A significant difference was also observed between Congruent and Slower (estimate = 3.33, SE = 1.49, t(1307)=2.23, p=0.0311), while the difference between Congruent and Faster was not significant (p=0.1706).

Numeric pain scale of intensity ratings increased as a function of stimulus intensity in all feedback conditions. However, the rate of increase (slope) differed depending on the feedback type. The steepest increase was observed in the Congruent condition (b=36.5, SE = 2.20, t(197) = 16.59, p<0.0001), followed by Faster (b=34.2, SE = 2.17, t(186) = 15.77, p<0.0001), No Feedback (b=31.7, SE = 2.15, t(178) = 14.75, p<0.0001), and Slower (b=29.8, SE = 2.15, t(178) = 13.86, p<0.0001). Pairwise comparisons between slopes revealed that the increase in pain ratings was significantly steeper in the Congruent condition compared to Slower (estimate = 6.74, SE = 2.33, t(1312)=2.90, p=0.0229). Differences between other slopes did not reach statistical significance (all ps >0.11).

The interaction between *Feedback x Trial* was significant, *F*(3, 1238.72)=20.77, p<0.0001, indicating that the trajectory of pain ratings across trials varied depending on the type of feedback received.

To further unpack this interaction, the estimated slopes of Trial were computed for each feedback condition. Pain ratings increased significantly across trials in both the Congruent (*b*=6.58, SE = 2.61, 95% CI [1.42, 11.73], *t*(190) = 2.52, p=0.0127) and Slower feedback conditions (*b*=6.85, SE = 2.42, 95% CI [2.06, 11.63], *t*(142) = 2.83, p=0.0053), indicating a moderate but consistent increase in perceived pain over time. In contrast, no significant change across trials was observed in the Faster condition (*b* = –1.51, SE = 2.58, 95% CI [–6.59, 3.57], *t*(181) = –0.59, p=0.558). The steepest and most robust increase in pain ratings over time was found in the No Feedback condition (*b*=21.27, SE = 2.77, 95% CI [15.80, 26.73], *t*(235) = 7.67, p<0.0001).

Post hoc pairwise comparisons of slopes (FDR-corrected) confirmed that the rate of pain increase over trials was significantly greater in the No Feedback condition compared to all other feedback types (all ps <0.0001). Specifically, the slope in the No Feedback condition was significantly higher than that of the Congruent (*estimate* = –14.69, SE = 2.94, *t*(1244) = –4.99, p<0.0001), Slower (*estimate* = –14.42, SE = 2.76, *t*(1241) = –5.22, p<0.0001), and Faster conditions (*estimate* = –22.78, SE = 2.91, *t*(1241) = –7.83, p<0.0001). Additionally, the slope in the Faster condition was significantly lower than that of both the Congruent (estimate = 8.090, SE = 2.76, *t*(1240)=2.931, p=0.0041) and Slower conditions (estimate = 8.359, SE = 2.57, *t*(1236)=3.251, p = 0.0018), while no significant difference emerged between the Congruent and Slower conditions (p=0.918).

To further explore how the effect of feedback on pain ratings evolved over time, estimated marginal means of Numeric pain scale of intensity ratings were computed at three levels of the standardized Trial variable (–0.5, 0, +0.5), representing earlier, middle, and later stages of the experimental session, respectively. This analysis allowed us to probe whether the differences in pain ratings across feedback conditions were stable or varied as the experiment progressed.

At *early trials* (Trial = –0.5), pain ratings differed markedly across feedback conditions. Ratings were significantly higher in the Faster condition (M=61.5, SE = 2.35) compared to both the Congruent (M=54.4, SE = 2.27; estimate = –7.10, SE = 1.54, t(1235) = –4.62, p<0.0001) and Slower conditions (M=54.4, SE = 2.29; estimate = –7.11, SE = 1.57, t(1233) = –4.53, p<0.0001). In contrast, the No Feedback condition elicited the lowest pain ratings (M=40.4, SE = 2.40), significantly lower than all other conditions (all ps <0.0001), with the largest difference observed when compared to the Faster condition (estimate = –21.03, SE = 1.72, t(1239) = –12.23, p<0.0001).

At *middle trials* (Trial = 0), the pattern of feedback differences remained largely consistent. Pain ratings were again significantly higher in the Faster condition (M=60.7, SE = 1.95) than in the Congruent (M=57.7, SE = 1.96; estimate = –3.06, SE = 0.80, t(1231) = –3.84, p=0.0002) and Slower conditions (M=57.8, SE = 1.95; estimate = –2.93, SE = 0.78, t(1231) = –3.75, p=0.0002). The No Feedback condition continued to yield significantly lower ratings (M=51.1, SE = 1.95) compared to all other feedback conditions (all ps <0.0001).

At *later trials* (Trial = 0.5), the feedback-related differences in pain ratings disappeared. No significant differences emerged among any of the feedback conditions (all ps >0.87), and pain ratings converged across all feedback types: Congruent (M=60.9, SE = 2.44), Slower (M=61.2, SE = 2.30), Faster (M=60.0, SE = 2.33), and No Feedback (M=61.7, SE = 2.39). This pattern suggests that while feedback modulated pain ratings in the earlier and middle stages of the experiment, these effects dissipated over time, resulting in equivalent pain ratings by the end of the session.

### 2. Interoceptive experiment (within-subjects model)

#### 2.1 Heart rate

To examine variations in heart rate (HR) within the interoceptive experiment, we applied general linear mixed-effects modeling (GLMM), the model was fitted using R (lme4 package; Bates et al., 2015) and was specified as follows:

$$\displaystyle HR\sim Feedback\times Trail+(Trai\mid Subject)$$

where Feedback and Trial were included as fixed effects, and a random slope for Trial at the subject level (Trial | Subject) to account for individual variability in HR trajectories over time. Model comparison indicated that the inclusion of a random slope significantly improved model fit (χ²=13.493, p=0.001).

##### 2.1.1 Results

###### 2.1.1.1 Effects of interest

The Type III ANOVA revealed a significant *main effect of Feedback*, F(3, 707.58)=60.70, p<0.0001, indicating that HR levels varied depending on the type of feedback received. Compared to the Congruent condition (M=74.2, SE = 1.37), HR was significantly lower in the Faster feedback condition (M=73.1, SE = 1.37; estimate = –1.05, SE = 0.25, t(708) = - 4.28, p<0.0001), and significantly higher in the No Feedback condition (M=76.2, SE = 1.37; estimate = 2.03, SE = 0.24, t(707) = 8.32, p<0.0001). In contrast, the Slower feedback condition did not significantly differ from the Congruent (M=73.8, SE = 1.37, estimate = –0.37, SE = 0.24, t(705.6) = –1.53, p=0.1277). *Post hoc comparisons* further clarified these effects. Relative to all feedback conditions, the No feedback elicited a significantly increased heart rate (all ps <0.0001). Additionally, heart rate decreased significantly under Faster feedback relative to the Slower (estimate = 0.694, SE = 0.240, t(708) = 2.898, p=0.0046) and Congruent feedback (estimate = 1.058, SE = 0.247, t(708) = 4.284, p<0.0001). The Slower feedback condition did not differ significantly from the Congruent condition (p=0.14).

###### 2.1.1.2 Additional unpredicted effects

A significant *main effect of Trial* also emerged, F(1, 34.95) = 8.23, p=0.0069, indicating that HR increased throughout the experiment (b=2.66, SE = 0.74, t(164.7)=3.58, p=0.0004).

The interaction between *Feedback × Trial* was significant, F(3, 711.19) = 6.16, p<0.001, indicating that the trajectory of heart rate (HR) across trials varied depending on the type of feedback received (see Appendix 1—figure 1).

To further unpack this interaction, the estimated slopes of Trial were computed for each feedback condition. HR increased significantly across trials in both the Congruent (b=2.67, SE = 0.74, 95% CI [1.20, 4.13], t(164) = 3.58, p=0.0005) and Faster feedback conditions (b=2.66, SE = 0.68, 95% CI [1.31, 4.00], t(119) = 3.91, p=0.0002), indicating a consistent rise in autonomic arousal over time. In contrast, the Slower condition did not show a significant change across trials (b=0.68, SE = 0.65, 95% CI [–0.61, 1.98], t(104) = 1.04, p=0.2993), while the No Feedback condition showed a flat-to-decreasing pattern that was also non-significant (b = –0.34, SE = 0.75, 95% CI [–1.82, 1.15], t(169) = –0.44, p=0.6574).

Post hoc pairwise comparisons of slopes (FDR-corrected) confirmed that the rate of HR increase over trials was significantly higher in the Congruent condition compared to both Slower (estimate = 1.98, SE = 0.81, t(707) = 2.44, p=0.0227) and No Feedback conditions (estimate = 3.00, SE = 0.90, t(715) = 3.34, p=0.0026). The slope in the Faster condition was also significantly greater than that of Slower (estimate = 1.98, SE = 0.76, t(707) = 2.61, p=0.0188) and No Feedback (estimate = 2.99, SE = 0.85, t(714) = 3.54, p=0.0026). No significant difference emerged between Congruent and Faster feedback (p=0.9928), nor between Slower and No Feedback (p=0.2611).

To further explore how the effect of feedback on heart rate (HR) evolved over time, estimated marginal means of HR were computed at three levels of the standardized Trial variable (–0.5, 0, +0.5), representing early, middle, and late stages of the experimental session. This analysis allowed us to assess whether differences in HR across feedback conditions were stable or changed as the session progressed.

At *early trials* (Trial = –0.5), HR was significantly higher in the No Feedback condition (M=76.4, SE = 1.33) compared to all other feedback conditions. Specifically, HR was significantly higher than in the Faster (M=71.8, SE = 1.33; estimate = –4.57, SE = 0.52, t(713) = –8.86, p<0.0001), Slower (M=73.5, SE = 1.32; estimate = –2.90, SE = 0.48, t(713) = –6.08, p<0.0001), and Congruent conditions (M=72.8, SE = 1.32; estimate = –3.52, SE = 0.47, t(715) = –7.43, p<0.0001). Additionally, the Faster condition elicited significantly lower HR than both the Slower (estimate = 1.68, SE = 0.48, t(705) = 3.52, p=0.0007) and Congruent conditions (estimate = 1.05, SE = 0.47, t(707) = 2.23, p=0.0315), while no significant difference emerged between Congruent and Slower feedback (p=0.1472).

At *middle trials* (Trial = 0), the same pattern of differences was observed. HR in the No Feedback condition (M=76.2, SE = 1.37) remained significantly higher than in all other conditions (all ps <0.0001). The Faster condition (M=73.1, SE = 1.37) was again significantly lower than Slower (M=73.8, SE = 1.37; estimate = –0.69, SE = 0.24, t(708) = –2.89, p=0.0048) and Congruent (M=74.2, SE = 1.37; estimate = –1.06, SE = 0.25, t(708) = –4.28, p<0.0001). Congruent and Slower did not differ significantly (p=0.1277).

By *later trials* (Trial = 0.5), HR values across conditions began to converge. Although No Feedback (M=76.0, SE = 1.50) was still numerically higher, it no longer differed significantly from Congruent (M=75.5, SE = 1.51; estimate = –0.52, SE = 0.55, t(712) = –0.95, p=0.4092). However, Faster (M=74.4, SE = 1.48) and Slower (M=74.1, SE = 1.49) still showed significantly lower HR compared to No Feedback (Faster vs. No Feedback: estimate = –1.58, SE = 0.46, t(712) = –3.46, p=0.0017; Slower vs. No Feedback: estimate = –1.88, SE = 0.47, t(711) = –3.97, p=0.0005). The Congruent condition was significantly higher than Slower (estimate = 1.36, SE = 0.51, t(706) = 2.65, p=0.0163) and marginally higher than Faster (estimate = 1.06, SE = 0.50, t(712) = 2.13, p=0.0507).

Taken together, these results indicate that the Faster feedback condition consistently elicited lower heart rate (HR) compared to the other feedback types, particularly during the early and middle phases of the experimental session, and this effect tended to vanish over later trials.

#### 2.2 Likert pain unpleasantness

To examine variations in pain unpleasantness ratings within the interoceptive experiment, we applied general linear mixed-effects modeling (GLMM), the model was fitted using R (lme4 package; Bates et al., 2015) and was specified as follows:

$$\displaystyle \begin{array}{ll}\text{LIKERT PAIN UNPLEASANTNESS RATINGS} \sim \\ {\rm Feedback} \times {\rm StimInt} \times {\rm Trial} + (\rm StimInt +Trial | Subject)\end{array}$$

where Feedback, StimInt, and Trial were included as fixed effects. A random slope for StimInt and Trial was included at the subject level (StimInt +Trial | Subject) to account for individual differences in pain perception across stimulus intensities and over time. Model comparison confirmed that including these random slopes significantly improved model fit (χ²=13.493 p=0.001).

##### 2.2.1 Results

###### 2.2.1.1 Effects of interest

The Type III ANOVA revealed a significant *main effect of Feedback*, F(3, 669.05)=19.60, p<0.0001, indicating that pain unpleasantness ratings varied as a function of the type of feedback received. Compared to the Congruent condition (M=3.02, SE = 0.11), unpleasantness ratings were significantly higher in the Faster feedback condition (M=3.17, SE = 0.11; estimate = –0.14, SE = 0.05, t(670) = –2.92, p=0.0044), and significantly lower in the No Feedback condition (M=2.80, SE = 0.11; estimate = 0.22, SE = 0.05, t(669) = 4.58, p<0.0001). In contrast, the Slower feedback condition (M=2.97, SE = 0.11) did not significantly differ from the Congruent condition (estimate = –0.05, SE = 0.05, t(668) = –0.99, p=0.3112).

Post hoc comparisons further clarified these effects. Ratings in the No Feedback condition were significantly lower than in all other feedback conditions (all ps <0.0005). Additionally, unpleasantness ratings were significantly higher in the Faster feedback condition compared to both the Slower (estimate = 0.19, SE = 0.05, t(669) = 4.02, p=0.0001) and Congruent conditions (p=0.0044). The Slower condition did not significantly differ from Congruent (p=0.3112), but was associated with significantly higher unpleasantness than the No Feedback condition (estimate = 0.17, SE = 0.05, t(669) = 3.67, p=0.0004).

###### 2.2.1.1 Additional unpredicted effects

A robust *main effect of Stimulus Intensity* also emerged, F(1, 34.04) = 169.12, p<0.0001, indicating that unpleasantness ratings increased significantly with higher stimulus intensities. The fixed-effect estimate confirmed a strong positive relationship (b=1.52, SE = 0.12, t(89.6)=12.18, p<0.0001), suggesting that stronger pain stimulation was reliably associated with higher unpleasantness evaluations.

A Type III ANOVA revealed a significant *main effect of Trial* on pain unpleasantness ratings, F(1, 34.46)=11.30, p=0.0019, suggesting that unpleasantness ratings varied throughout the experimental session. However, the fixed-effect estimate of the Trial coefficient did not reach statistical significance (b=0.25, SE = 0.16, t(140.90)=1.57, p=0.118), indicating a non-significant trend toward increasing unpleasantness ratings over time. These interactions are reported in detail below.

The interaction between *Stimulus Intensity × Feedback* was significant, F(3, 672.32)=3.90, p=0.0088, indicating that the effect of stimulus intensity on pain unpleasantness ratings varied depending on the type of feedback received (see Appendix 1—figure 2). To further examine this interaction, estimated marginal means (EMMs) of unpleasantness ratings were computed at three levels of stimulus intensity: low (–0.5), medium (0), and high (+0.5).

The interaction between Stimulus Intensity and Feedback was significant, *F*(3, 672.32)=3.90, p=0.0088, indicating that the effect of stimulus intensity on pain unpleasantness ratings varied as a function of the type of feedback received. To explore this interaction, estimated marginal means (EMMs) of unpleasantness were examined at three levels of stimulus intensity: low (–0.5), medium (0), and high (+0.5).

At low stimulus intensity (–0.5), the highest unpleasantness ratings were observed under the Faster feedback condition (*M*=2.58, SE = 0.10), followed by Slower (*M*=2.36, SE = 0.10), Congruent (*M*=2.26, SE = 0.11), and No Feedback (*M*=2.23, SE = 0.10). Post hoc comparisons showed that Faster feedback elicited significantly higher ratings than both Congruent (estimate = –0.32, SE = 0.08, *t*(675) = –3.97, p=0.0002) and No Feedback (estimate = 0.35, SE = 0.08, *t*(673) = 4.55, p<0.0001). Ratings under Faster were also significantly higher than Slower (estimate = 0.22, SE = 0.08, *t*(675) = –2.84, p=0.0094). No other contrasts reached significance.

At medium intensity (0), the Faster condition again yielded the highest ratings (*M*=3.17, SE = 0.11), followed by Congruent (*M*=3.02, SE = 0.11), Slower (*M*=2.97, SE = 0.11), and No Feedback (*M*=2.80, SE = 0.11). Ratings under Faster were significantly higher than Congruent (estimate = –0.14, SE = 0.05, *t*(670) = –2.93, p=0.0042), Slower (estimate = –0.19, SE = 0.05, *t*(669) = –4.02, p=0.0001), and No Feedback (estimate = 0.37, SE = 0.05, *t*(671) = 7.61, p<0.0001). Both Congruent and Slower feedback also produced significantly higher ratings than No Feedback (all ps <0.001).

At high intensity (+0.5), unpleasantness ratings peaked under Congruent feedback (*M*=3.78, SE = 0.14), followed by Faster (*M*=3.76, SE = 0.14), Slower (*M*=3.59, SE = 0.14), and No Feedback (*M*=3.37, SE = 0.14). No Feedback elicited significantly lower ratings than all other conditions (all ps <0.01). In addition, both Congruent (estimate = 0.20, SE = 0.08, *t*(671) = 2.52, p=0.0179) and Faster (estimate = 0.17, SE = 0.08, *t*(671) = 2.21, p=0.0326) conditions elicited significantly higher ratings than Slower. No significant difference was found between Congruent and Faster (estimate = 0.03, SE = 0.08, *t*(670) = 0.33, p=0.7415).

Unpleasantness ratings increased significantly with stimulus intensity across all feedback conditions. However, the rate of increase (slope) varied by feedback type. The steepest increase was observed in the Congruent condition (b=1.52, SE = 0.13, t(88.6)=12.19, p<0.0001), followed by Slower (b=1.23, SE = 0.12, t(76.6)=10.23, p<0.0001), Faster (b=1.18, SE = 0.12, t(85.0)=9.56, p<0.0001), and No Feedback (b=1.14, SE = 0.12, t(77.5)=9.51, p<0.0001). Pairwise comparisons of slopes (FDR-corrected) confirmed that the slope in the Congruent condition was significantly steeper than in Slower (estimate = 0.29, SE = 0.12, t(672) = 2.43, p=0.0305), Faster (estimate = 0.34, SE = 0.12, t(673) = 2.76, p=0.0177), and No Feedback (estimate = 0.38, SE = 0.12, t(672) = 3.12, p=0.0114). No other pairwise slope differences reached significance (all ps >0.70).

The interaction between *Feedback × Trial* was significant, F(3, 675.33)=3.44, p=0.0165, indicating that the trajectory of unpleasantness ratings across trials varied depending on the type of feedback received (See Appendix 1—figure 3). To further unpack this interaction, the estimated slopes of Trial were computed for each feedback condition. Unpleasantness ratings increased significantly over time in the Slower feedback condition (b=0.35, SE = 0.14, 95% CI [0.07, 0.62], t(91) = 2.50, p=0.0143), and even more robustly in the No Feedback condition (b=0.69, SE = 0.16, 95% CI [0.38, 1.00], t(134) = 4.43, p<0.0001). In contrast, the increase observed in the Congruent (b=0.25, SE = 0.16, 95% CI [–0.06, 0.56], t(142) = 1.58, p=0.1174) and Faster conditions (b=0.18, SE = 0.14, 95% CI [–0.10, 0.46], t(101) = 1.26, p=0.2122) did not reach statistical significance.

Post hoc pairwise comparisons of slopes (FDR-corrected) confirmed that the rate of increase in unpleasantness over trials was significantly higher in the No Feedback condition compared to both Congruent (estimate = –0.44, SE = 0.18, t(679) = –2.46, p=0.0430) and Faster feedback (estimate = –0.51, SE = 0.17, t(677) = –3.05, p=0.0144). The difference between No Feedback and Slower was marginally significant (estimate = –0.34, SE = 0.16, t(676) = –2.10, p=0.0730), while no significant differences were found among the active feedback conditions (all ps >0.40).

To further explore how the effect of feedback on unpleasantness evolved over time, estimated marginal means were computed at three levels of the standardized Trial variable (–0.5, 0, +0.5), representing early, middle, and late stages of the session, respectively. This analysis allowed us to assess whether the differences between feedback conditions were stable or changed over time.

At *early trials* (Trial = –0.5), unpleasantness ratings were highest in the Faster condition (M=3.08, SE = 0.13), followed by Congruent (M=2.90, SE = 0.13), Slower (M=2.80, SE = 0.13), and No Feedback (M=2.46, SE = 0.13). Ratings in the No Feedback condition were significantly lower than in all other feedback conditions (all ps <0.0007). Moreover, Faster feedback elicited significantly higher ratings than Slower (estimate = –0.28, SE = 0.10, t(671) = –2.90, p=0.0058) and marginally higher ratings than Congruent (estimate = –0.18, SE = 0.09, t(671) = –1.88, p=0.0733).

At *middle trials* (Trial = 0), the pattern remained consistent. Unpleasantness ratings were again highest in the Faster condition (M=3.17, SE = 0.11), followed by Congruent (M=3.02, SE = 0.11), Slower (M=2.98, SE = 0.11), and No Feedback (M=2.80, SE = 0.11). Ratings in the No Feedback condition were significantly lower than in all other feedback types (all ps <0.0005), and Faster was significantly higher than both Congruent (estimate = –0.14, SE = 0.05, t(670) = –2.91, p=0.0045) and Slower (estimate = –0.19, SE = 0.05, t(669) = –4.01, p=0.0001).

By *late trials* (Trial = 0.5), feedback-related differences in unpleasantness ratings had largely dissipated, with no statistically significant differences among feedback conditions (all ps >0.55). Nonetheless, the Faster condition continued to elicit the highest unpleasantness ratings (M=3.26, SE = 0.12), slightly above Congruent (M=3.15, SE = 0.14), Slower (M=3.15, SE = 0.12), and No Feedback (M=3.15, SE = 0.13). Although these differences were no longer significant, the numerical pattern suggests a persistent trend toward heightened unpleasantness in the presence of accelerated feedback, even in the final stage of the session. This convergence indicates that, over time, the modulatory effect of feedback on unpleasantness attenuated.

Additionally, the effect of Trial on pain unpleasantness ratings varied as a function of the actual intensity of the nociceptive stimulation, as indicated by a significant *Stimulus Intensity × Trial* interaction, *F*(1, 680.64)=5.44, p=0.0199. To further investigate this interaction, estimated marginal trends of unpleasantness ratings across trials were computed at three levels of stimulus intensity: low (–0.5), medium (0), and high (+0.5). Unpleasantness ratings increased progressively over trials at all intensity levels, but the rate of increase was steeper as stimulus intensity increased.

At low intensity, the slope was positive but did not reach statistical significance (*b*=0.20, SE = 0.13, 95% CI [–0.06, 0.45], *t*(68.8)=1.50, p=0.1377). A significant upward trend emerged at medium intensity (*b*=0.37, SE = 0.11, 95% CI [0.14, 0.59], *t*(34.6)=3.36, p=0.0019), which became even more pronounced at high intensity (*b*=0.54, SE = 0.13, 95% CI [0.27, 0.80], *t*(74.7)=4.04, p=0.0001).

Pairwise comparisons between slopes (FDR-corrected) confirmed that the increase in unpleasantness ratings over trials was significantly steeper at higher stimulus intensities. Specifically, the slope at low intensity was significantly shallower than both medium (estimate = –0.17, SE = 0.073, *t*(681) = –2.33, p=0.0201) and high intensity (estimate = –0.34, SE = 0.15, *t*(681) = –2.33, p=0.0201). Moreover, the slope at medium intensity was also significantly lower than at high intensity (estimate = –0.17, SE = 0.073, *t*(681) = –2.33, p=0.0201). These results suggest that the impact of repeated nociceptive stimulation on perceived unpleasantness intensifies progressively with the strength of the stimulation.

#### 2.3 Numeric Pain Scale of intensity ratings

To examine variations in pain intensity ratings within the interoceptive experiment, we applied general linear mixed-effects modeling (GLMM), the model was fitted using R (lme4 package; Bates et al., 2015) and was specified as follows:

$$\displaystyle \begin{array}{ll}\text{NUMERIC PAIN SCALE OF INTENSITY RATINGS }\sim \\ {\rm Feedback} \times {\rm StimInt} \times {\rm Trial} + (\rm StimInt +Trial \mid Subject)\end{array}$$

where Feedback, StimInt and Trial were included as fixed effects. A random slope for StimInt and Trial was included at the subject level (StimInt +Trial | Subject) to account for individual differences in pain perception across stimulus intensities and over time. Model comparison confirmed that including these random slopes significantly improved model fit (χ²=83.437, p<0.0001).

##### 2.3.1 Results

###### 2.3.1.1 Effects of interest

The results of the Type III ANOVA with Satterthwaite’s approximation revealed a significant *main effect of Feedback*, F(3, 663.89)=22.46, p<0.001, indicating that Numeric pain scale of intensity ratings varied across feedback conditions. Compared to the reference condition (i.e. Congruent feedback), Numeric pain scale of intensity ratings were significantly higher in the Faster feedback condition (b=3.69, SE = 1.08, t(664.80)=3.40, p=0.0008), and significantly lower in the No Feedback condition (b = –4.90, SE = 1.07, t(663.18) = –4.59, p<0.0001). The Slower feedback condition did not differ significantly from the Congruent condition (b = –0.79, SE = 1.07, t(663.03) = –0.74, p=0.4623).

Post hoc comparisons further clarified these effects. Numeric pain scale of intensity ratings were significantly higher in the Faster feedback condition compared to Congruent (estimate = –3.691, SE = 1.08, *t*(665)=−3.406, p=0.0008), Slower (estimate = –4.522, SE = 1.06, *t*(665)=−4.285, p<0.0001), and No Feedback (estimate = 8.711, SE = 1.05, *t*(665)=8.260, p<0.0001). The Slower condition did not differ significantly from the Congruent condition (p=0.4350). Moreover, No Feedback yielded significantly lower Numeric pain scale of intensity ratings compared to all other feedback conditions (all ps <0.0001).

###### 2.3.1.2 Additional unpredicted effects

A Type III ANOVA revealed a significant *main effect of Trial* on Numeric pain scale of intensity ratings, F(1, 32.90)=9.01, p=0.0051, suggesting that pain ratings varied throughout the experimental session. However, the fixed-effect estimate of the Trial coefficient from the mixed model indicated a positive but non-significant trend (b=6.05, SE = 3.73, t(103.82)=1.62, p=0.108), suggesting that pain intensity ratings tended to increase over time, although this trend was not statistically reliable when averaged across all other factors in the model. This apparent discrepancy likely reflects the fact that Trial was involved in several higher-order interactions, particularly with Stimulus Intensity and Feedback, that modulated its effect on pain ratings. These interactions are reported in detail below.

Critically, there was also a robust *main effect of Stimulus Intensity*, F(1, 34.22)=204.11, p<0.001, indicating that pain ratings increased as a function of the objective intensity of nociceptive stimulation. The fixed-effect estimate showed a strong positive association (b=37.12, SE = 2.82, t(83.01)=13.14, p<0.001), consistent with the expected relationship between stimulus intensity and perceived pain.

The *Feedback × Stimulus Intensity* interaction was also significant, F(3, 667.07)=3.4631, p=0.016, indicating that the effect of stimulus intensity on pain ratings varied across feedback conditions (See Appendix 1—figure 4). To further unpack this interaction, estimated marginal means (EMMs) of Numeric pain scale of intensity ratings were computed at the three levels of stimulus intensity: low (–0.5), medium (0), and high (+0.5).

At *low stimulus intensity* (–0.5), the Faster feedback condition elicited the highest pain ratings (M=43.4, SE = 2.76), followed by Slower (M=39.6, SE = 2.74), Congruent (M=37.2, SE = 2.78), and No Feedback (M=36.3, SE = 2.74).

Ratings in the Faster condition were significantly higher than those in the Congruent (estimate = –6.260, SE = 1.74, t(668) = –3.589, p=0.0011), Slower (estimate = –3.872, SE = 1.69, t(671) = –2.289, p=0.0448), and No Feedback (estimate = 7.185, SE = 1.68, t(668) = 4.274, p=0.0001).

At *medium intensity* (0), Faster again produced the highest ratings (M=59.4, SE = 2.76), followed by Congruent (M=55.7, SE = 2.76), Slower (M=54.9, SE = 2.75), and No Feedback (M=50.7, SE = 2.75). Post hoc comparisons confirmed that Faster was significantly higher than both Congruent (estimate = –3.721, SE = 1.08, t(665) = –3.432, p=0.0008) and Slower (estimate = –4.515, SE = 1.06, t(665) = –4.277, p<0.0001), while all three active feedback conditions yielded significantly higher ratings than No Feedback (all ps <0.0002).

At *high intensity* (+0.5), Faster again elicited the highest ratings (M=75.4, SE = 3.38), followed by Congruent (M=74.2, SE = 3.39), Slower (M=70.3, SE = 3.37), and No Feedback (M=65.2, SE = 3.37). The No Feedback condition was again significantly lower than all others (all ps <0.0035), and Faster was significantly higher than Slower (estimate = –5.157, SE = 1.67, t(665) = –3.091, p=0.0032). A significant difference was also observed between Congruent and Slower (estimate = 3.976, SE = 1.68, t(666) = 2.362, p=0.0221), while the difference between Congruent and Faster was not significant (p=0.4904).

Numeric pain scale of intensity ratings increased as a function of stimulus intensity in all feedback conditions. However, the rate of increase (slope) differed depending on the feedback type. The steepest increase was observed in the Congruent condition (b=37.1, SE = 2.82, t(81) = 13.161, p<0.0001), followed by Faster (b=32.0, SE = 2.78, t(77.7)=11.480, p<0.0001), Slower (b=30.7, SE = 2.74, t(72.5)=11.212, p<0.0001), and No Feedback (b=29.0, SE = 2.73, t(71.7)=10.612, p<0.0001). Pairwise comparisons between slopes revealed that the increase in pain ratings was significantly steeper in the Congruent condition compared to Slower (estimate = 6.36, SE = 2.64, t(668) = 2.411, p=0.048) and No feedback (estimate = 8.10, SE = 2.64, t(668) = 3.07, p=0.0133). Differences between other slopes did not reach statistical significance (all ps >0.11).

The interaction between *Feedback × Trial* was significant, *F*(3, 669.15)=9.86, p<0.001, indicating that the trajectory of Numeric pain scale of intensity ratings across trials varied depending on the type of feedback received (See Appendix 1—figure 5). To further unpack this interaction, the estimated slopes of Trial were computed for each feedback condition. Pain intensity ratings increased significantly over time in the Slower feedback condition (*b*=7.69, SE = 3.37, 95% CI [0.97, 14.41], *t*(73.3)=2.28, p=0.0255), and even more robustly in the No Feedback condition (*b*=19.61, SE = 3.68, 95% CI [12.31, 26.91], *t*(101.1)=5.33, p<0.0001).

Post hoc pairwise comparisons of slopes (FDR-corrected) confirmed that the rate of increase in pain intensity over trials was significantly higher in the No Feedback condition compared to Congruent (estimate = –13.48, SE = 3.93, *t*(673) = –3.43, p=0.0018), Slower (estimate = –11.92, SE = 3.57, *t*(670) = –3.34, p=0.0018), and Faster (estimate = –19.62, SE = 3.66, *t*(671) = –5.37, p<0.0001). Additionally, the slope in the Slower condition was significantly higher than in the Faster condition (estimate = 7.70, SE = 3.34, *t*(668) = 2.30, p=0.0323), while no significant differences were found among the active feedback conditions (all ps >0.11).

To further explore how the effect of feedback on pain intensity evolved over time, estimated marginal means were computed at three levels of the standardized Trial variable (–0.5, 0, +0.5), representing early, middle, and late stages of the session. This analysis allowed us to assess whether the differences between feedback conditions were stable or changed over time.

At *early trials* (Trial = –0.5), pain intensity ratings were highest in the Faster condition (M=59.6, SE = 3.33), followed by Congruent (M=52.9, SE = 3.23), Slower (M=51.3, SE = 3.23), and No Feedback (M=41.2, SE = 3.31). Ratings in the No Feedback condition were significantly lower than in all other feedback conditions (all ps <0.0001). Moreover, Faster feedback elicited significantly higher pain ratings than both Congruent (estimate = –6.73, SE = 2.09, t(666) = –3.23, p=0.0016) and Slower (estimate = –8.33, SE = 2.08, t(666) = –3.99, p=0.0001).

At *middle trials* (Trial = 0), the pattern remained consistent: Faster feedback again produced the highest pain ratings (M=59.6, SE = 2.76), followed by Congruent (M=56.0, SE = 2.77), Slower (M=55.1, SE = 2.75), and No Feedback (M=51.0, SE = 2.75). Ratings in the No Feedback condition were significantly lower than all others (all ps <0.0005). Additionally, Faster was significantly higher than both Congruent (estimate = –3.66, SE = 1.09, t(666) = –3.37, p=0.0010) and Slower (estimate = –4.48, SE = 1.05, t(665) = –4.26, p<0.0001).

By *late trials* (Trial = 0.5), feedback-related differences in pain ratings had largely dissipated, with no statistically significant contrasts among conditions (all ps >0.94). Nevertheless, Faster still elicited numerically higher pain ratings (M=59.6, SE = 3.17) compared to Congruent (M=59.0, SE = 3.44), Slower (M=59.0, SE = 3.23), and No Feedback (M=60.8, SE = 3.31), suggesting a potential residual effect of feedback on pain perception. However, this trend was no longer statistically significant, indicating that the modulatory influence of feedback on the Numeric Pain Scale of intensity ratings diminished over time.

### 3 Exteroceptive experiment (within-subjects model)

#### 3.1 Heart rate

To examine variations in heart rate (HR) within the exteroceptive experiment, we applied general linear mixed-effects modeling (GLMM); the model was fitted using R (lme4 package; Bates et al., 2015) and was specified as follows:

$$\displaystyle HR\sim Feedback\times Trail+(Trail\mid Subject)$$

where Feedback and Trial were included as fixed effects, and a random slope for Trial at the subject level (Trial | Subject) to account for individual variability in HR trajectories over time. Model comparison indicated that the inclusion of a random slope significantly improved model fit (χ²=11.426, p=0.0435).

##### 3.1.1 Results

###### 3.1.1.1 Effects of interest

The Type III ANOVA revealed a significant *main effect of Feedback* on heart rate (HR), F(3, 596.03)=44.43, p<0.0001, indicating that HR levels varied depending on the type of feedback received. Compared to the Congruent condition (M=76.5, SE = 1.67), HR was significantly higher in the No Feedback condition (M=78.8, SE = 1.67; estimate = 2.19, SE = 0.25, t(597.5)=8.92, p<0.0001). In contrast, neither the Slower (M=76.3, SE = 1.67; estimate = –0.29, SE = 0.24, t(594.9) = –1.21, p=0.2285) nor the Faster feedback condition (M=76.5, SE = 1.67; estimate = –0.03, SE = 0.24, t(595.1) = –0.11, p=0.9088) significantly differed from the Congruent condition.

Post hoc comparisons further clarified these effects. Relative to all other conditions, the No Feedback condition elicited significantly higher HR values (all ps <0.0001). Specifically, HR was significantly higher in the No Feedback condition compared to both the Slower (estimate = –2.50, SE = 0.24, t(598) = –10.29, p<0.0001) and Faster conditions (estimate = –2.24, SE = 0.24, t(598) = –9.22, p<0.0001).

*No significant differences were found among the active feedback conditions (all ps >0.33)*, suggesting that the HR modulation was driven specifically by the absence of feedback, rather than differences among feedback types.

No other main effects or interactions reached statistical significance (See Appendix 1—figure 6 for illustrative purposes).

#### 3.2 Likert pain unpleasantness

To examine variations in pain unpleasantness ratings within the exteroceptive experiment, we applied general linear mixed-effects modeling (GLMM); the model was fitted using R (lme4 package; Bates et al., 2015) and was specified as follows:

$$\displaystyle \begin{array}{ll}\text{LIKERT PAIN UNPLEASANTNESS RATINGS} \sim\\ {\rm Feedback} \times {\rm StimInt} \times {\rm Trial} + (\rm StimInt +Trial \mid Subject)\end{array}$$

where Feedback, StimInt, and Trial were included as fixed effects. A random slope for StimInt and Trial was included at the subject level (StimInt +Trial | Subject) to account for individual differences in pain perception across stimulus intensities and over time. Model comparison confirmed that including these random slopes significantly improved model fit (*χ²*=51.538, p<0.001).

##### 3.2.1 Results

###### 3.2.1.1 Effects of interest

The Type III ANOVA revealed a significant main effect of *Feedback* on pain unpleasantness ratings, *F*(3, 563.76)=22.12, p<0.0001, indicating that ratings varied as a function of the type of feedback received. Compared to the Congruent condition (M=3.05, SE = 0.11), unpleasantness ratings were significantly lower in the No Feedback condition (M=2.76, SE = 0.11; estimate = 0.29, SE = 0.05, *t*(565) = 6.00, p<0.0001). In contrast, neither the Slower (M=3.06, SE = 0.11; estimate = –0.01, SE = 0.05, *t*(564) = –0.20, p=0.8434) nor the Faster condition (M=3.10, SE = 0.11; estimate = –0.05, SE = 0.05, *t*(564) = –1.07, p=0.4301) differed significantly from the Congruent condition.

Post hoc comparisons further clarified these effects. Ratings in the No Feedback condition were significantly lower than in all other feedback conditions (all ps <0.0001). Specifically, unpleasantness ratings were significantly higher in the Faster condition compared to No Feedback (estimate = 0.34, SE = 0.05, *t*(564) = 7.17, p<0.0001), as well as in the Slower condition compared to No Feedback (estimate = 0.30, SE = 0.05, *t*(564) = 6.31, p<0.0001), and in the Congruent compared to the No feedback (estimate = 0.28, SE = 0.04, *t*(565) = 6.00, p<0.0001).

*No significant differences emerged between active feedback conditions (all ps >0.43)*, suggesting that the changes in pain ratings were driven specifically by the absence of feedback, rather than differences among feedback types.

###### 3.2.1.2 Additional unpredicted effects

A robust main effect of *Stimulus Intensity* also emerged, *F*(1, 28.27)=190.00, p<0.0001, indicating that unpleasantness ratings increased significantly with higher intensity stimulation. The fixed-effect estimate confirmed a strong positive association between stimulus intensity and unpleasantness ratings (b=1.54, SE = 0.12, *t*(74.01)=12.98, p<0.0001), suggesting that more intense stimuli were consistently evaluated as more unpleasant.

Finally, a significant main effect of *Trial* was observed, *F*(1, 29.63)=7.85, p=0.0089, suggesting that unpleasantness ratings varied over the course of the experimental session. However, the fixed-effect estimate of the Trial coefficient did not reach statistical significance (b=0.21, SE = 0.15, *t*(89.91)=1.40, p=0.165), indicating only a non-significant trend toward increasing unpleasantness ratings across trials. This apparent discrepancy likely reflects the presence of significant higher-order interactions with Feedback and Stimulus Intensity, which are described in detail in the following sections.

The interaction between *Stimulus Intensity × Feedback* was significant, *F*(3, 569.16)=8.21, p<0.0001, indicating that the effect of stimulus intensity on pain unpleasantness ratings varied depending on the type of feedback received (see Appendix 1—figure 7). To further examine this interaction, estimated marginal means (EMMs) of unpleasantness ratings were computed at three levels of stimulus intensity: low (–0.5), medium (0), and high (+0.5).

At *low stimulus intensity* (–0.5), Slower feedback elicited the highest unpleasantness ratings (M=2.56, SE = 0.12), followed by Faster (M=2.40, SE = 0.12), Congruent (M=2.28, SE = 0.12), and No Feedback (M=2.17, SE = 0.12). Post hoc comparisons revealed that unpleasantness ratings in the Slower condition were significantly higher than in the Congruent (estimate = –0.28, SE = 0.08, *t*(570) = –3.64, p=0.0009) and No Feedback conditions (estimate = 0.39, SE = 0.08, *t*(567) = 5.19, p<0.0001). Ratings in the Faster condition were also significantly higher than in No Feedback (estimate = 0.23, SE = 0.08, *t*(567) = 3.07, p=0.0045), but did not significantly differ from Congruent nor Slower (p>0.05).

At *medium intensity* (0), the pattern shifted slightly, with Faster feedback yielding the highest ratings (M=3.10, SE = 0.11), followed by Slower (M=3.06, SE = 0.11), Congruent (M=3.05, SE = 0.11), and No Feedback (M=2.76, SE = 0.11). All three active feedback conditions produced significantly higher unpleasantness ratings than No Feedback (all ps <0.0001). No significant differences were found among the active feedback conditions (all ps >0.42).

At *high intensity* (+0.5), unpleasantness ratings peaked under Congruent (M=3.82, SE = 0.14), followed by Faster (M=3.80, SE = 0.14), Slower (M=3.56, SE = 0.14), and No Feedback (M=3.35, SE = 0.14). Ratings in the Congruent condition were significantly higher than in Slower (estimate = 0.26, SE = 0.08, *t*(567) = 3.44, p=0.0013) and No Feedback (estimate = 0.47, SE = 0.08, *t*(567) = 6.14, p<0.0001). Similarly, Faster elicited significantly higher ratings than both Slower (estimate = –0.24, SE = 0.07, *t*(566) = –3.24, p=0.0019) and No Feedback (estimate = 0.45, SE = 0.08, *t*(567) = 5.98, p<0.0001). No difference was found between Congruent and Faster (p=0.8027).

Across all conditions, unpleasantness ratings increased significantly with higher stimulus intensity. However, the rate of increase (slope) varied depending on feedback. The steepest increase was observed in the Congruent condition (b=1.54, SE = 0.12, *t*(74.5)=12.96, p<0.0001), followed by Faster (b=1.40, SE = 0.12, *t*(70.2)=11.97, p<0.0001), No Feedback (b=1.18, SE = 0.12, *t*(70.8)=10.08, p<0.0001), and Slower (b=1.00, SE = 0.12, *t*(69.6)=8.58, p<0.0001). Pairwise comparisons of slopes confirmed that the Congruent condition showed a significantly steeper increase in unpleasantness compared to both Slower (estimate = 0.54, SE = 0.12, *t*(571) = 4.56, p<0.0001) and No Feedback (estimate = 0.36, SE = 0.12, *t*(569) = 3.02, p=0.0052). Faster also differed from Slower (estimate = 0.40, SE = 0.12, *t*(569) = 3.43, p=0.0020), while the difference between Congruent and Faster did not reach significance (p=0.2365), nor did the contrast between Faster and No Feedback (p=0.0943).

The interaction between *Feedback × Trial* was significant, *F*(3, 568.85)=11.47, p<0.0001, indicating that the trajectory of unpleasantness ratings over time varied depending on the feedback condition (see Appendix 1—figure 8). To investigate this effect, the slope of Trial was estimated within each feedback level. Unpleasantness ratings increased significantly over time in the No Feedback condition (b=0.94, SE = 0.17, 95% CI [0.61, 1.27], *t*(139.1)=5.63, p<0.0001). In contrast, the slopes for Congruent (b=0.21, SE = 0.15, 95% CI [–0.09, 0.50], *t*(90.3)=1.40, p=0.1649), Slower (b=0.23, SE = 0.14, 95% CI [–0.04, 0.51], *t*(70.8)=1.68, p=0.0973), and Faster feedback (b = –0.14, SE = 0.16, 95% CI [–0.44, 0.17], *t*(109.4) = –0.87, p=0.3843) were not statistically significant.

Post hoc comparisons of Trial slopes (FDR-corrected) confirmed that the increase in unpleasantness was significantly steeper in the No Feedback condition compared to Congruent (estimate = –0.73, SE = 0.18, *t*(571) = –4.07, p=0.0001), Slower (estimate = –0.71, SE = 0.17, *t*(570) = –4.11, p=0.0001), and Faster (estimate = –1.08, SE = 0.19, *t*(570) = –5.76, p<0.0001). Additionally, the Slower condition showed a significantly steeper slope than the Faster condition (estimate = 0.37, SE = 0.16, *t*(567) = 2.28, p=0.0343). No significant differences were found among the active feedback conditions (all ps >0.05).

To examine how feedback differences evolved over time, estimated marginal means were computed at three levels of the standardized Trial variable: early (–0.5), middle (0), and late (+0.5).

At *early trials* (Trial = –0.5), unpleasantness ratings were highest in the Faster condition (M=3.17, SE = 0.13), followed by Congruent (M=2.95, SE = 0.12), Slower (M=2.94, SE = 0.13), and No Feedback (M=2.28, SE = 0.13). Ratings in the No Feedback condition were significantly lower than in all other conditions (all ps <0.0001). Additionally, Faster feedback elicited significantly higher ratings than both Slower (p=0.0215) and Congruent (p=0.0215).

At the *midpoint* (Trial = 0), the pattern remained consistent: unpleasantness ratings were highest in the Faster condition (M=3.10, SE = 0.11), followed by Slower (M=3.06, SE = 0.11), Congruent (M=3.05, SE = 0.11), and No Feedback (M=2.75, SE = 0.11). Post hoc comparisons confirmed that the No Feedback condition was rated significantly lower than all others (all ps <0.0001), while no other differences between active feedback conditions reached significance (all ps >0.40).

By *late trials* (Trial = 0.5), unpleasantness ratings converged across conditions, with No Feedback (M=3.23, SE = 0.15), Slower (M=3.17, SE = 0.14), Congruent (M=3.15, SE = 0.15), and Faster (M=3.03, SE = 0.15) showing no statistically significant differences (all ps >0.35). Despite numerical variability, these findings indicate that feedback-related effects on unpleasantness diminished over time.

These results suggest that feedback-related differences in unpleasantness ratings were most pronounced at the beginning of the session and progressively diminished over time. Notably, the No Feedback condition was associated with a sustained and significantly steeper increase in unpleasantness ratings over trials, highlighting its distinct temporal trajectory.

Additionally, the effect of Trial on pain unpleasantness ratings varied as a function of the actual intensity of the nociceptive stimulation, as indicated by a significant *Stimulus Intensity ×Trial interaction*, F(1, 577.49)=15.61, p<0.001. To further investigate this interaction, estimated marginal trends of unpleasantness ratings across trials were computed at three levels of stimulus intensity: low (–0.5), medium (0), and high (+0.5). While unpleasantness ratings tended to increase over trials at all intensity levels, the rate of this increase became progressively steeper with higher stimulus intensities.

At low intensity, the slope was small and non-significant (b=0.02, SE = 0.13, 95% CI [–0.24, 0.28], t(53.2)=0.15, p=0.8813). A significant positive trend emerged at medium intensity (b=0.31, SE = 0.11, 95% CI [0.08, 0.54], t(29.7)=2.80, p=0.0089), which became markedly stronger at high intensity (b=0.60, SE = 0.14, 95% CI [0.33, 0.88], t(68.0)=4.38, p<0.0001).

Pairwise comparisons between slopes (FDR-corrected) confirmed that the increase in unpleasantness ratings over trials was significantly steeper at higher stimulus intensities. Specifically, the slope at low intensity was significantly shallower than both medium (estimate = –0.29, SE = 0.074, t(578) = –3.94, p=0.0001) and high intensity (estimate = –0.58, SE = 0.15, t(578) = –3.94, p=0.0001). Moreover, the slope at medium intensity was also significantly lower than at high intensity (estimate = –0.29, SE = 0.074, t(578) = –3.94, p=0.0001). These findings indicate that the cumulative effect of repeated nociceptive stimulation on perceived unpleasantness intensifies with increasing stimulation strength.

#### 3.3 Numeric Pain Scale of intensity ratings

To examine variations in pain intensity ratings within the exteroceptive experiment, we applied general linear mixed-effects modeling (GLMM), the model was fitted using R (lme4 package; Bates et al., 2015) and was specified as follows:

$$\displaystyle \begin{array}{ll}\text{NUMERIC PAIN SCALE OF INTENSITY RATINGS} \sim\\ {\rm Feedback} \times {\rm StimInt} \times{\rm Trial} + (\rm StimInt +Trial | Subject)\end{array}$$

where Feedback, StimInt, and Trial were included as fixed effects. A random slope for StimInt and Trial was included at the subject level (StimInt +Trial | Subject) to account for individual differences in pain perception across stimulus intensities and over time. Model comparison confirmed that including these random slopes significantly improved model fit (χ²=56.623, p <<0.001).

##### 3.3.1 Results

###### 3.3.1.1 Effects of interest

The results of the Type III ANOVA with Satterthwaite’s approximation revealed a significant main effect of *Feedback*, F(3, 565.43)=34.72, p<0.001, indicating that pain intensity ratings (Numeric pain scale of intensity ratings) varied across feedback conditions. Compared to the reference condition (i.e. Congruent feedback), Numeric pain scale of intensity ratings were significantly lower in the No Feedback condition (b = –8.47, SE = 1.17, t(565.85) = –7.23, p<0.0001), while the Faster feedback condition showed a marginally significant increase in ratings (b=2.23, SE = 1.17, t(564.96)=1.91, p=0.0562). The Slower feedback condition did not differ significantly from the Congruent condition (b=0.84, SE = 1.17, t(565.11)=0.72, p=0.4727).

Post hoc comparisons further clarified these effects. Numeric pain scales of intensity ratings were significantly lower in the No Feedback condition compared to all other feedback conditions (all ps <0.0001).

Specifically, intensity ratings were significantly higher in the Faster condition compared to No Feedback (estimate = 10.545, SE = 1.16, *t*(566) = 9.124, p<0.0001), as well as in the Slower condition compared to No Feedback (estimate = 9.181, SE = 1.16, *t*(566) = 7.933, p<0.0001), and in the Congruent compared to the No Feedback (estimate = 8.383, SE = 1.17, *t*(566) = 7.150, p<0.0001). *No significant differences emerged between active feedback conditions (all ps >0.10)*, suggesting that the changes in pain ratings were driven specifically by the absence of feedback, rather than differences among feedback types.

###### 3.3.1.2 Additional unpredicted effects

A significant main effect of *Trial* also emerged, F(1, 29.23)=8.86, p=0.0058, suggesting that pain intensity ratings changed throughout the experimental session. The fixed-effect estimate of the Trial coefficient indicated a significant positive trend (b=7.86, SE = 3.64, t(83.06)=2.16, p=0.0337), consistent with a general increase in pain ratings over time.

Critically, a strong main effect of *Stimulus Intensity* was observed, F(1, 28.31) = 187.17, p<0.001, indicating that pain intensity ratings increased as a function of the objective strength of nociceptive stimulation. The fixed-effect estimate confirmed a robust positive association (b=37.90, SE = 3.15, t(60.25)=12.05, p<0.001), aligning with theoretical expectations and validating the experimental manipulation of stimulus intensity.

The *Feedback × Stimulus Intensity* interaction was significant, F(3, 570.65) = 3.00, p=0.0301, indicating that the effect of stimulus intensity on pain ratings varied across feedback conditions. To further unpack this interaction, estimated marginal means (EMMs) of Numeric pain scale of intensity ratings were computed at three levels of stimulus intensity: low (–0.5), medium (0), and high (+0.5).

At *low stimulus intensity* (–0.5), the Slower condition elicited the highest pain ratings (M=44.7, SE = 3.38), followed by Faster (M=42.2, SE = 3.38), Congruent (M=40.4, SE = 3.38), and No Feedback (M=33.8, SE = 3.37). Post hoc comparisons revealed that the Slower condition yielded significantly higher ratings than Congruent (estimate = –4.24, SE = 1.87, t(570) = –2.27, p=0.0351). Moreover, all active feedback conditions (Slower, Faster, Congruent) elicited significantly higher ratings than No Feedback (all ps <0.001). No other contrasts reached significance.

At *medium intensity* (0), ratings followed a similar pattern: Faster produced the highest ratings (M=61.6, SE = 2.74), followed by Slower (M=60.2, SE = 2.74), Congruent (M=59.4, SE = 2.74), and No Feedback (M=51.1, SE = 2.74). While differences among active feedback conditions were not statistically significant (all ps >0.09), each of them led to significantly higher ratings than the No Feedback condition (all ps <0.0001).

At *high intensity* (+0.5), the highest ratings were reported in the Faster condition (M=81.0, SE = 2.91), followed by Congruent (M=78.4, SE = 2.93), Slower (M=75.8, SE = 2.91), and No Feedback (M=68.3, SE = 2.91). The No Feedback condition was significantly lower than all other feedback conditions (all ps <0.0002), and pain ratings were significantly higher in the Faster compared to the Slower condition (estimate = –5.14, SE = 1.82, t(568) = –2.83, p=0.0072). No significant difference was observed between Congruent and Faster (p=0.1682).

Numeric pain scale of intensity ratings increased with stimulus intensity in all feedback conditions. However, the rate of increase (slope) differed across feedback types. The steepest increase was observed in the Faster condition (b=38.8, SE = 3.14, t(59.4)=12.36, p<0.0001), followed closely by Congruent (b=38.0, SE = 3.15, t(60.5)=12.05, p<0.0001), No Feedback (b=34.5, SE = 3.12, t(58.2)=11.07, p<0.0001), and Slower (b=31.1, SE = 3.13, t(58.7)=9.96, p<0.0001). Pairwise comparisons between slopes revealed that the increase in pain ratings was significantly steeper in the Faster condition compared to Slower (estimate = –7.61, SE = 2.86, t(571) = –2.66, p=0.0484). All other comparisons between slopes were not statistically significant (all ps >0.05).

The interaction between *Feedback × Trial* was significant, F(3, 569.80)=12.13, p<0.001, indicating that the trajectory of Numeric pain scale of intensity ratings across trials varied depending on the type of feedback received.

To further explore this interaction, the estimated slopes of Trial were computed for each feedback condition. Pain intensity ratings increased significantly over time in the Congruent feedback condition (b=7.91, SE = 3.65, 95% CI [0.66, 15.16], t(84.5)=2.17, p=0.0329) and even more markedly in the No Feedback condition (b=23.53, SE = 4.13, 95% CI [15.36, 31.71], t(132.9)=5.69, p<0.0001). The Slower and Faster conditions did not show significant change over time (ps >0.12), although a numerically positive (b=5.39, SE = 3.45) and negative (b=–3.52, SE = 3.82) trend was observed for the Slower and Faster, respectively.

Post hoc pairwise comparisons of slopes (FDR-corrected) confirmed that the rate of increase in pain intensity was significantly higher in the No Feedback condition compared to Congruent (estimate = –15.63, SE = 4.38, t(571) = –3.57, p=0.0008), Slower (estimate = –18.14, SE = 4.23, t(571) = –4.29, p=0.0001), and Faster (estimate = –27.05, SE = 4.54, t(570) = –5.96, p<0.0001). Additionally, Congruent feedback showed a significantly steeper increase over trials compared to Faster (estimate = 11.43, SE = 4.09, t(570) = 2.79, p=0.0081), and Slower was significantly steeper than Faster (estimate = 8.91, SE = 3.92, t(568) = 2.27, p=0.0280). No other slope comparisons reached significance (all ps >0.5).

To examine how the effect of feedback on pain intensity evolved over time, estimated marginal means were computed at three levels of the standardized Trial variable (–0.5=early, 0=middle, +0.5 = late).

At *early trials* (Trial = –0.5), pain ratings were highest in the Faster condition (M=63.5, SE = 3.25), followed by Slower (M=57.6, SE = 3.19), Congruent (M=55.6, SE = 3.12), and No Feedback (M=39.3, SE = 3.40). Ratings in the No Feedback condition were significantly lower than all others (all ps <0.0001), and Faster was significantly higher than both Congruent (estimate = –7.95, SE = 2.26, t(568) = –3.52, p=0.0007) and Slower (estimate = –5.88, SE = 2.35, t(567) = –2.50, p=0.0152).

At *mid trials* (Trial = 0), the pattern was similar: Faster again elicited the highest ratings (M=61.7, SE = 2.73), followed by Slower (M=60.3, SE = 2.73), Congruent (M=59.5, SE = 2.74), and No Feedback (M=51.0, SE = 2.73). All active feedback conditions resulted in significantly higher pain ratings than No Feedback (all ps <0.0001), though differences among the active feedback types were not statistically significant (all ps >0.08).

By *late trials* (Trial = 0.5), feedback-related differences had substantially diminished. Pain ratings were comparable across conditions: Congruent (M=63.5, SE = 3.45), Slower (M=63.0, SE = 3.28), No Feedback (M=62.8, SE = 3.45), and Faster (M=60.0, SE = 3.41), with no significant pairwise differences (all ps >0.50). This indicates that while feedback had a strong modulatory effect on pain early in the session, this influence tended to fade as the session progressed.

Additionally, the effect of trial on pain ratings varied as a function of the actual intensity of the nociceptive stimulation, as indicated by a significant *Stimulus Intensity × Trial* interaction, F(1, 576.56)=14.64, p<0.001. To further investigate this interaction, estimated marginal trends of Numeric pain scale of intensity ratings across trials were computed at three levels of stimulus intensity: low (–0.5), medium (0), and high (+0.5). Pain ratings increased over trials at all intensity levels, but the rate of increase became progressively steeper with higher stimulus intensity.

At low intensity, the slope was small and not statistically significant (b=1.44, SE = 3.20, 95% CI [–4.99, 7.87], t(51.4)=0.45, p=0.6551). A significant positive trend was observed at medium intensity (b=8.28, SE = 2.78, 95% CI [2.60, 13.96], t(29.6)=2.98, p=0.0058), which became even more pronounced at high intensity (b=15.11, SE = 3.41, 95% CI [8.31, 21.92], t(65.0)=4.44, p<0.0001).

Pairwise comparisons between slopes (FDR-corrected) confirmed that the increase in Numeric pain scale of intensity ratings over trials was significantly steeper at higher levels of stimulus intensity. Specifically, the slope at low intensity was significantly lower than both medium (estimate = –6.84, SE = 1.79, t(577) = –3.82, p=0.0001) and high intensity (estimate = –13.67, SE = 3.58, t(577) = –3.82, p=0.0001). Moreover, the slope at medium intensity was also significantly lower than at high intensity (estimate = –6.84, SE = 1.79, t(577) = –3.82, p=0.0001). These findings suggest that the cumulative impact of repeated nociceptive stimulation on pain perception becomes progressively stronger with increasing stimulation intensity.
