# Supplementary material for: Exposure to false cardiac feedback alters pain perception and anticipatory cardiac frequency
Source: eLife. 2026 Jun 15;12:RP90013. doi: 10.7554/eLife.90013 (PMC13268646; doi:10.7554/eLife.90013)
Supplement: Peer review history

# Editor report: eLife Assessment

**DOI:** 10.7554/eLife.90013.3.sa0

**Authors:** José Biurrun Manresa

**Affiliations:** National Scientific and Technical Research Council (CONICET), National University of Entre Ríos (UNER) Argentina

**Keywords:** Solid, Valuable

In this **valuable** study, Parrotta et al. showed that it is possible to modulate pain perception and heart rate by providing false heart rate (HR) acoustic feedback before administering electrical cutaneous shocks. The evidence supporting the claims of the authors is rather **solid**, although what they consider an interoceptive signal is not necessarily supported as such by the results. In this regard, including a larger number of trials per participant, increasing the sample size, and adding a measure of actual pain perception after its induction would have strengthened the study. Although mechanisms and some alternative explanations for this effect remain to be addressed, the work will nonetheless be of interest to neuroscientists working on predictions and perception, health psychologists, pain researchers, and placebo researchers.

# Referee report: Reviewer #1 (Public review)

**DOI:** 10.7554/eLife.90013.3.sa1

Summary:

I read the paper by Parrotta et al with great interest. The authors are asking an interesting and important question regarding pain perception, which is derived from predictive processing accounts of brain function. They ask: If the brain indeed integrates information coming from within the body (interoceptive information) to comprise predictions about the expected incoming input and how to respond to it, could we provide false interoceptive information to modulate its predictions, and subsequently alter the perception of such input? To test this question, they use pain as the input and the sounds of heartbeats (falsified or accurate) as the interoceptive signal.

Strengths:

I found the question well-established, interesting and important, with important implications and contributions for several fields, including neuroscience of prediction-perception and pain research. The study is clearly written, the methods are generally adequate, and the results indeed support the claim that false cardiac feedback modulates both pain perception and anticipatory cardiac frequency. Importantly, the authors include a control experiment using exteroceptive auditory feedback to test whether effects are specific to heartbeat-like cues. This addition substantially strengthens interpretability.

Weaknesses:

In my view, the authors' central interpretation, namely that the effects arise because the manipulation targets interoceptive rather than exteroceptive or high-level threat-related cues, cannot be fully supported by the current design. The evidence does not rule out the possibility that participants interpret increased heartbeat sounds as a generic danger/threat cue rather than as (manipulated) interoceptive input. I also disagree with several other claims, though they are less critical, for example, that the use of specific comparisons without pre-registering them, the use of sensitivity analysis to justify sample size, and the intentional use of only 6 trials per participant.

Conclusion:

To conclude, the authors have shown in their findings that predictions about an upcoming aversive (pain) stimulus - and its subsequent subjective perception - can be altered not only by external expectations, or manipulating the pain cue, as was done in studies so far, but also by manipulating a cue that has fundamental importance to human physiological status, namely heartbeats. Whether this is a manipulation of actual interoception as sensed by the brain is, in my view, left to be proven.

Even if the authors drop this claim, the paper has important implications in several fields of science, ranging from neuroscience prediction-perception research, to pain research, and may have implications for clinical disorders, as the authors propose. Furthermore, it may lead - either the authors or someone else - to further test this interesting question of manipulation of interoception in a different or more controlled manner.

I salute the authors for coming up with this interesting question and encourage them to continue and explore ways to study it and related follow-up questions.

# Referee report: Reviewer #3 (Public review)

**DOI:** 10.7554/eLife.90013.3.sa2

Parrotta et al provide a convincing and thorough revision of their manuscript "Exposure to false cardiac feedback alters pain perception and anticipatory cardiac frequency". The authors addressed my previous concerns regarding theoretical framing and methodological clarity. For example:

They provided additional detail on the experimental design, procedure and statistical analyses.

The predictive coding rationale for the hypotheses has been clarified.

The limitations of the study are discussed comprehensively

Additional analyses were performed to investigate the role of learning effects and across-experiment effects

New supplementary figures allow a closer look at the feedback-related response patterns

In sum, the revisions improve the manuscript. However, some issues remain present.

(1) Potential learning/ habituation effects. In my first review of the manuscript, I raised the concern that learning effects may have contributed to the observed differences between interoceptive & exteroceptive cues.

The authors argue that the small number of six trials per condition could limit aversive effects of differential learning between experiments. However, electric nociceptive stimuli are exceptionally potent in classical conditioning experiments and humans can develop conditioned responses to these types of stimuli after a single trial [1-2]. Therefore, six trials are sufficient to allow for associative or expectancy-based learning processes.

However, the authors are also presenting additional analyses, i.e. LME models which included trial rank as a predictor. While these models do not show a statistically significant learning effect, they do indicate a noteworthy larger effect in earlier trials compared to later ones. However, in my reading, this speaks towards the presence of unspecific effects of attention or arousal. This pattern is compatible with early learning or, alternatively, with non-specific attentional or arousal responses that diminish across repetitions. This is potentially a limitation of the design: repetition-related effects (attention reduction, arousal habituation, early learning) may contribute to the results, and distinguishing between interoceptive inference and non-specific effects remains challenging within this paradigm.

(1) Haesen K, Beckers T, Baeyens F, Vervliet B. One-trial overshadowing: Evidence for fast specific fear learning in humans. Behav Res Ther. 2017 Mar;90:16-24. doi: 10.1016/j.brat.2016.12.001. Epub 2016 Dec 8. PMID: 27960093.

(2) Glenn CR, Lieberman L, Hajcak G. Comparing electric shock and a fearful screaming face as unconditioned stimuli for fear learning. Int J Psychophysiol. 2012 Dec;86(3):214-9. doi: 10.1016/j.ijpsycho.2012.09.006. Epub 2012 Sep 21. PMID: 23007035; PMCID: PMC3627354.

(2) SESOI and power rationale. The authors elaborated on the sensitivity analyses and the rationale of reporting SESOI rather than traditional a-priori power analyses and included this information in the manuscript, which improves transparency.

(3) Unspecific arousal/ attention mechanisms. The authors argue against unspecific arousal mechanisms based on the absence of main effects in pain ratings and heart rate. This reduces the likelihood of a purely unspecific arousal account, however, these unspecific effects may not need to manifest as main effects. Unspecific mechanisms are likely adding (at least residual) effects onto the results.

Regarding attention-based mechanisms, the authors have clarified that in Experiment 2 (exteroceptive cue), the participants are instructed that the sound does not have any relation with their heart rate. If participants did not receive any instructions on the meaning of the knocking sounds, they may have simply ignored it - not unlikely, also because the exteroceptive feedback did not elicit any systematic effect on the outcome variables (minus the slowing of HR with slower exteroceptive feedback, which may reflect noise, altering, multiple comparisons?). Ultimately, how the participants did or did not process the exteroceptive cue is unclear.

(4) The authors provided more context to their hypothesis and strengthened its theoretical motivation (increased pain intensity with incongruent-high cardiac feedback), rooting it in predictive coding accounts of interoception. For instance, their prior study shows that participants report an increased cardiac frequency while anticipating pain. The reasoning behind this study is hence that if pain shapes cardiac perception, cardiac perception should in turn shape pain perception. The introduction has been revised accordingly, adding more references on the interplay between cardiac feedback and pain and emotional responses. While this rooting within the predictive processing framework is now clearly developed, it also underscores a gap between the proposed theoretical mechanism and the current analytical approach. The hypothesis is formulated in a mechanistic, computational-level language, yet the statistical analysis remains primarily descriptive, at a group level, and does not directly test the predictive-coding account.

New concerns introduced by the revision:

(1) Some of the newly added paragraphs interrupt the narrative flow. For example, the justification of the supradiaphragmatic focus based on the BPQ questionnaire feels too long for this section and might fit more naturally in the theoretical background or introduction. Similarly, the predictive-coding paragraph appearing after the hypotheses seems better suited to the earlier conceptual framing rather than following the hypothesis statements. It would be better for the argumentative flow if hypotheses followed from theoretical considerations.

(2) The authors now note that the administration of the BPQ questionnaire was exploratory, explaining the null-results in the methods section as resulting from an underpowered design. But if the design is not appropriate for discovering a connection between self-reported body awareness and pain ratings, why was it administered in the first place? The rationale here is unclear.

(3) The discussion is longer than before and would benefit greatly from streamlining the arguments.

# Author comment: Author response

**DOI:** 10.7554/eLife.90013.3.sa3

**Authors:** Eleonora Parrotta, Patric Bach, Giovanni Pezzulo, Andrea Zaccaro, Mauro Gianni Perrucci, Marcello Costantini, FRANCESCA FERRI

**Affiliations:** Sapienza University of Rome Rome Italy; University of Aberdeen Aberdeen United Kingdom; Institute of Cognitive Sciences and Technologies, National Research Council Rome Italy; University of Chieti-Pescara Chieti Italy; Department of Neuroscience, Imaging and Clinical Sciences, Institute for Advanced Biomedical Technologies ‑ ITAB, "G. d'Annunzio" University of Chieti-Pescara, Chieti, Italy Chieti Italy; University of Chieti-Pescara Chieti Italy; University of Chieti-Pescara Chieti Italy

The following is the authors’ response to the original reviews.

**Public Reviews:**

**Reviewer #1 (Public Review):**

Summary:

I read the paper by Parrotta et al with great interest. The authors are asking an interesting and important question regarding pain perception, which is derived from predictive processing accounts of brain function. They ask: If the brain indeed integrates information coming from within the body (interoceptive information) to comprise predictions about the expected incoming input and how to respond to it, could we provide false interoceptive information to modulate its predictions, and subsequently alter the perception of such input? To test this question, they use pain as the input and the sounds of heartbeats (falsified or accurate) as the interoceptive signal.

Strengths:

I found the question well-established, interesting, and important, with important implications and contributions for several fields, including neuroscience of prediction-perception, pain research, placebo research, and health psychology. The paper is well-written, the methods are adequate, and the findings largely support the hypothesis of the authors. The authors carried out a control experiment to rule out an alternative explanation of their finding, which was important.

Weaknesses:

I will list here one theoretical weakness or concern I had, and several methodological weaknesses.

The theoretical concern regards what I see as a misalignment between a hypothesis and a result, which could influence our understanding of the manipulation of heartbeats, and its meaning: The authors indicate from prior literature and find in their own findings, that when preparing for an aversive incoming stimulus, heartbeats *decrease*. However, in their findings, manipulating the heartbeats that participants hear to be slower than their own prior to receiving a painful stimulus had *no effect* on participants' actual heartbeats, nor on their pain perceptions. What authors did find is that when listening to heartbeats that are *increased* in frequency - that was when their own heartbeats decreased (meaning they expected an aversive stimulus) and their pain perceptions increased.

This is quite complex - but here is my concern: If the assumption is that the brain is collecting evidence from both outside and inside the body to prepare for an upcoming stimulus, and we know that *slowing down* of heartbeats predicts an aversive stimulus, why is it that participants responded in a change in pain perception and physiological response when listened to *increased heartbeats* and not decreased? My interpretation is that the manipulation did not fool the interoceptive signals that the brain collects, but rather the more conscious experience of participants, which may then have been translated to fear/preparation for the incoming stimulus. As the authors indicate in the discussion (lines 704-705), participants do not *know* that decreased heartbeats indicate upcoming aversive stimulus, and I would even argue the opposite - the common knowledge or intuitive response is to increase alertness when we hear increased heartbeats, like in horror films or similar scenarios. Therefore, the unfortunate conclusion is that what the authors assume is a manipulation of interoception - to me seems like a manipulation of participants' alertness or conscious experience of possible danger. I hope the (important) distinction between the two is clear enough because I find this issue of utmost importance for the point the paper is trying to make. If to summarize in one sentence - if it is decreased heartbeats that lead the brain to predict an approaching aversive input, and we assume the manipulation is altering the brain's interoceptive data collection, why isn't it responding to the decreased signal? --> My conclusion is, that this is not in fact a manipulation of interoception, unfortunately

We thank the reviewer for their comment, which gives us the opportunity to clarify what we believe is a theoretical misunderstanding that we have not sufficiently made clear in the previous version of the manuscript. The reviewer suggests that a decreased heart rate itself might act as an internal cue for a forthcoming aversive stimulus, and questions why our manipulation of slower heartbeats then did not produce measurable effects.

The central point is this: decreased heart rate is not a signal the brain uses to predict a threat, but is a consequence of the brain having already predicted the threat. This distinction is crucial. The well-known anticipatory decrease of heartrate serves an allostatic function: preparing the body in advance so that physiological responses to the actual stressor (such as an increase in sympathetic activation) do not overshoot. In other words, the deceleration is an output of the predictive model, not an input from which predictions are inferred. It would be maladaptive for the brain to predict threat through a decrease in heartrate, as this would then call for a further decrease, creating a potential runaway cycle.

Instead, increased heart rate is a salient and evolutionarily conserved cue for arousal, threat, and pain. This association is reinforced both culturally - for example, through the use of accelerating heartbeats in films and media to signal urgency, as R1 mentions - and physiologically, as elevated heart rates reliably occur in response to actual (not anticipated) stressors. Decreased heartrates, in contrast, are reliably associated with the absence of stressors, for example during relaxation and before (and during) sleep. Thus, across various everyday experiences, increased (instead of decreased) heartrates are robustly associated with actual stressors, and there is no a priori reason to assume that the brain would treat decelerating heartrates as cue for threat. As we argued in previous work, “the relationship between the increase in cardiac activity and the anticipation of a threat may have emerged from participants’ first-hand experience of increased heart rates to actual, not anticipated, pain” (Parrotta et al., 2024). The changes in heart rate and pain perception that we hypothesize (and observe) are therefore fully in line with the prior literature on the anticipatory compensatory heartrate response (Bradley et al., 2008, 2005; Colloca et al., 2006; Lykken et al., 1972; Taggart et al., 1976; Tracy et al., 2017; Skora et al., 2022), as well as with Embodied Predictive Coding models (Barrett & Simmons, 2015; Pezzulo, 2014; Seth, 2013; Seth et al., 2012), which assume that our body is regulated through embodied simulations that anticipate likely bodily responses to upcoming events, thereby enabling anticipatory or allostatic regulation of physiological states (Barrett, 2017).

We now add further explanation to this point to the Discussion (lines 740-758) and Introduction (lines 145-148; 154-156) of our manuscript to make this important point clearer.

Barrett, L. F., & Simmons, W. K. (2015). Interoceptive predictions in the brain. Nature reviews neuroscience, 16(7), 419-429.

Barrett, L. F. (2017). The theory of constructed emotion: An active inference account of interoception and categorization. Social cognitive and affective neuroscience, 12(1), 1-23.

Bradley, M. M., Moulder, B., & Lang, P. J. (2005). When good things go bad: The reflex physiology of defense. Psychological science, 16(6), 468-473.

Bradley, M. M., Silakowski, T., & Lang, P. J. (2008). Fear of pain and defensive activation. PAIN, 137(1), 156-163.

Colloca, L., Petrovic, P., Wager, T. D., Ingvar, M., & Benedetti, F. (2010). How the number of learning trials affects placebo and nocebo responses. Pain, 151(2), 430-439.

Lykken, D., Macindoe, I., & Tellegen, A. (1972). Preception: Autonomic response to shock as a function of predictability in time and locus. Psychophysiology, 9(3), 318-333.

Taggart, P., Hedworth-Whitty, R., Carruthers, M., & Gordon, P. D. (1976). Observations on electrocardiogram and plasma catecholamines during dental procedures: The forgotten vagus. British Medical Journal, 2(6039), 787-789.

Tracy, L. M., Gibson, S. J., Georgiou-Karistianis, N., & Giummarra, M. J. (2017). Effects of explicit cueing and ambiguity on the anticipation and experience of a painful thermal stimulus. PloS One, 12(8), e0183650.

Parrotta, E., Bach, P., Perrucci, M. G., Costantini, M., & Ferri, F. (2024). Heart is deceitful above all things: Threat expectancy induces the illusory perception of increased heartrate. Cognition, 245, 105719.

Pezzulo, G. (2014). Why do you fear the bogeyman? An embodied predictive coding model of perceptual inference. Cognitive, Affective & Behavioral Neuroscience, 14(3), 902-911.

Seth, A., Suzuki, K., & Critchley, H. (2012). An Interoceptive Predictive Coding Model of Conscious Presence. Frontiers in Psychology, 2. here

Seth, A. K. (2013). Interoceptive inference, emotion, and the embodied self. Trends in Cognitive Sciences, 17(11), 565-573.

Skora, L. I., Livermore, J. J. A., & Roelofs, K. (2022). The functional role of cardiac activity in perception and action. Neuroscience & Biobehavioral Reviews, 104655.

I will add that the control experiment - with an exteroceptive signal (knocking of wood) manipulated in a similar manner - could be seen as evidence of the fact that heartbeats are regarded as an interoceptive signal, and it is an important control experiment, however, to me it seems that what it is showing is the importance of human-relevant signals to pain prediction/perception, and not directly proves that it is considered interoceptive. For example, it could be experienced as a social cue of human anxiety/fear etc, and induce alertness.

The reviewer asks us to consider whether our measured changes in pain response happen not because the brain treats the heartrate feedback in Experiment 1 as interoceptive stimulus, but because heartbeat sounds could have signalled threat on a more abstract, perhaps metacognitive or affective, level, in contrast to the less visceral control sounds in Experiment 2. We deem this highly unlikely for several reasons.

First, as we point out in our response to Reviewer 3 (Point 3), if this were the case, the different sounds in both experiments should have induced overall (between-experiment) differences in pain perception and heart rate, induced by the (supposedly) generally more threatening heart beat sounds. However, when we added such comparisons, no such between-experiment differences were obtained (See Results Experiment 2, and Supplementary Materials, Cross-experiment analysis between-subjects model). Instead, we only find a significant interaction between experiment and feedback (faster, slower). Thus, it is not the heartbeat sounds per se that induce the measured changes to pain perception, but the modulation of their rate, and that identical changes to the rate of non-heartrate sounds produce no such effects. In other words, pain perception is sensitive to a change in heart rate feedback, as we predicted, instead of the overall presence of heartbeat sounds (as one would need to predict if heart beat sounds had more generally induced threat or stress).

Second, one may suspect that it is precisely the acceleration of heartrate feedback that could act as cue to arousal, while accelerated exteroceptive feedback would not. However, if this were the case, one would need to predict a general heart rate increase with accelerated feedback, as this is the general physiological marker of increasing alertness and arousal (e.g. Tousignant-Laflamme et al., 2005; Terkelsen et al., 2005; for a review, see Forte et al., 2022). However, the data shows the opposite, with real heartrates decreasing when the heartrate feedback increases. This result is again fully in line with the predicted interoceptive consequences of accelerated heartrate feedback, which mandates an immediate autonomic regulation, especially when preparing for an anticipated stressor.

Third, our view is further supported by neurophysiological evidence showing that heartbeat sounds, particularly under the belief they reflect one’s own body, are not processed merely as generic aversive or “human-relevant” signals. For instance, Vicentin et al. (2024) showed that simulated faster heartbeat sounds elicited stronger EEG alpha-band suppression, indicative of increased cortical activation over frontocentral and right frontal areas, compatible with the localization of brain regions contributing to interoceptive processes (Kleint et al., 2015). Importantly, Kleint et al. also demonstrated via fMRI that heartbeat sounds, compared to acoustically matched tones, selectively activate bilateral anterior insula and frontal operculum, key hubs of the interoceptive network. This suggests that the semantic identity of the sound as a heartbeat is sufficient to elicit internal body representations, despite its exteroceptive nature. Further evidence comes from van Elk et al. (2014), who found that heartbeat sounds suppress the auditory N1 component, a neural marker of sensory attenuation typically associated with self-generated or predicted stimuli. The authors interpret this as evidence that the brain treats heartbeat sounds as internally predicted bodily signals, supporting interoceptive predictive coding accounts in which exteroceptive cues (i.e., auditory cardiac feedback) are integrated with visceral information to generate coherent internal body representations.

Finally, it is worth noting that the manipulation of heartrate feedback in our study elicited measurable compensatory changes in participants’ actual heart rate. This is striking compared to our previous work (Parrotta et al., 2024), wherein we used a highly similar design as here, combined with a very strong threat manipulation. Specifically, we presented participants with highly salient threat cues (knives directed at an anatomical depiction of a heart), which predicted forthcoming pain with 100% validity (compared to flowers that did predict the absence of pain with 100%). In other words, these cues perfectly predicted actual pain, through highly visceral stimuli. Nevertheless, we found no measurable decrease in actual heartrate. From an abstract threat perspective, it is therefore striking that the much weaker manipulation of slightly increased or decreased heartrates we used here would induce such a change. The difference therefore suggests that what caused the response here is not due to an abstract feeling of threat, but because the brain indeed treated the increased heartrate feedback as an interoceptive signal for (stressor-induced) sympathetic activation, which would then be immediately down-regulated.

Together, we hope you agree that these considerations make a strong case against a non-specific, arousal or alertness-related explanation of our data. We now make this point clearer in the new paragraph of the Discussion (Accounting for general unspecific contributionslines 796-830), and have added the relevant between experiment comparisons to the Results of Experiment 2.

Forte, G., Troisi, G., Pazzaglia, M., Pascalis, V. D., & Casagrande, M. (2022). Heart rate variability and pain: a systematic review. Brain sciences, 12(2), 153.

Vicentin, S., Guglielmi, S., Stramucci, G., Bisiacchi, P., & Cainelli, E. (2024). Listen to the beat: behavioral and neurophysiological correlates of slow and fast heartbeat sounds. International Journal of Psychophysiology, 206, 112447.

Kleint, N. I., Wittchen, H. U., & Lueken, U. (2015). Probing the interoceptive network by listening to heartbeats: an fMRI study. PloS one, 10(7), e0133164.

Parrotta, E., Bach, P., Perrucci, M. G., Costantini, M., & Ferri, F. (2024). Heart is deceitful above all things: Threat expectancy induces the illusory perception of increased heartrate. Cognition, 245, 105719.

Terkelsen, A. J., Mølgaard, H., Hansen, J., Andersen, O. K., & Jensen, T. S. (2005). Acute pain increases heart rate: differential mechanisms during rest and mental stress. Autonomic Neuroscience, 121(1-2), 101-109.

Tousignant-Laflamme, Y., Rainville, P., & Marchand, S. (2005). Establishing a link between heart rate and pain in healthy subjects: a gender effect. The journal of pain, 6(6), 341-347.

van Elk, M., Lenggenhager, B., Heydrich, L., & Blanke, O. (2014). Suppression of the auditory N1-component for heartbeat-related sounds reflects interoceptive predictive coding. Biological psychology, 99, 172-182.

Several additional, more methodological weaknesses include the very small number of trials per condition - the methods mention 18 test trials per participant for the 3 conditions, with varying pain intensities, which are later averaged (and whether this is appropriate is a different issue). This means 6 trials per condition, and only 2 trials per condition and pain intensity. I thought that this number could be increased, though it is not a huge concern of the paper. It is, however, needed to show some statistics about the distribution of responses, given the very small trial number (see recommendations for authors). The sample size is also rather small, on the verge of "just right" to meet the required sample size according to the authors' calculations.

We provide detailed responses to these points in the “Recommendations for The Authors” section, where each of these issues is addressed point by point in response to the specific questions raised.

Finally, and just as important, the data exists to analyze participants' physiological responses (ECG) after receiving the painful stimulus - this could support the authors' claims about the change in both subjective and objective responses to pain. It could also strengthen the physiological evidence, which is rather weak in terms of its effect. Nevertheless, this is missing from the paper.

This is indeed an interesting point, and we agree that analyzing physiological responses such as ECG following the painful stimulus could offer additional insights into the objective correlates of pain. However, it is important to clarify that the experiment was not designed to investigate post-stimulus physiological responses. Our primary focus was on the anticipatory processes leading up to the pain event. Notably, in the time window immediately following the stimulus - when one might typically expect to observe physiological changes such as an increase in heart rate - participants were asked to provide subjective ratings of their nociceptive experience. It is therefore not a “clean” interval that would lend itself for measurement, especially as a substantial body of evidence indicates that one’s heart rate is strongly modulated by higher-order cognitive processes, including attentional control, executive functioning, decision-making and action itself (e.g., Forte et al., 2021a; Forte et al., 2021b; Luque-Casado et al., 2016).

This limitation is particularly important as the induced change in pain ratings by our heart rate manipulation is substantially smaller than the changes in heart rate induced by actual pain (e.g., Loggia et al., 2011). To confirm this for our study, we simply estimated how much change in heart rate is produced by a change in actual stimulus intensity in the initial no feedback phase of our experiment. There, we find that a change between stimulus intensities 2 and 4 induces a NPS change of 32.95 and a heart rate acceleration response of 1.19 (difference in heart rate response relative to baseline, Colloca et al., 2006), d = .52, p < .001. The change of NPS induced by our implicit heart rate manipulation, however, is only a seventh of this (4.81 on the NPS). This means that the expected effect size of heart rate acceleration produced by our manipulation would only be d = .17. A power analysis, using GPower, reveals that a sample size of n = 266 would be required to detect such an effect, if it exists. Thus, while we agree that this is an exciting hypothesis to be tested, it requires a specifically designed study, and a much larger sample than was possible here.

Colloca, L., Benedetti, F., & Pollo, A. (2006). Repeatability of autonomic responses to pain anticipation and pain stimulation. European Journal of Pain, 10(7), 659-665.

Forte, G., Morelli, M., & Casagrande, M. (2021a). Heart rate variability and decision-making: Autonomic responses in making decisions. Brain sciences, 11(2), 243.

Forte, G., Favieri, F., Oliha, E. O., Marotta, A., & Casagrande, M. (2021b). Anxiety and attentional processes: the role of resting heart rate variability. Brain sciences, 11(4), 480.

Loggia, M. L., Juneau, M., & Bushnell, M. C. (2011). Autonomic responses to heat pain: Heart rate, skin conductance, and their relation to verbal ratings and stimulus intensity. PAIN, 152(3), 592-598.

Luque-Casado, A., Perales, J. C., Cárdenas, D., & Sanabria, D. (2016). Heart rate variability and cognitive processing: The autonomic response to task demands. Biological psychology, 113, 83-90

I have several additional recommendations regarding data analysis (using an ANOVA rather than multiple t-tests, using raw normalized data rather than change scores, questioning the averaging across 3 pain intensities) - which I will detail in the "recommendations for authors" section.

We provide detailed responses to these points in the “Recommendations for The Authors” section, where each of these issues is addressed point by point in response to the specific questions raised.

Conclusion:

To conclude, the authors have shown in their findings that predictions about an upcoming aversive (pain) stimulus - and its subsequent subjective perception - can be altered not only by external expectations, or manipulating the pain cue, as was done in studies so far, but also by manipulating a cue that has fundamental importance to human physiological status, namely heartbeats. Whether this is a manipulation of actual interoception as sensed by the brain is - in my view - left to be proven.

Still, the paper has important implications in several fields of science ranging from neuroscience prediction-perception research, to pain and placebo research, and may have implications for clinical disorders, as the authors propose. Furthermore, it may lead - either the authors or someone else - to further test this interesting question of manipulation of interoception in a different or more controlled manner.

I salute the authors for coming up with this interesting question and encourage them to continue and explore ways to study it and related follow-up questions.

We sincerely thank the reviewer for the thoughtful and encouraging feedback. We hope our responses to your points below convince you a bit more that what we are measuring does indeed capture interoceptive processes, but we of course fully acknowledge that additional measures - for example from brain imaging (or computational modelling, see Reviewer 3) - could further support our interpretation, and highlights in the Limitations and Future directions section.

**Reviewer #2 (Public Review):**

In this manuscript, Parrotta et al. tested whether it is possible to modulate pain perception and heart rate by providing false HR acoustic feedback before administering electrical cutaneous shocks. To this end, they performed two experiments. The first experiment tested whether false HR acoustic feedback alters pain perception and the cardiac anticipatory response. The second experiment tested whether the same perceptual and physiological changes are observed when participants are exposed to a non-interoceptive feedback. The main results of the first experiment showed a modulatory effect for faster HR acoustic feedback on pain intensity, unpleasantness, and cardiac anticipatory response compared to a control (acoustic feedback congruent to the participant's actual HR). However, the results of the second experiment also showed an increase in pain ratings for the faster non-interoceptive acoustic feedback compared to the control condition, with no differences in pain unpleasantness or cardiac response.

The main strengths of the manuscript are the clarity with which it was written, and its solid theoretical and conceptual framework. The researchers make an in-depth review of predictive processing models to account for the complex experience of pain, and how these models are updated by perceptual and active inference. They follow with an account of how pain expectations modulate physiological responses and draw attention to the fact that most previous studies focus on exteroceptive cues. At this point, they make the link between pain experience and heart rate changes, and introduce their own previous work showing that people may illusorily perceive a higher cardiac frequency when expecting painful stimulation, even though anticipating pain typically goes along with a decrease in HR. From here, they hypothesize that false HR acoustic feedback evokes more intense and unpleasant pain perception, although the actual HR actually decreases due to the orienting cardiac response. Furthermore, they also test the hypothesis that an exteroceptive cue will lead to no (or less) changes in those variables. The discussion of their results is also well-rooted in the existing bibliography, and for the most part, provides a credible account of the findings.

Thank you for the clear and thoughtful review. We appreciate your positive comments on the manuscript’s clarity, theoretical framework, and interpretation of results.

The main weaknesses of the manuscript lies in a few choices in methodology and data analysis that hinder the interpretation of the results and the conclusions as they stand.

The first peculiar choice is the convoluted definition of the outcomes. Specifically, pain intensity and unpleasantness are first normalized and then transformed into variation rates (sic) or deltas, which makes the interpretation of the results unnecessarily complicated. This is also linked to the definitions of the smallest effect of interest (SESOI) in terms of these outcomes, which is crucial to determining the sample size and gauging the differences between conditions. However, the choice of SESOI is not properly justified, and strangely, it changes from the first experiment to the second.

We thank the reviewer for this important observation. In the revised manuscript, we have made substantial changes and clarifications to address both aspects of this concern: (1) the definition of outcome variables and their normalization, and (2) the definition of the SESOI.

First, As explained in our response to Reviewer #1, we have revised the analyses and removed the difference-based change scores from the main results, addressing concerns about interpretability. However, we retained the normalization procedure: all variables (heart rate, pain intensity, unpleasantness) are normalized relative to the no-feedback baseline using a standard proportional change formula (X−bX)/bX(X - bX)/bX(X−bX)/bX, where X is the feedback-phase mean and bX is the no-feedback baseline. This is a widely used normalization procedure (e.g., Bartolo et al., 2013; Cecchini et al., 2020). This method controls for interindividual variability by expressing responses relative to each participant’s own baseline. The resulting normalized values are then used directly in all analyses, and not further transformed into deltas.

To address potential concerns about this baseline correction approach and its interpretability, we also conducted a new set of supplementary analyses (now reported in the supplementary materials) that include the no-feedback condition explicitly in the models, rather than treating it as a baseline for normalization. These models confirm that our main effects are not driven by the choice of normalization and hold even when no-feedback is analyzed as an independent condition. The new analyses and results are now reported in the Supplementary Materials.

Second, concerning the SESOI values and their justification: The difference in SESOI values between Experiment 1 and Experiment 2 reflects the outcome of sensitivity analyses conducted for each dataset separately, rather than a post-hoc reinterpretation of our results. Specifically, we followed current methodological recommendations (Anderson, Kelley & Maxwell, 2017; Albers & Lakens, 2017; Lakens, 2022), which advise against estimating statistical power based on previously published effect sizes, especially when working with novel paradigms or when effect sizes in the literature may be inflated or imprecise. Instead, we used the sensitivity analysis function in G*Power (Version 3.1) to determine the smallest effect size our design was capable of detecting with high statistical power (90%), given the actual sample size, test type, and alpha level used in each experiment. This is a prospective, design-based estimation rather than a post-hoc analysis of observed effects. The slight differences in SESOI are due to more participants falling below our exclusions criteria in Experiment 2, leading to slightly larger effect sizes that can be detected (d = 0.62 vs d = 0.57). Importantly, both experiments remain adequately powered to detect effects of a size commonly reported in the literature on top-down pain modulation. For instance, Iodice et al. (2019) reported effects of approximately d = 0.7, which is well above the minimum detectable thresholds of our designs.

We have now clarified the logic in the Participant section of Experiment 1 (193-218).

Anderson, S. F., Kelley, K., & Maxwell, S. E. (2017). Sample-Size Planning for More Accurate Statistical Power: A Method Adjusting Sample Effect Sizes for Publication Bias and Uncertainty. Psychological Science, 28(11), 1547-1562.

Bartolo, M., Serrao, M., Gamgebeli, Z., Alpaidze, M., Perrotta, A., Padua, L., Pierelli, F., Nappi, G., & Sandrini, G. (2013). Modulation of the human nociceptive flexion reflex by pleasant and unpleasant odors. PAIN, 154(10), 2054-2059.

Cecchini, M. P., Riello, M., Sandri, A., Zanini, A., Fiorio, M., & Tinazzi, M. (2020). Smell and taste dissociations in the modulation of tonic pain perception induced by a capsaicin cream application. European Journal of Pain, 24(10), 1946-1955.

Lakens, D. (2022). Sample size justification. Collabra: psychology, 8(1), 33267.

Albers, C., & Lakens, D. (2018). When power analyses based on pilot data are biased: Inaccurate effect size estimators and follow-up bias. Journal of experimental social psychology, 74, 187-195.

Furthermore, the researchers propose the comparison of faster vs. slower delta HR acoustic feedback throughout the manuscript when the natural comparison is the incongruent vs. the congruent feedback.

We very much disagree that the natural comparison is congruent vs incongruent feedback. First, please note that congruency simply refers to whether the heartrate feedback was congruent with (i.e., matched) the participant’s heartrate measurements in the no feedback trials, or whether it was incongruent, and was therefore either faster or slower than this baseline frequency. As such, simply comparing congruent with incongruent feedback could only indicate that pain ratings change when the feedback does not match the real heart rate, irrespective of whether it is faster or slower. Such a test can therefore only reveal potential general effects of surprise or salience, when the feedback heartrate does not match the real one.

We therefore assume that the reviewer specifically refers to the comparison of congruent vs incongruent faster feedback. However, this is not a good test either, as this comparison is, by necessity, confounded with the factor of surprise described above. In other words, if a difference would be found, it would not be clear if it emerges because, as we assume, that faster feedback is represented as an interoceptive signal for threat, or simply because participants are surprised about heartrate feedback that diverges from their real heartrate. Note that even a non-significant result in the analogous comparison of congruent vs incongruent slower feedback would not be able to resolve this confound, as in null hypothesis testing the absence of a significant effect does, per definition, not indicate that there is no effect - only that it could not be detected here.

Instead, the only possible test of our hypothesis is the one we have designed our experiment around and focussed on with our central t-test: the comparison of incongruent faster with incongruent slower feedback. This keeps any possible effects of surprise/salience from generally altered feedback constant and allows us to test our specific hypothesis: that real heart rates will decrease and pain ratings will increase when receiving false interoceptive feedback about increased compared to decreasing heartrates. Note that this test of faster vs slower feedback is also statistically the most appropriate, as it collapses our prediction onto a single and highest-powered hypothesis test: As faster and slower heartrate feedback are assumed to induce effects in the opposite direction, the effect size of their difference is, per definition, double than the averaged effect size for the two separate tests of faster vs congruent feedback and slower vs congruent feedback.

That being said, we also included comparisons with the congruent condition in our revised analysis, in line with the reviewer’s suggestion and previous studies. These analyses help explore potential asymmetries in the effect of false feedback. While faster feedback (both interoceptive and exteroceptive) significantly modulated pain relative to congruent feedback, the slower feedback did not, consistent with previous literature showing stronger effects for arousal-increasing cues (e.g., Valins, 1966; Iodice et al., 2019). To address this point, in the revised manuscript we have added a paragraph to the Data Analysis section of Experiment 1 (lines 405-437) to make this logic clearer.

Valins, S. (1966). Cognitive effects of false heart-rate feedback. Journal of personality and social psychology, 4(4), 400.

Iodice, P., Porciello, G., Bufalari, I., Barca, L., & Pezzulo, G. (2019). An interoceptive illusion of effort induced by false heart-rate feedback. Proceedings of the National Academy of Sciences, 116(28), 13897-13902.

This could be influenced by the fact that the faster HR exteroceptive cue in experiment 2 also shows a significant modulatory effect on pain intensity compared to congruent HR feedback, which puts into question the hypothesized differences between interoceptive vs. exteroceptive cues. These results could also be influenced by the specific choice of exteroceptive cue: the researchers imply that the main driver of the effect is the nature of the cue (interoceptive vs. exteroceptive) and not its frequency. However, they attempt to generalize their findings using knocking wood sounds to all possible sounds, but it is possible that some features of these sounds (e.g., auditory roughness or loomingness) could be the drivers behind the observed effects.

We appreciate this thoughtful comment. We agree that low-level auditory features can potentially introduce confounds in the experimental design, and we acknowledge the importance of distinguishing these factors from the higher-order distinction that is central to our study: whether the sound is perceived as interoceptive (originating from within the body) or exteroceptive (perceived as external). To this end, the knocking sound was chosen not for its specific acoustic profile, but because it lacked bodily relevance, thus allowing us to test whether the same temporal manipulations (faster, congruent, slower) would have different effects depending on whether the cue was interpreted as reflecting an internal bodily state or not. In this context, the exteroceptive cue served as a conceptual contrast rather than an exhaustive control for all auditory dimensions.

Several aspects of our data make it unlikely that the observed effects are driven by unspecific acoustic characteristics of the sounds used in the exteroceptive and interoceptive experiments (see also our responses to Reviewer 1 and Reviewer 3 who raised similar points).

First, if the knocking sound had inherent acoustic features that strongly influenced perception or physiological responses, we would expect it to have produced consistent effects across all feedback conditions (Faster, Slower, Congruent), regardless of the interpretive context. This would have manifested as an overall difference between experiments in the between-subjects analyses and in the supplementary mixed-effects models that included Experiment as a fixed factor. Yet, we observed no such main effects in any of our variables. Instead, significant differences emerged only in specific theoretically predicted comparisons (e.g., Faster vs. Slower), and critically, these effects depended on the cue type (interoceptive vs. exteroceptive), suggesting that perceived bodily relevance, rather than a specific acoustic property, was the critical modulator. In other words, any alternative explanation based on acoustic features would need to be able to explain why these acoustic properties would induce not an overall change in heart rate and pain perception (i.e., similarly across slower, faster, and congruent feedback), but the brain’s response to changes in the rate of this feedback – increasing pain ratings and decreasing heartrates for faster relative to slower feedback. We hope you agree that a simple effect of acoustic features would not predict such a sensitivity to the rate with which the sound was played.

Please refer to our responses to Reviewers 1 and 2 for further aspects of the data, arguing strongly against other features associated with the sounds (e.g., alertness, arousal) could be responsible for the results, as the data pattern again goes in the opposite direction than that predicted by such accounts (e.g., faster heartrate feedback decreased real heartrate, instead of increasing them, as would be expected if accelerated heartrate feedback increased arousal).

Finally, to further support this interpretation, we refer to neurophysiological evidence showing that heartbeat sounds are not processed as generic auditory signals, but as internal, bodily relevant cues especially when believed to reflect one’s own physiological state. For instance, fMRI research (Kleint et al., 2015) shows that heartbeat sounds engage key interoceptive regions such as the anterior insula and frontal operculum more than acoustically matched control tones. EEG data (Vicentin et al., 2024) showed that faster heartbeat sounds produce stronger alpha suppression over frontocentral areas, suggesting enhanced processing in networks associated with interoceptive attention. Moreover, van Elk et al. (2014) found that heartbeat sounds attenuate the auditory N1 response, a neural signature typically linked to self-generated or predicted bodily signals. These findings consistently demonstrate that heartbeats sounds are processed as interoceptive and self-generated signals, which is in line with our rationale that the critical factor at play concern whether it is semantically perceived as reflecting one’s own bodily state, rather than the physical properties of the sound.

We now explicitly discuss these issues in the revised Discussion section (lines 740-758).

Kleint, N. I., Wittchen, H. U., & Lueken, U. (2015). Probing the interoceptive network by listening to heartbeats: an fMRI study. PloS one, 10(7), e0133164.

van Elk, M., Lenggenhager, B., Heydrich, L., & Blanke, O. (2014). Suppression of the auditory N1-component for heartbeat-related sounds reflects interoceptive predictive coding. Biological psychology, 99, 172-182.

Vicentin, S., Guglielmi, S., Stramucci, G., Bisiacchi, P., & Cainelli, E. (2024). Listen to the beat: behavioral and neurophysiological correlates of slow and fast heartbeat sounds. International Journal of Psychophysiology, 206, 112447.

Finally, it is noteworthy that the researchers divided the study into two experiments when it would have been optimal to test all the conditions with the same subjects in a randomized order in a single cross-over experiment to reduce between-subject variability. Taking this into consideration, I believe that the conclusions are only partially supported by the evidence. Despite of the outcome transformations, a clear effect of faster HR acoustic feedback can be observed in the first experiment, which is larger than the proposed exteroceptive counterpart. This work could be of broad interest to pain researchers, particularly those working on predictive coding of pain.

We appreciate the reviewer’s suggestion regarding a within-subject crossover design. While such a design indeed offers increased statistical power by reducing interindividual variability (Charness, Gneezy, & Kuhn, 2012), we intentionally opted for a between-subjects design due to theoretical and methodological considerations specific to studies involving deceptive feedback. Most importantly, carryover effects are a major concern in deception paradigms. Participants exposed to one type of feedback initially (e.g., interoceptive), and then the other (exteroceptive) would be more likely to develop suspicion or adaptive strategies that would alter their responses. Such expectancy effects could contaminate results in a crossover design, particularly when participants realize that feedback is manipulated. In line with this idea, past studies on false cardiac feedback (e.g., Valins, 1966; Pennebaker & Lightner, 1980) often employed between-subjects or blocked designs to mitigate this risk.

Pennebaker, J. W., & Lightner, J. M. (1980). Competition of internal and external information in an exercise setting. Journal of personality and social psychology, 39(1), 165.

Valins, S. (1966). Cognitive effects of false heart-rate feedback. Journal of personality and social psychology, 4(4), 400.

**Reviewer #3 (Public Review):**

In their manuscript titled "Exposure to false cardiac feedback alters pain perception and anticipatory cardiac frequency", Parrotta and colleagues describe an experimental study on the interplay between false heart rate feedback and pain experience in healthy, adult humans. The experimental design is derived from Bayesian perspectives on interoceptive inference. In Experiment 1 (N=34), participants rated the intensity and unpleasantness of an electrical pulse presented to their middle fingers. Participants received auditory cardiac feedback prior to the electrical pulse. This feedback was congruent with the participant's heart rate or manipulated to have a higher or lower frequency than the participant's true heart rate (incongruent high/ low feedback). The authors find heightened ratings of pain intensity and unpleasantness as well as a decreased heart rate in participants who were exposed to the incongruent-high cardiac feedback. Experiment 2 (N=29) is equivalent to Experiment 1 with the exception that non-interoceptive auditory feedback was presented. Here, mean pain intensity and unpleasantness ratings were unaffected by feedback frequency.

Strengths:

The authors present interesting experimental data that was derived from modern theoretical accounts of interoceptive inference and pain processing.

(1) The motivation for the study is well-explained and rooted within the current literature, whereas pain is the result of a multimodal, inferential process. The separation of nociceptive stimulation and pain experience is explained clearly and stringently throughout the text.

(2) The idea of manipulating pain-related expectations via an internal, instead of an external cue, is very innovative.

(3) An appropriate control experiment was implemented, where an external (non-physiological) auditory cue with parallel frequency to the cardiac cue was presented.

(4) The chosen statistical methods are appropriate, albeit averaging may limit the opportunity for mechanistic insight, see weaknesses section.

(5) The behavioral data, showing increased unpleasantness and intensity ratings after exposure to incongruent-high cardiac feedback, but not exteroceptive high-frequency auditory feedback, is backed up by ECG data. Here, the decrease in heart rate during the incongruent-high condition speaks towards a specific, expectation-induced physiological effect that can be seen as resulting from interoceptive inference.

We thank the reviewer for their positive feedback. We are glad that the study’s theoretical foundation, innovative design, appropriate control conditions, and convergence of behavioral and physiological data were well received.

Weaknesses:

Additional analyses and/ or more extensive discussion are needed to address these limitations:

(1) I would like to know more about potential learning effects during the study. Is there a significant change in ∆ intensity and ∆ unpleasantness over time; e.g. in early trials compared to later trials? It would be helpful to exclude the alternative explanation that over time, participants learned to interpret the exteroceptive cue more in line with the cardiac cue, and the effect is driven by a lack of learning about the slightly less familiar cue (the exteroceptive cue) in early trials. In other words, the heartbeat-like auditory feedback might be "overlearned", compared to the less naturalistic tone, and more exposure to the less naturalistic cue might rule out any differences between them w.r.t. pain unpleasantness ratings.

We thank the reviewer for raising this important point. Please note that the repetitions in our task were relatively limited (6 trials per condition), which limits the potential influence of such differential learning effects between experiments. To address this concern, we performed an additional analysis, reported in the Supplementary Materials, using a Linear Mixed-Effects Model approach. This method allowed us to include "Trial" (the rank order of each trial) as a variable to account for potential time-on-task effects such as learning, adaptation, or fatigue (e.g., Möckel et al., 2015). All feedback conditions (no-feedback, congruent, faster, slower) and all stimulus intensity levels were included.

Specifically, we tested the following models:

Likert Pain Unpleasantness Ratings ~ Experiment × Feedback × StimInt × Trial + (StimInt + Trial | Subject)

Numeric Pain Scale of Intensity Ratings ~ Experiment × Feedback × StimInt × Trial + (StimInt + Trial | Subject)

In both models, no significant interactions involving Trial × Experiment or Trial × Feedback × Experiment were found. Instead, we just find generally larger effects in early trials compared to later ones (Main effect of Trial within each Experiment), similar to other cognitive illusions where repeated exposure diminishes effects. Thus, although some unspecific changes over time may have occurred (e.g., due to general task exposure), these changes did not differ systematically across experimental conditions (interoceptive vs. exteroceptive) or feedback types. However, we are fully aware that the absence of significant higher-order interactions does not conclusively rule out the possibility of learning-related effects. It is possible that our models lacked the statistical power to detect more subtle or complex time-dependent modulations, particularly if such effects differ in magnitude or direction across feedback conditions.

We report the full description of these analyses and results in the Supplementary materials 1. Cross-experiment analysis (between-subjects model).

(2) The origin of the difference in Cohen's d (Exp. 1: .57, Exp. 2: .62) and subsequently sample size in the sensitivity analyses remains unclear, it would be helpful to clarify where these values are coming from (are they related to the effects reported in the results? If so, they should be marked as post-hoc analyses).

Following recommendations (Anderson, Kelley & Maxwell, 2017; Albers & Lakens, 2017), we do not report theoretical power based on previously reported effect sizes as this neglects uncertainty around effect size measurements, especially for new effects for which no reliable expected effect size estimates can be derived across the literature. Instead, the power analysis is based on a sensitivity analysis, conducted in G*Power (Version 3.1). Importantly, these are not post-hoc analyses, as they are not based on observed effect sizes in our study, but derived a priori. Sensitivity analyses estimate effect sizes that our design is well-powered (90%) to detect (i.e. given target power, sample size, type of test), for the crucial comparison between faster and slower feedback in both experiments (Lakens, 2022). Following recommendations, we also report the smallest effect size this test can in principle detect in our study (SESOI, Lakens, 2022). This yields effect sizes of d = .57 in Experiment 1 and d = .62 in Experiment 2 at 90% power and SESOIs of d = .34 and .37, respectively. Note that values are slightly higher in Experiment 2, as more participants were excluded based on our exclusion criteria. Importantly, detectable effect sizes in both experiments are smaller than reported effect sizes for comparable top-down effects on pain measurements of d = .7 (Iodice et al., 2019). We have now added more information to the power analysis sections to make this clearer (lines 208-217).

Albers, C., & Lakens, D. (2018). When power analyses based on pilot data are biased: Inaccurate effect size estimators and follow-up bias. Journal of experimental social psychology, 74, 187-195.

Anderson, S. F., Kelley, K., & Maxwell, S. E. (2017). Sample-Size Planning for More Accurate Statistical Power: A Method Adjusting Sample Effect Sizes for Publication Bias and Uncertainty. Psychological Science, 28(11), 1547-1562.

Lakens, D. (2022). Sample size justification. Collabra: psychology, 8(1), 33267.

(3) As an alternative explanation, it is conceivable that the cardiac cue may have just increased unspecific arousal or attention to a larger extent than the exteroceptive cue. It would be helpful to discuss the role of these rather unspecific mechanisms, and how it may have differed between experiments.

We thank the reviewer for raising this important point. We agree that, in principle, unspecific mechanisms such as increased arousal or attention driven by cardiac feedback could be an alternative explanation for the observed effects. However, several aspects of our data indicate that this is unlikely:

(1) No main effect of Experiment on pain ratings:

If the cardiac feedback had simply increased arousal or attention in a general (non-specific) way, we would expect a main effect of Experiment (i.e., interoceptive vs exteroceptive condition) on pain intensity or unpleasantness ratings, regardless of feedback frequency. However, such a main effect was never observed when we compared between experiments (see between-experiment t-tests in results, and in supplementary analyses). Instead, effects were specific to the manipulation of feedback frequency.

(2) Heart rate as an arousal measure:

Heart rate (HR) is a classical physiological index of arousal. If there had been an unspecific increase in arousal in the interoceptive condition, we would expect a main effect of Experiment on HR. However, no such main effect was found. Instead, our HR analyses revealed a significant interaction between feedback and experiment, suggesting that HR changes depended specifically on the feedback manipulation rather than reflecting a general arousal increase.

(3) Arousal predicts faster, not slower, heart rates

In Experiment 1, faster interoceptive cardiac feedback led to a slowdown in heartrates both when compared to slower feedback and to congruent cardiac feedback. This is in line with the predicted compensatory response to faster heart rates. In contrast, if faster feedback would have only generally increased arousal, heart rates should have increased instead of decreased, as indicated by several prior studies (Tousignant-Laflamme et al., 2005; Terkelsen et al., 2005; for a review, see Forte et al., 2022), predicting the opposite pattern of responses than was found in Experiment 1.

Taken together, these findings indicate that the effects observed are unlikely to be driven by unspecific arousal or attention mechanisms, but rather are consistent with feedback-specific modulations, in line with our interoceptive inference framework.

We have now integrated these considerations in the revised discussion (lines 796-830), and added the relevant between-experiment comparisons to the Results of Experiment 2 and the supplementary analysis.

Terkelsen, A. J., Mølgaard, H., Hansen, J., Andersen, O. K., & Jensen, T. S. (2005). Acute pain increases heart rate: differential mechanisms during rest and mental stress. Autonomic Neuroscience, 121(1-2), 101-109.

Tousignant-Laflamme, Y., Rainville, P., & Marchand, S. (2005). Establishing a link between heart rate and pain in healthy subjects: a gender effect. The journal of pain, 6(6), 341-347.

Forte, G., Troisi, G., Pazzaglia, M., Pascalis, V. D., & Casagrande, M. (2022). Heart rate variability and pain: a systematic review. Brain sciences, 12(2), 153.

(4) The hypothesis (increased pain intensity with incongruent-high cardiac feedback) should be motivated by some additional literature.

We thank the reviewer for this helpful suggestion. Please note that the current phenomenon was tested in this experiment for the first time. Therefore, there is no specific prior study that motivated our hypotheses; they were driven theoretically, and derived from our model of interoceptive integration of pain and cardiac perception. The idea that accelerated cardiac feedback (relative to decelerated feedback) will increase pain perception and reduce heart rates is grounded on Embodied Predictive coding frameworks. Accordingly, expectations and signals from different sensory modalities (sensory, proprioceptive, interoceptive) are integrated both to efficiently infer crucial homeostatic and physiological variables, such as hunger, thirst, and, in this case, pain, and regulate the body’s own autonomic responses based on these inferences.

Within this framework, the concept of an interoceptive schema (Tschantz et al., 2022; Iodice et al., 2019; Parrotta et al., 2024; Schoeller et al., 2022) offers the basis for understanding interoceptive illusions, wherein inferred levels of interoceptive states (i.e., pain) deviate from the actual physiological state. Cardiac signals conveyed by the feedback manipulation act as a misleading prior, shaping the internal generative model of pain. Specifically, an increased heart rate may signal a state of threat, establishing a prior expectation of heightened pain. Building on predictive models of interoception, we predict that this cardiac prior is integrated with interoceptive (i.e., actual nociceptive signal) and exteroceptive inputs (i.e., auditory feedback input), leading to a subjective experience of increased pain even when there is no corresponding increase in the nociceptive input.

This idea is not completely new, but it is based on our previous findings of an interoceptive cardiac illusion driven by misleading priors about anticipated threat (i.e., pain). Specifically, in Parrotta et al. (2024), we tested whether a common false belief that heart rate increases in response to threat lead to an illusory perception of accelerated cardiac activity when anticipating pain. In two experiments, we asked participants to monitor and report their heartbeat while their ECG was recorded. Participants performed these tasks while visual cues reliably predicted a forthcoming harmless (low-intensity) vs. threatening (high-intensity) cutaneous electrical stimulus. We showed that anticipating a painful vs. harmless stimulus causes participants to report an increased cardiac frequency, which does not reflect their real cardiac response, but the common (false) belief that heart rates would accelerate under threat, reflecting the hypothesised integration of prior expectations and interoceptive inputs when estimating cardiac activity.

Here we tested the counterpart of such a cardiac illusion. We reasoned that if cardiac interoception is shaped by expectations about pain, then the inverse should also be true: manipulating beliefs about cardiac activity (via cardiac feedback) in the context of pain anticipation should influence the perception of pain. Specifically, we hypothesized that presenting accelerated cardiac feedback would act as a misleading prior, leading to an illusory increase in pain experience, even in the absence of an actual change in nociceptive input.

Moreover, next to the references already provided in the last version of the manuscript, there is ample prior research that provides more general support for such relationships. Specifically, studies have shown that providing mismatched cardiac feedback in contexts where cardiovascular changes are typically expected (i.e. sexual arousal, Rupp & Wallen, 2008; Valins, 1996; physical exercise, Iodice et al., 2019) can enhance the perception of interoceptive states associated with those experiences. Furthermore, findings that false cardiac feedback can influence emotional experience suggest that it is the conscious perception of physiological arousal, combined with the cognitive interpretation of the stimulus, that plays a key role in shaping emotional responses (Crucian et al., 2000).

This point is now addressed in the revised Introduction, wherein additional references have been integrated (lines 157-170).

Crucian, G. P., Hughes, J. D., Barrett, A. M., Williamson, D. J. G., Bauer, R. M., Bowers, D., & Heilman, K. M. (2000). Emotional and physiological responses to false feedback. Cortex, 36(5), 623-647.

Iodice, P., Porciello, G., Bufalari, I., Barca, L., & Pezzulo, G. (2019). An interoceptive illusion of effort induced by false heart-rate feedback. Proceedings of the National Academy of Sciences, 116(28), 13897-13902.

Parrotta, E., Bach, P., Perrucci, M. G., Costantini, M., & Ferri, F. (2024). Heart is deceitful above all things: Threat expectancy induces the illusory perception of increased heartrate. Cognition, 245, 105719.

Rupp, H. A., & Wallen, K. (2008). Sex differences in response to visual sexual stimuli: A review. Archives of sexual behavior, 37(2), 206-218.

Schoeller, F., Horowitz, A., Maes, P., Jain, A., Reggente, N., Moore, L. C., Trousselard, M., Klein, A., Barca, L., & Pezzulo, G. (2022). Interoceptive technologies for clinical neuroscience.

Tschantz, A., Barca, L., Maisto, D., Buckley, C. L., Seth, A. K., & Pezzulo, G. (2022). Simulating homeostatic, allostatic and goal-directed forms of interoceptive control using active inference. Biological Psychology, 169, 108266.

Valins, S. (1966). Cognitive effects of false heart-rate feedback. Journal of personality and social psychology, 4(4), 400.

(5) The discussion section does not address the study's limitations in a sufficient manner. For example, I would expect a more thorough discussion on the lack of correlation between participant ratings and self-reported bodily awareness and reactivity, as assessed with the BPQ.

We thank the reviewer for this valuable observation. In response, we have revised the Discussion section to explicitly acknowledge and elaborate on the lack of significant correlations between participants’ pain ratings and their self-reported bodily awareness and reactivity as assessed with the BPQ.

We now clarify that the inclusion of this questionnaire was exploratory. While it would be theoretically interesting to observe a relationship between subjective pain modulation and individual differences in interoceptive awareness, detecting robust correlations between within-subject experimental effects and between-subjects trait measures such as the BPQ typically requires much larger sample sizes (often exceeding N = 200) due to the inherently low reliability of such cross-level associations (see Hedge, Powell & Sumner, 2018; the “reliability paradox”). As such, the absence of a significant correlation in our study does not undermine the conclusions we draw from our main findings. Future studies with larger samples will be needed to systematically address this question. We now acknowledge this point explicitly in the revised manuscript (lines 501-504; 832-851).

Hedge, C., Powell, G., & Sumner, P. (2018). The reliability paradox: Why robust cognitive tasks do not produce reliable individual differences. Behavior Research Methods, 50(3), 1166-1186. https://doi.org/10.3758/s13428-017-0935-1

(a) Some short, additional information on why the authors chose to focus on body awareness and supradiaphragmatic reactivity subscales would be helpful.

We chose to focus on the body awareness and supradiaphragmatic reactivity subscales because these aspects are closely tied to emotional and physiological processing, particularly in the context of interoception. Body awareness plays a critical role in how individuals perceive and interpret bodily signals, which in turn affects emotional regulation and self-awareness. Supradiaphragmatic reactivity refers specifically to organs located or occurring above the diaphragm (i.e., the muscle that separates the chest cavity from the abdomen), which includes the heart, compared to subdiaphragmatic reactivity subscales further down. Our decision to include these subscales is further motivated by recent research, including the work by Petzschner et al. (2021), which demonstrates that the focus of attention can modulate the heartbeat-evoked potential (HEP), and that this modulation is predicted by participants’ responses on the supradiaphragmatic reactivity subscales. Thus, this subscale, and the more general body awareness scale, allows us to explore the interplay between bodily awareness, physiological reactivity, and emotional processing in our study. We now clarify this point in the revised version of the Methods - Body Perception Questionnaire (lines 384-393).

(6) The analyses presented in this version of the manuscript allow only limited mechanistic conclusions - a computational model of participants' behavior would be a very strong addition to the paper. While this may be out of the scope of the article, it would be helpful for the reader to discuss the limitations of the presented analyses and outline avenues towards a more mechanistic understanding and analysis of the data. The computational model in [7] might contain some starting ideas.

Thank you for your valuable feedback. We agree that a computational model would enhance the mechanistic understanding of our findings. While this is beyond the current scope, we now discuss the limitations of our analysis in the Limitations and Future directions section (lines 852-863). Specifically, we acknowledge that future studies could use computational models to better understand the interactions between physiological, cognitive, and perceptual factors.

Some additional topics were not considered in the first version of the manuscript:

(1) The possible advantages of a computational model of task behavior should be discussed.

We agree that a computational model of task behavior could provide several advantages. By formalizing principles of predictive processing and active inference, such a model could generate quantitative predictions about how heart rate (HR) and feedback interact, providing a more precise understanding of their respective contributions to pain modulation. However, this is a first demonstration of a theoretically predicted phenomenon, and computationally modelling it is currently outside the scope of the article. We would be excited to explore this in the future. We have added a brief discussion of these potential advantages in the revised manuscript and suggest that future work could integrate computational modelling to further deepen our understanding of these processes (lines 852-890).

(2) Across both experiments, there was a slightly larger number of female participants. Research suggests significant sex-related differences in pain processing [1,2]. It would be interesting to see what role this may have played in this data.

Thank you for your insightful comment. While we acknowledge that sex-related differences in pain processing are well-documented in the literature, we do not have enough participants in our sample to test this in a well-powered way. As such, exploring the role of sex differences in pain perception will need to be addressed in future studies with more balanced samples. It would be interesting if more sensitive individuals, with a more precise representation of pain, also show smaller effects on pain perception. We have noted this point in the revised manuscript (lines 845-851) and suggest that future research could specifically investigate how sex differences might influence the modulation of pain and physiological responses in similar experimental contexts.

(3) There are a few very relevant papers that come to mind which may be of interest. These sources might be particularly useful when discussing the roadmap towards a mechanistic understanding of the inferential processes underlying the task responses [3,4] and their clinical implications.

Thank you for highlighting these relevant papers. We appreciate your suggestion and have now cited them in the Limitations and Future directions paragraph (lines 852-863).

(4) In this version of the paper, we only see plots that illustrate ∆ scores, averaged across pain intensities - to better understand participant responses and the relationship with stimulus intensity, it would be helpful to see a more descriptive plot of task behavior (e.g. stimulus intensity and raw pain ratings)

To directly address the reviewer’s request, we now provide additional descriptive plots in the supplementary material of the revised manuscript, showing raw pain ratings across different stimulus intensities and feedback conditions. These plots offer a clearer view of participant behavior without averaging across pain levels, helping to better illustrate the relationship between stimulus intensity and reported pain.

Mogil, J. S. (2020). Qualitative sex differences in pain processing: emerging evidence of a biased literature. Nature Reviews Neuroscience, 21(7), 353-365. https://www.nature.com/articles/s41583-020-0310-6

Sorge, R. E., & Strath, L. J. (2018). Sex differences in pain responses. Current Opinion in Physiology, 6, 75-81. here

Unal, O., Eren, O. C., Alkan, G., Petzschner, F. H., Yao, Y., & Stephan, K. E. (2021). Inference on homeostatic belief precision. Biological Psychology, 165, 108190.

Allen, M., Levy, A., Parr, T., & Friston, K. J. (2022). In the body's eye: the computational anatomy of interoceptive inference. PLoS Computational Biology, 18(9), e1010490.

Stephan, K. E., Manjaly, Z. M., Mathys, C. D., Weber, L. A., Paliwal, S., Gard, T., ... & Petzschner, F. H. (2016). Allostatic self-efficacy: A metacognitive theory of dyshomeostasis-induced fatigue and depression. Frontiers in human neuroscience, 10, 550.

Friston, K. J., Stephan, K. E., Montague, R., & Dolan, R. J. (2014). Computational psychiatry: the brain as a phantastic organ. The Lancet Psychiatry, 1(2), 148-158.

Eckert, A. L., Pabst, K., & Endres, D. M. (2022). A Bayesian model for chronic pain. Frontiers in Pain Research, 3, 966034.

We thank the reviewer for highlighting these relevant references which have now been integrated in the revised version of the manuscript.

**Recommendations For The Authors:**

**Reviewer #1 (Recommendations For The Authors):**

At the time I was reviewing this paper, I could not think of a detailed experiment that would answer my biggest concern: Is this a manipulation of the brain's interoceptive data integration, or rather a manipulation of participants' alertness which indirectly influences their pain prediction?

One incomplete idea that came to mind was delivering this signal in a more "covert" manner (though I am not sure it will suffice), or perhaps correlating the effect size of a participant with their interoceptive abilities, as measured in a different task or through a questionnaire.... Another potential idea is to tell participants that this is someone else's HR that they hear and see if that changes the results (though requires further thought). I leave it to the authors to think further, and perhaps this is to be answered in a different paper - but if so, I am sorry to say that I do not think the claims can remain as they are now, and the paper will need a revision of its arguments, unfortunately. I urge the authors to ask further questions if my point about the concern was not made clear enough for them to address or contemplate it.

We thank the reviewer for raising this important point. As detailed in our previous response, this point invites an important clarification regarding the role of cardiac deceleration in threat processing. Rather than serving as an interoceptive input from which the brain infers the likelihood of a forthcoming aversive event, heart rate deceleration is better described as an output of an already ongoing predictive process, as it reflects an allostatic adjustment of the bodily state aimed at minimizing the impact of the predicted perturbation (e.g., pain) and preventing sympathetic overshoot. It would be maladaptive for the brain to use a decelerating heart rate as evidence of impending threat, since this would paradoxically trigger further parasympathetic activation, initiating a potentially destabilizing feedback loop. Conversely, increased heart rate represents an evolutionarily conserved cue for arousal, threat, and pain. Our results therefore align with the idea that the brain treats externally manipulated increases in cardiac signals as congruent with anticipated sympathetic activation, prompting a compensatory autonomic and perceptual response consistent with embodied predictive processing frameworks (e.g., Barrett & Simmons, 2015; Seth, 2013).

We would also like to re-iterate that our results cannot be explained by general differences induced by the different heart rate sounds relative to the exteroceptive (see also our detailed comments to your point above, and our response to a similar point from Reviewer 3), for three main reasons.

(1) No main effect of Experiment on pain ratings:

If the cardiac feedback had simply increased arousal or attention in a general (non-specific) way, we would expect a main effect of Experiment (i.e., interoceptive vs exteroceptive condition) on pain intensity or unpleasantness ratings, regardless of feedback frequency. However, such a main effect was never observed. Instead, effects were specific to the manipulation of feedback frequency.

(2) Heart rate as an arousal measure:

Heart rate (HR) is a classical physiological index of arousal. If there had been an unspecific increase in arousal in the interoceptive condition, we would expect a main effect of Experiment on HR. However, no such main effect was found. Instead, our HR analyses revealed a significant interaction between feedback and experiment, suggesting that HR changes depended specifically on the feedback manipulation rather than reflecting a general arousal increase.

(3) Arousal predicts faster, not slower, heart rates

In Experiment 1, faster interoceptive cardiac feedback led to a slowdown in heartrates both when compared to slower feedback and to congruent cardiac feedback. This is in line with the predicted compensatory response to faster heart rates. In contrast, if faster feedback would have only generally increased arousal, heart rates should have increased instead of decreased, as indicated by several prior studies (for a review, see Forte et al., 2022), predicting the opposite pattern of responses than was found in Experiment 1.

Taken together, these findings indicate that the effects observed are unlikely to be driven by unspecific arousal or attention mechanisms, but rather are consistent with feedback-specific modulations, in line with our interoceptive inference framework. We now integrate these considerations in the general discussion (lines 796-830).

Barrett, L. F., & Simmons, W. K. (2015). Interoceptive predictions in the brain. Nature reviews neuroscience, 16(7), 419-429.

Forte, G., Troisi, G., Pazzaglia, M., Pascalis, V. D., & Casagrande, M. (2022). Heart rate variability and pain: a systematic review. Brain sciences, 12(2), 153.

Seth, A. K. (2013). Interoceptive inference, emotion, and the embodied self. Trends in Cognitive Sciences, 17(11), 565-573.

Additional recommendations:

Major (in order of importance):

(1) Number of trials per participant, per condition: as I mentioned, having only 6 trials for each condition is very little. The minimum requirement to accept so few trials would be to show data about the distribution of participants' responses to these trials, both per pain intensity (which was later averaged across - another issue discussed later), and across pain intensities, and see that it allows averaging across and that it is not incredibly variable such that the mean is unreliable.

We appreciate the reviewer’s concern regarding the limited number of trials per condition. This choice was driven by both theoretical and methodological considerations.

First, as is common in body illusion paradigms (e.g., the Rubber Hand Illusion, Botvinick & Cohen, 1998; the Full Body Illusion, Ehrsson, 2007; the Cardio-visual full body illusion, Pratviel et al., 2022) only a few trials are typically employed due to the immediate effects these manipulations elicit. Repetition can reduce the strength of the illusion through habituation, increased awareness, or loss of believability.

Second, the experiment was already quite long (1.5h to 2h per participant) and cognitively demanding. It would not have been feasible to expand it further without compromising data quality due to fatigue, attentional decline, or participant disengagement.

Third, the need for a large number of trials is more relevant when using implicit measures such as response times or physiological indices, which are typically indirectly related to the psychological constructs of interest. In contrast, explicit ratings are often more sensitive and less noisy, and thus require fewer repetitions to yield reliable effects (e.g., Corneille et al., 2024).

Importantly, we also addressed your concern analytically. We ran therefore linear mixed-effects model analyses across all dependent variables (See Supplementary materials), with Trial (i.e., the rank order of each trial) included as a predictor to account for potential time-on-task effects such as learning, adaptation, or fatigue (e.g., Möckel et al., 2015). These models captured trial-by-trial variability and allowed us to test for systematic changes in heart rate (HR) and pain ratings including interactions with feedback conditions (e.g., Klieg et al., 2011; Baayen et al., 2010; Ambrosini et al., 2019). The consistent effects of Trial suggest that repetition dampens the illusion, reinforcing our decision to limit the number of exposures.

In the interoceptive experiment, these analyses revealed a significant Feedback × Trial interaction (F(3, 711.19) = 6.16, p < .001), indicating that the effect of feedback on HR was not constant over time. As we suspected, and in line with other illusion-like effects, the difference between Faster and Slower feedback, which was significant early on (estimate = 1.68 bpm, p = .0007), decreased by mid-session (estimate = 0.69 bpm, p = .0048), and was no longer significant in later trials (estimate = 0.30 bpm, p = .4775). At the end of the session, HR values in the Faster and Slower conditions even numerically converged (Faster: M = 74.4, Slower: M = 74.1), and the non-significant contrast confirms that the difference had effectively vanished (for further details about slope estimation, see Supplementary material).

The same pattern emerged for pain-unpleasantness ratings. A significant Feedback × Trial interaction (F (3, 675.33) = 3.44, p = .0165) revealed that the difference between Faster and Slower feedback was strongest at the beginning of the session and progressively weakened. Specifically, Faster feedback produced higher unpleasantness than Slower in early trials (estimate = -0.28, p = .0058) and mid-session (estimate = - 0.19, p = .0001), but this contrast was no longer significant in the final trials, wherein all the differences between active feedback conditions vanished (all ps > .55).

Finally, similar results were yielded for pain intensity ratings. A significant Feedback × Trial interaction (F (3, 669.15) = 9.86, p < .001) showed that the Faster vs Slower difference was greatest at the start of the session and progressively vanished over trials. In early trials Faster feedback exceeded Slower (estimate=-8.33, p = .0001); by mid-session this gap had shrunk to 4.48 points (p < .0001); and in the final trials it was no longer significant (all ps > .94).

Taken together, our results show that the illusion induced by Faster relative to slower feedback fades with repetition; adding further trials would likely have masked this key effect, confirming the methodological choice to restrict each condition to fewer exposures. To conclude, given that this is the first study to investigate an illusion of pain using heartbeat-based manipulation, we intentionally limited repeated exposures to preserve the integrity of the illusion. The use of mixed models as complementary analyses strengthens the reliability of our conclusions within these necessary design constraints. We now clarify this point in the Procedure paragraph (lines 328-335)

Ambrosini, E., Peressotti, F., Gennari, M., Benavides-Varela, S., & Montefinese, M. (2023). Aging-related effects on the controlled retrieval of semantic information. Psychology and Aging, 38(3), 219.

Baayen, R. H., & Milin, P. (2010). Analyzing reaction times. International Journal of Psychological Research, 3(2), 12-28.

Botvinick, M., & Cohen, J. (1998). Rubber hands ‘feel’touch that eyes see. Nature, 391(6669), 756-756.

Corneille, O., & Gawronski, B. (2024). Self-reports are better measurement instruments than implicit measures. Nature Reviews Psychology, 3(12), 835–846.

Ehrsson, H. H. (2007). The experimental induction of out-of-body experiences. Science, 317(5841), 1048-1048.

Kliegl, R., Wei, P., Dambacher, M., Yan, M., & Zhou, X. (2011). Experimental effects and individual differences in linear mixed models: Estimating the relation of spatial, object, and attraction effects in visual attention. Frontiers in Psychology, 1, 238. https://doi.org/10.3389/fpsyg.2010.00238

Möckel, T., Beste, C., & Wascher, E. (2015). The effects of time on task in response selection-an ERP study of mental fatigue. Scientific reports, 5(1), 10113.

Pratviel, Y., Bouni, A., Deschodt-Arsac, V., Larrue, F., & Arsac, L. M. (2022). Avatar embodiment in VR: Are there individual susceptibilities to visuo-tactile or cardio-visual stimulations?. Frontiers in Virtual Reality, 3, 954808.

(2) Using different pain intensities: what was the purpose of training participants on correctly identifying pain intensities? You state that the aim of having 5 intensities is to cause ambiguity. What is the purpose of making sure participants accurately identify the intensities? Also, why then only 3 intensities were used in the test phase? The rationale for these is lacking.

We thank the reviewer for raising these important points regarding the use of different pain intensities. The purpose of using five levels during the calibration and training phases was to introduce variability and increase ambiguity in the participants’ sensory experience. This variability aimed to reduce predictability and prevent participants from forming fixed expectations about stimulus intensity, thereby enhancing the plausibility of the illusion. It also helped prevent habituation to a single intensity and made the manipulation subtler and more credible. We had no specific theoretical hypotheses about this manipulation. Regarding the accuracy training, although the paradigm introduced ambiguity, it was important to ensure that participants developed a stable and consistent internal representation of the pain scale. This step was essential to control for individual differences in sensory discrimination and to ensure that illusion effects were not confounded by participants’ inability to reliably distinguish between intensities.

As for the use of only three pain intensities in the test phase, the rationale was to focus on a manageable subset that still covered a meaningful range of the stimulus spectrum. This approach followed the same logic as Iodice et al. (2019, PNAS), who used five (rather than all seven) intensity levels during their experimental session. Specifically, they excluded the extreme levels (45 W and 125 W) used during baseline, to avoid floor and ceiling effects and to ensure that each test intensity could be paired with both a “slower” and a “faster” feedback from an adjacent level. This would not have been possible at the extremes of the intensity range, where no adjacent level exists in one direction. We adopted the same strategy to preserve the internal consistency and plausibility of our feedback manipulation.

We further clarified these points in the revised manuscript (lines 336-342).

Iodice, P., Porciello, G., Bufalari, I., Barca, L., & Pezzulo, G. (2019). An interoceptive illusion of effort induced by false heart-rate feedback. Proceedings of the National Academy of Sciences, 116(28), 13897-13902.

(3) Averaging across pain intensities: this is, in my opinion, not the best approach as by matching a participant's specific responses to a pain stimulus before and after the manipulation, you can more closely identify changes resulting from the manipulation. Nevertheless, the minimal requirement to do so is to show data of distributions of pain intensities so we know they did not differ between conditions per participant, and in general - as you indicate they were randomly distributed.

We thank the reviewer for this thoughtful comment. The decision to average across pain intensities in our main analyses was driven by the specific aim of the study: we did not intend to determine at which exact intensity level the illusion was most effective, and the limited number of trials makes such an analysis difficult. Rather, we introduced variability in nociceptive input to increase ambiguity and reduce predictability in the participants’ sensory experience. This variability was critical for enhancing the plausibility of the illusion by preventing participants from forming fixed expectations about stimulus strength. Additionally, using a range of intensities helped to minimize habituation effects and made the feedback manipulation subtler and more credible.

That said, we appreciate the reviewer’s point that matching specific responses before and after the manipulation at each intensity level could provide further insights into how the illusion operates across varying levels of nociceptive input. We therefore conducted supplementary analyses using linear mixed-effects models in which all three stimulus intensities were included as a continuous fixed factor. This allowed us to examine whether the effects of feedback were intensity-specific or generalized across different levels of stimulation

These analyses revealed that, in both the interoceptive and exteroceptive experiments, the effect of feedback on pain ratings was significantly modulated by stimulus intensity, as indicated by a Feedback × Stimulus Intensity interaction (Interoceptive: unpleasantness F(3, 672.32)=3.90, p=.0088; intensity ratings F(3, 667.07)=3.46, p=.016. Exteroceptive: unpleasantness F(3, 569.16)=8.21, p<.0001; intensity ratings F(3, 570.65)=3.00, p=.0301). The interaction term confirmed that the impact of feedback varied with stimulus strength, yet the pattern that emerged in each study diverged markedly.

In the interoceptive experiment, the accelerated-heartbeat feedback (Faster) systematically heightened pain relative to the decelerated version (Slower) at every level of noxious input: for low-intensity trials Faster exceeded Slower by 0.22 ± 0.08 points on the unpleasantness scale (t = 2.84, p = .0094) and by 3.87 ± 1.69 units on the numeric intensity scale (t = 2.29, p = .0448); at the medium intensity the corresponding differences were 0.19 ± 0.05 (t = -4.02, p = .0001) and 4.52 ± 1.06 (t = 4.28, p < .0001); and even at the highest intensity, Faster still surpassed Slower by 0.17 ± 0.08 on unpleasantness (t = 2.21, p = .0326) and by 5.16 ± 1.67 on intensity (t = 3.09, p = .0032). This uniform Faster > Slower pattern indicates that the interoceptive manipulation amplifies perceived pain in a stimulus-independent fashion.

The exteroceptive control experiment told a different story: the Faster-Slower contrast reached significance only at the most noxious setting (unpleasantness: estimate = 0.24 ± 0.07, t = -3.24, p = .0019; intensity: estimate = - 5.14 ± 1.82, t = 2.83, p = .0072) and was absent at the medium level (intensity , p=0.29; unpleasantness, p=0.45), while at the lowest level Slower actually produced numerically higher unpleasantness (2.56 versus 2.40) and intensity ratings (44.7 versus 42.2).

Thus, although both studies show that feedback effects depend on the actual nociceptive level of the stimulus, the results suggest that the faster vs. slower interoceptive feedback manipulation delivers a robust and intensity-invariant enhancement of pain, whereas the exteroceptive cue exerts a sporadic influence that surfaces solely under maximal stimulation.

These new results are now included in the Supplementary Materials, where we report the detailed analyses for both the Interoceptive and Exteroceptive experiments on the Likert unpleasantness ratings and the numeric pain intensity ratings.

(4) Sample size: It seems that the sample size was determined after the experiment was conducted, as the required N is identical to the actual N. I would be transparent about that, and say that retrospective sample size analyses support the ability of your sample size to support your claims. In general, a larger sample size than is required is always recommended, and if you were to run another study, I suggest you increase the sample size.

As also addressed in our responses to your later comments (see our detailed reply regarding the justification of SESOI and power analyses), the power analyses reported here were not post-hoc power analyses based on obtained results. In line with current recommendations (Anderson, Kelley & Maxwell, 2017; Albers & Lakens, 2018), we did not base our analyses on previously reported effect sizes, as these can carry considerable uncertainty, particularly for novel effects where robust estimates are lacking. Instead, we used sensitivity analyses, conducted using the sensitivity analysis function in G*Power (Version 3.1). Sensitivity analyses allow us to report effect sizes that our design was adequately powered (90%) to detect, given the actual sample size, desired power level, and the statistical test used in each experiment (Lakens, 2022). Following further guidance (Lakens, 2022), we also report the smallest effect size of interest (SESOI) that these tests could reliably detect.

This approach indicated that our design was powered to detect effect sizes of d = 0.57 in Experiment 1 and d = 0.62 in Experiment 2, with corresponding SESOIs of d = 0.34 and d = 0.37, respectively. The slightly higher value in Experiment 2 reflects the greater number of participants excluded (from an equal number originally tested) based on pre-specified criteria. Importantly, both experiments were well-powered to detect effects smaller than those typically reported in similar top-down pain modulation studies, where effect sizes around d = 0.7 have been observed (Iodice et al., 2019).

We have now clarified this rationale in the revised manuscript, Experiment 1- Methods - Participants (lines 208-217).

Albers, C., & Lakens, D. (2018). When power analyses based on pilot data are biased: Inaccurate effect size estimators and follow-up bias. Journal of experimental social psychology, 74, 187-195.

Anderson, S. F., Kelley, K., & Maxwell, S. E. (2017). Sample-Size Planning for More Accurate Statistical Power: A Method Adjusting Sample Effect Sizes for Publication Bias and Uncertainty. Psychological Science, 28(11), 1547-1562. https://doi.org/10.1177/0956797617723724

Lakens, D. (2022). Sample size justification. Collabra: psychology, 8(1), 33267.

(5) Analysis: the use of change scores instead of the actual scores is not recommended, as it is a loss of data, but could have been ignored if it didn't have a significant effect on the analyses conducted. Instead of conducting an RM-ANOVA of conditions (faster, slower, normal heartbeats) across participants, finding significant interaction, and then moving on to specific post-hoc paired comparisons between conditions, the authors begin with the change score but then move on to conduct the said paired comparisons without ever anchoring these analyses in an appropriate larger ANOVA. I strongly recommend the use of an ANOVA but if not, the authors would have to correct for multiple comparisons at the minimum.

We thank the reviewer for their comment regarding the use of change scores. These were originally derived from the difference between the slower and faster feedback conditions relative to the congruent condition. In line with the reviewer’s recommendation, we have now removed these difference-based change scores from the main analysis. The results remain identical. Please note that we have retained the normalization procedure, relative to each participant’s initial baseline in the no feedback trials, as it is widely used in the interoceptive and pain literature (e.g., Bartolo et al., 2013; Cecchini et al., 2020; Riello et al., 2019). This approach helps to control for interindividual variability and baseline differences by expressing each participant’s response relative to their no-feedback baseline. As before, normalization was applied across all dependent variables (heart rate, pain intensity, and pain unpleasantness).

To address the reviewer’s concern about statistical validity, we now first report a 1-factor repeated-measures ANOVA (Greenhouse-Geisser corrected) for each dependent variable, with feedback condition (slower, congruent, faster) as the within-subject factor.

These show in each case a significant main effect, which we then follow with planned paired-sample t-tests comparing:

Faster vs. slower feedback (our main hypothesis, as these manipulations are expected to produce largest, most powerful, test of our hypothesis, see response to Reviewer 3),

Faster vs. congruent and slower vs. congruent (to test for potential asymmetries, as suggested by previous false heart rate feedback studies).

The rationale of these analyses is further discussed in the Data Analysis of Experiment 1 (lines 405-437).

Although we report the omnibus one-factor RM-ANOVAs to satisfy conventional expectations, we note that such tests are not statistically necessary, nor even optimal, when the research question is fully captured by a priori, theory-driven contrasts. Extensive methodological work shows that, in this situation, going straight to planned contrasts maximises power without inflating Type I error and avoids the logical circularity of first testing an effect one does not predict (e.g., Rosenthal & Rosnow, 1985). In other words, an omnibus F is warranted only when one wishes to protect against unspecified patterns of differences. Here our hypotheses were precise (Faster ≠ Slower; potential asymmetry relative to Congruent), so the planned paired comparisons would have sufficed statistically. We therefore include the RM-ANOVAs solely for readers who expect to see them, but our inferential conclusions rest on the theoretically motivated contrasts.

Rosenthal, R., & Rosnow, R. L. (1985). Contrast analysis. New York: Cambridge.

(6) Correlations: were there correlations between subjects' own heartbeats (which are considered a predictive cue) and pain perceptions? This is critical to show that the two are in fact related.

We thank the reviewer for this thoughtful suggestion. While we agree that testing for a correlation between anticipatory heart rate responses and subjective pain ratings is theoretically relevant. However, we have not conducted this analysis in the current manuscript, as our study was not designed or powered to reliably detect such individual differences. As noted by Hedge, Powell, and Sumner (2018), robust within-subject experimental designs tend to minimize between-subject variability in order to detect clear experimental effects. This reduction in variance at the between-subject level limits the reliability of correlational analyses involving trait-like or individual response patterns. This issue, known as the reliability paradox, highlights that measures showing robust within-subject effects may not show stable individual differences, and therefore correlations with other individual-level variables (like subjective ratings used here) require much larger samples to produce interpretable results than available here (and commonly used in the literature), typically more than 200 participants. For these reasons, we believe that running such an analysis in our current dataset would not yield informative results and could be misleading.

We now explicitly acknowledge this point in the revised version of the manuscript (Limitations and future directions, lines 832-851) and suggest that future studies specifically designed to examine individual variability in anticipatory physiological responses and pain perception would be better suited to address this question.

Hedge, C., Powell, G., & Sumner, P. (2018). The reliability paradox: Why robust cognitive tasks do not produce reliable individual differences. Behavior Research Methods, 50(3), 1166-1186. https://doi.org/10.3758/s13428-017-0935-1

(7) The direct comparison between studies is great! and finally the use of ANOVA - but why without the appropriate post-hoc tests to support the bold claims in lines 542-544? This is needed. Same for 556-558.

We apologize if our writing was not clear here, but the result of the ANOVAs fully warrants the claims in 542-544 (now lines 616-618) and 556-558 (now lines 601-603).

In a 2x2 design, the interaction term is mathematically identical to comparing the difference induced by Factor 1 at one level of Factor 2 with the same difference induced at the other level of Factor 2. In our 2x2 analysis with the factors Experiment (Cardiac feedback, Exteroceptive feedback - between participants) and Feedback Frequency (faster, slower - within participants), the interaction therefore directly tests whether the effect of Feedback frequency differs statistically (i.e., is larger or smaller) in the participants in the interoceptive and exteroceptive experiments. Thus, the conclusion that “faster feedback affected the perceptual bias more strongly in the Experiment 1 than in Experiment 2” captures the outcome of the significant interaction exactly. Indeed, this test would be statistically equivalent (and would produce identical p values) to a simple between-group t-test between each participant’s difference between the faster and slower feedback in the interoceptive group and the analogous differences between the faster and slower feedback in the exteroceptive group, as illustrated in standard examples of factorial analysis (see, e.g., Maxwell, Delaney and Kelley, 2018).

Please note that, for the above reason, mathematically the conclusion of larger effects in one experiment than the other is licensed by the significant interaction even without follow-up t-tests. However, if the reader would like to see these tests, they are simply the main analysis results reported in each of the two experiment sections, where significant (t-test) differences between faster and slower feedback were induced with interoceptive cues (Experiment 1) but not exteroceptive cues (Experiment 2). Reporting them in the between-experiment comparison section again would therefore be redundant.

To avoid this lack of clarity, we have now re-written the results section of each experiment. First, as noted above, we now precede our main hypothesis test - the crucial t-test comparing heartrate and pain ratings after faster vs slower feedback - with an ANOVA including all three levels (faster, congruent, slower feedback). Moreover, we removed the separate between-experiment comparison section. Instead, in the Result section of the exteroceptive Experiment 2, we now directly compare the (absent or reversed) effects of faster vs slower feedback directly, with a between-groups t-test, with the present effects in the interoceptive Experiment 1. This shows conclusively, and hopefully more clearly, that the effects in both experiments differ. We hope that this makes the logic of our analyses clearer.

Maxwell, S. E., Delaney, H. D., & Kelley, K. (2017). Designing experiments and analyzing data: A model comparison perspective. Routledge.

(8) The discussion is missing a limitation paragraph.

Thank you for the suggestion. We have now added a dedicated limitations paragraph in the Discussion section (lines 832-890).

Additional recommendations:

Minor (chronological order):

(1) Sample size calculations for both experiments: what was the effect size based on? A citation or further information is needed. Also, clarify why the effect size differed between the two experiments.

Please see above

(2) "Participants were asked to either not drink coffee or smoke cigarettes" - either is implying that one of the two was asked. I suspect it is redundant as both were not permitted.

The intention was to restrict both behaviors, so we have corrected the sentence to clarify that participants were asked not to drink coffee or smoke cigarettes before the session.

(3) Normalization of ECG - what exactly was normalized, namely what measure of the ECG?

The normalized measure was the heart rate, expressed in beats per minute (bpm). We now clarify this in the Data Analysis section of Experiment 1 (Measures of the heart rate recorded with the ECG (beats per minute) in the feedback phase were normalized)

(4) Line 360: "Mean Δ pain unpleasantness ratings were analysed analogously" - this is unclear, if already described in methods then should be removed here, if not - should be further explained here.

Thank you for your observation. We are no longer using change scores.

(5) Lines 418-420: "Consequently, perceptual and cardiac modulations associated with the feedback manipulation should be reduced over the exposure to the faster exteroceptive sound." - why reduced and not unchanged? I didn't follow the logic.

We chose the term “reduced” rather than “unchanged” to remain cautious in our interpretation. Statistically, the absence of a significant effect in one experiment does not necessarily mean that no effect is present; it simply means we did not detect one. For this reason, we avoided using language that would suggest complete absence of modulation. It also more closely matches the results of the between experiment comparisons that we report in the Result section of Experiment 2, which can in principle only show that the effect in Experiment 2 was smaller than that of Experiment 1, not that it was absent. Even the TOST analysis that we utilize to show the absence of an effect can only show that any effect that is present is smaller than we could reasonably expect to detect with our experimental design, not its complete absence.

Also, on a theoretical level, pain is a complex, multidimensional experience influenced not only by sensory input but also by cognitive, emotional, social and expectancy factors. For this reason, we considered it important to remain open to the possibility that other mechanisms beyond the misleading cardiac prior induced by the feedback might have contributed to the observed effects. If such other influences had contributed to the induced differences between faster and slower feedback in Experiment 1, some remainder of this difference could have been observed in Experiment 2 as well.

Thus, for both statistical and theoretical reasons, we were careful to predict a reduction of the crucial difference, not its complete elimination. However, to warrant the possibility that effects could be completely eliminated we now write that “perceptual and cardiac modulations associated with the feedback manipulation should be reduced or eliminated with exteroceptive feedback”

(6) Study 2 generation of feedback - was this again tailored per participants (25% above and beyond their own HR at baseline + gradually increasing or decreasing), or identical for everyone?

Yes, in Study 2, the generation of feedback was tailored to each participant, mirroring the procedure or Experiment 1. Specifically, the feedback was set to be 25% above or below their baseline heart rate, with the feedback gradually increasing or decreasing. This individualized approach ensured that each participant experienced feedback relative to their own baseline heart rate. We now clarify this in the Methods section (lines 306-318).

(7) I did not follow why we need the TOST and how to interpret its results.

We thank the reviewer for raising this important point. In classical null hypothesis significance testing (NHST), a non-significant p-value (e.g., p > .05) only indicates that we failed to find a statistically significant difference, not that there is no difference. It therefore does not allow us to conclude that two conditions are equivalent – only that we cannot confidently say they are different. In our case, to support the claim that exteroceptive feedback does not induce perceptual or physiological changes (unlike interoceptive feedback), we needed a method to test for the absence of a meaningful effect, not just the absence of a statistically detectable one.

The TOST (Two One-Sided Tests) procedure reverses the logic of NHST by testing whether the observed effect falls within a predefined equivalence interval, called the smallest effect size of interest (SESOI) that is in principle measurable with our design parameters (e.g., type of test, number of participants). This approach is necessary when the goal is not to detect a difference, but rather to demonstrate that an observed effect is so small that it can be considered negligible – or at the least smaller than we could in principle expect to observe in the given experiment. We used the TOST procedure in Experiment 2 to test for statistical equivalence between the effects of faster and slower exteroceptive feedback on pain ratings and heart rate.

We hope that the clearer explanation now provided in data analysis of Experiment 2 section (lines 5589-563) fully addresses the reviewer’s concern.

(8) Lines 492-3: authors say TOST significant, while p value = 0.065

We thank the reviewer for spotting this inconsistency. The discrepancy was due to a typographical error in the initial manuscript. During the revision of the paper, we rechecked and fully recomputed all TOST analyses, and the results have now been corrected throughout the manuscript to accurately reflect the statistical outcomes. In particular, for the comparison of heart rate between faster and slower exteroceptive feedback in Experiment 2, the corrected TOST analysis now shows a significant equivalence, with the observed effect size being d = -0.19 (90% CI [-0.36, -0.03]) and both one-sided tests yielding p = .025 and p < .001. These updated results are reported in the revised Results section.

**Reviewer #2 (Recommendations For The Authors):**

I would suggest the authors revise their definition of pain in the introduction, since it is not always a protective experience. The new IASP definition specifically takes this into consideration.

We thank the reviewer for this suggestion. We have updated the definition of pain in the Introduction (lines 2-4) to align with the most recent IASP definition (2020), which characterizes pain as “an unpleasant sensory and emotional experience associated with, or resembling that associated with, actual or potential tissue damage” (lines 51-53).

The work on exteroceptive cues does not necessarily neglect the role of interoceptive sources of information, although it is true that it has been comparatively less studied. I suggest rephrasing this sentence to reflect this.

We thank the reviewer for pointing out this important nuance. We agree that studies employing exteroceptive cues to modulate pain perception do not necessarily neglect the role of interoceptive sources, even though these are not always the primary focus of investigation. Our intention was not to imply a strict dichotomy, but rather to highlight that interoceptive mechanisms have been comparatively under-investigated. We have revised the sentence in the Introduction accordingly to better reflect this perspective (Introduction, lines 110-112, “Although interoceptive processes may have contributed to the observed effects, these studies did not specifically target interoceptive sources of information within the inferential process.”).

The last paragraph of the introduction (lines 158-164) contains generalizations beyond what can be supported by the data and the results, about the generation of predictive processes and the origins of these predictions. The statements regarding the understanding of pain-related pathologies in terms of chronic aberrant predictions in the context of this study are also unwarranted.

We have deleted this paragraph now.

I could not find the study registration (at least in clinicaltrials.gov). This is curious considering that the hypothesis and the experimental design seem in principle well thought out, and a study pre-registration improves the credibility of the research (Nosek et al., 2018). I also find the choice for the smallest effect of interest (SESOI) odd. Besides the unnecessary variable transformations (more on that later), there is no justification for why that particular SESOI was chosen, or why it changes between experiments (Dienes, 2021; King, 2011), which makes the choice look arbitrary. The SESOI is a fundamental component of a priori power analysis (Lakens, 2022), and without rationale and preregistration, it is impossible to tell whether this is a case of SPARKing or not (Sasaki & Yamada, 2023).

We acknowledge that the study was not preregistered. Although our hypotheses and design were developed a priori and informed by established theoretical frameworks, the lack of formal preregistration is a limitation.

The SESOI values for Experiments 1 and 2 were derived from sensitivity analyses based on the fixed design parameters (type of test, number of participants, alpha level) of our study, not from any post-hoc interpretation based on observed results - they can therefore not be a case of SPARKing. Following current recommendations (Anderson, Kelley & Maxwell, 2017; Albers & Lakens, 2017; Lakens, 2022), we avoided basing power estimates on published effect sizes, as no such values exist for in novel paradigms, and are typically inflated due to publication and other biases. Instead, sensitivity analyses (using G*Power, v 3.1) allows us to calculate, prospectively, the smallest effect each design could detect with 90 % power, given the actual sample size, test type, and α level. Because more participants were excluded in Experiment 2, this design can detect slightly larger effects (d = 0.62) than Experiment 1 (d = 0.57). Please note that both studies therefore remain well-powered to capture effects of the magnitude typically reported in previous research using feedback manipulations to explore interoceptive illusions (e.g., Iodice et al., 2019, d ≈ 0.7).

We have added this clarification to the Participants section of Experiment 1 (Lines 208-217).

Anderson, S. F., Kelley, K., & Maxwell, S. E. (2017). Sample-Size Planning for More Accurate Statistical Power: A Method Adjusting Sample Effect Sizes for Publication Bias and Uncertainty. Psychological Science, 28(11), 1547-1562.

Lakens, D. (2022). Sample size justification. Collabra: psychology, 8(1), 33267.

Albers, C., & Lakens, D. (2018). When power analyses based on pilot data are biased: Inaccurate effect size estimators and follow-up bias. Journal of experimental social psychology, 74, 187-195.

In the Apparatus subsection, it is stated that the intensity of the electrical stimuli was fixed at 2 ms. I believe the authors refer to the duration of the stimulus, not its intensity.

You are right, thank you for pointing that out. The text should refer to the duration of the electrical stimulus, not its intensity. We have corrected this wording in the revised manuscript to avoid confusion.

It would be interesting to report (in graphical form) the stimulation intensities corresponding to the calibration procedure for the five different pain levels identified for all subjects.

That's a good suggestion. We have included a supplementary figure showing the stimulation intensities corresponding to the five individually calibrated pain levels across all participants (Supplementary Figure 11.)

It is questionable that researchers state that "pain and unpleasantness should be rated independently" but then the first level of the Likert scale for unpleasantness is "1=no pain". This is particularly relevant since simulation (and specifically electrical stimulation) can be unpleasant but non-painful at the same time. Since the experiments were already performed, the researchers should at least explain this choice.

Thank you for raising this point. You are right in that the label of “no pain” in the pain unpleasantness scale was not ideal, and we now acknowledge this in the text (lines 886-890). Please note that this was always the second rating that participants gave (after pain intensity), and the strongest results come from this first rating.

Discussion.

I did not find in the manuscript the rationale for varying the frequency of the heart rate by 25% (instead of any other arbitrary quantity).

We thank the Reviewer for this observation, which prompted us to clarify the rationale behind our choice of a ±25% manipulation of heart rate feedback. False feedback paradigms have historically relied on a variety of approaches to modulate perceived cardiac signals. Some studies have adopted non-individualised values, using fixed frequencies (e.g., 60 or 110 bpm) to evoke states of calm or arousal, independently of participants’ actual physiology (Valins, 1966; Shahidi & Baluch, 1991; Crucian et al., 2000; Tajadura-Jiménez et al., 2008). Others have used the participant’s real-time heart rate as a basis, introducing accelerations or decelerations without applying a specific percentage transformation (e.g., Iodice et al., 2019). More recently, a growing body of work has employed percentage-based alterations of the instantaneous heart rate, offering a controlled and participant-specific manipulation. These include studies using −20% (Azevedo et al., 2017), ±30% (Dey et al., 2018), and even ±50% (Gray et al., 2007).

These different methodologies - non-individualised, absolute, or proportionally scaled - have all been shown to effectively modulate subjective and physiological responses. They suggest that the impact of false feedback does not depend on a single fixed method, but rather on the plausibility and salience of the manipulation within the context of the task. We chose to apply a ±25% variation because it falls well within the most commonly used range and strikes a balance between producing a detectable effect and maintaining the illusion of physiological realism. The magnitude is conceptually justified as being large enough to shape interoceptive and emotional experience (as shown by Azevedo and Dey), yet small enough to avoid implausible or disruptive alterations, such as those approaching ±50%. We have now clarified this rationale in the revised Procedure paragraph of Experiment 1 (lines 306-318).

T. Azevedo, R., Bennett, N., Bilicki, A., Hooper, J., Markopoulou, F., & Tsakiris, M. (2017). The calming effect of a new wearable device during the anticipation of public speech. Scientific reports, 7(1), 2285.

Crucian, G. P., Hughes, J. D., Barrett, A. M., Williamson, D. J. G., Bauer, R. M., Bowers, D., & Heilman, K. M. (2000). Emotional and physiological responses to false feedback. Cortex, 36(5), 623-647.

Dey, A., Chen, H., Billinghurst, M., & Lindeman, R. W. (2018, October). Effects of manipulating physiological feedback in immersive virtual environments. In Proceedings of the 2018 Annual Symposium on Computer-Human Interaction in Play (pp. 101-111).

Gray, M. A., Harrison, N. A., Wiens, S., & Critchley, H. D. (2007). Modulation of emotional appraisal by false physiological feedback during fMRI. PLoS one, 2(6), e546.

Shahidi, S., & Baluch, B. (1991). False heart-rate feedback, social anxiety and self-attribution of embarrassment. Psychological reports, 69(3), 1024-1026.

Tajadura-Jiménez, A., Väljamäe, A., & Västfjäll, D. (2008). Self-representation in mediated environments: the experience of emotions modulated by auditory-vibrotactile heartbeat. CyberPsychology & Behavior, 11(1), 33-38.

Valins, S. (1966). Cognitive effects of false heart-rate feedback. Journal of personality and social psychology, 4(4), 400.

The researchers state that pain ratings collected in the feedback phase were normalized to the no-feedback phase to control for inter-individual variability in pain perception, as established by previous research. They cite three studies involving smell and taste, of which the last two contain the same normalization presented in this study. However, unlike these studies, the outcomes here require no normalization whatsoever, because there should be no (or very little) inter-individual variability in pain intensity ratings. Indeed, pain intensity ratings in this study are anchored to 30, 50, and 70 / 100 as a condition of the experimental design. The researchers go to extreme lengths to ensure this is the case, by adjusting stimulation intensities until at least 75% of stimulation intensities are correctly matched to their pain ratings counterpart in the pre-experiment procedure. In other words, inter-individual variability in this study is in stimulation intensities, and not pain intensity ratings. Even if it could be argued that pain unpleasantness and heart rate still need to account for inter-individual variability, the best way to do this is by using the baseline (no-feedback) measures as covariates in a mixed linear model. Another advantage of this approach is that all the effects can be described in terms of the original scales and are readily understandable, and post hoc tests between levels can be corrected for multiple comparisons. On the contrary, the familywise error rate for the comparisons between conditions in the current analysis is larger than 5% (since there is a "main" paired t-test and additional "simple" tests).

We disagree that there is little to no variability in the no feedback phase. Participants were tested in their ability to distinguish intensities in an initial pre-experiment calibration phase. In the no feedback phase, participants rated the pain stimuli in the full experimental context.

In the pre-experiment calibration phase, participants were tested only once in their ability to match five electrical‐stimulation levels to the 0-100 NPS scale, before any feedback manipulation started. During this pre-experiment calibration we required that each level was classified correctly on ≥ 75 % of the four repetitions; “correct” meant falling within ± 5 NPS units of the target anchor (e.g., a response of 25–35 was accepted for the 30/100 anchor). This procedure served one purpose only: to make sure that every participant entered the main experiment with three unambiguously distinguishable stimulation levels (30 / 50 / 70). We integrated this point in the revised manuscript lines 263-270.

Once the real task began, the context changed: shocks are unpredictable, attention is drawn to the heartbeat, and participants must judge both intensity and unpleasantness. In this full experimental setting the no-feedback block indeed shows considerable variability, even for the pain intensity ratings. Participants mean rating on the NPS scale was 46.4, with a standard deviation of 11.9 - thus participants vary quite strongly in their mean ratings (range 14.5 to 70). Moreover, while all participants show a positive correlation between actual intensities and their ratings (i.e., they rate the higher intensities as more intense than the lower ones), they vary in how much of the scale they use, with differences between reported highest and lowest intensities ranging between 8 and 91, for the participants showing the smallest and largest differences, respectively.

Thus, while we simplified the analysis to remove the difference scoring relative to the congruent trials and now use these congruent trials as an additional condition in the analysis, we retained the normalisation procedure to account for the in-fact-existing between-participant variability, and ensure consistency with prior research (Bartolo et al., 2013; Cecchini et al., 2020; Riello et al., 2019) and our a priori analysis plan.

However, to ensure we fully address your point here (and the other reviewers’ points about potential additional factors affecting the effects, like trial number and stimulus intensity), we also report an additional linear mixed-effects model analysis without normalization. It includes every feedback level as condition (No-Feedback, Congruent, Slower, Faster), plus additional predictors for actual stimulus intensity and trial rank within the experiment (as suggested by the other reviewers). This confirms that all relevant results remain intact once baseline and congruent trials are explicitly included in the model.

In brief, cross‐experiment analyses demonstrated that the Faster vs Slower contrast was markedly larger when the feedback was interoceptive than when it was exteroceptive. This held for heart-rate deceleration (b = 0.94 bpm, p = .005), for increases in unpleasantness (b = -0.16 Likert units, p = .015), and in pain-intensity ratings (b = -3.27 NPS points, p = .037).

These findings were then further confirmed by within-experiment analyses. Within the interoceptive experiment, the mixed-model on raw scores replicated every original effect: heart rate was lower after Faster than Slower feedback (estimate = –0.69 bpm, p = .005); unpleasantness was higher after Faster than Slower feedback (estimate = 0.19, p < .001); pain-intensity rose after Faster versus Slower (estimate=-4.285, p < .001). In the exteroceptive experiment, however, none of these Faster–Slower contrasts reached significance for heart rate (all ps > .33), unpleasantness (all ps > .43) or intensity (all ps > .10). Because these effects remain significant even with No-Feedback and Congruent trials explicitly included in the model and vanish under exteroceptive control, the supplementary, non-normalised analyses confirm that the faster vs. slower interoceptive feedback uniquely lowers anticipatory heart rate while amplifying both intensity and unpleasantness of pain, independent of data transformation or reference conditions. Please see Supplementary analyses for further details.

Bartolo, M., Serrao, M., Gamgebeli, Z., Alpaidze, M., Perrotta, A., Padua, L., Pierelli, F., Nappi, G., & Sandrini, G. (2013). Modulation of the human nociceptive flexion reflex by pleasant and unpleasant odors. PAIN, 154(10), 2054-2059.

Cecchini, M. P., Riello, M., Sandri, A., Zanini, A., Fiorio, M., & Tinazzi, M. (2020). Smell and taste dissociations in the modulation of tonic pain perception induced by a capsaicin cream application. European Journal of Pain, 24(10), 1946-1955.

Riello, M., Cecchini, M. P., Zanini, A., Di Chiappari, M., Tinazzi, M., & Fiorio, M. (2019). Perception of phasic pain is modulated by smell and taste. European Journal of Pain, 23(10), 1790-1800.

I could initially not find a rationale for bringing upfront the comparison between faster vs. slower HR acoustic feedback when in principle the intuitive comparisons would be faster vs. congruent and slower vs. congruent feedback. This is even more relevant considering that in the proposed main comparison, the congruent feedback does not play a role: since Δ outcomes are calculated as (faster - congruent) and (slower - congruent), a paired t-test between Δ faster and Δ slower outcomes equals (faster - congruent) - (slower - congruent) = (faster - slower). I later realized that the statistical comparison (paired t-test) of pain intensity ratings of faster vs. slower acoustic feedback is significant in experiment 1 but not in experiment 2, which in principle would support the argument that interoceptive, but not exteroceptive, feedback modulates pain perception. However, the "simple" t-tests show that faster feedback modulates pain perception in both experiments, although the effect is larger in experiment 1 (interoceptive feedback) compared to experiment 2 (exteroceptive feedback).

The comparison between faster and slower feedback is indeed crucial, and we regret not having made this clearer in the first version of the manuscript. As noted in our response to your point in the public review, this comparison is both statistically most powerful, and theoretically the most appropriate, as it controls for any influence of salience or surprise when heart rates deviate (in either direction) from what is expected. It therefore provides a clean measure of how much accelerated heartrate affects pain perception and physiological response, relative to an equal change in the opposite direction. However, as noted above, in the new version of the manuscript we have now removed the analysis via difference scores, and directly compared all three relevant conditions (faster, congruent, slower), first via an ANOVA and then with follow-up planned t-tests.

Please refer to our previous response for further details (i.e., Furthermore, the researchers propose the comparison of faster vs. slower delta HR acoustic feedback throughout the manuscript when the natural comparison is the incongruent vs. the congruent feedback [..]).

The design of experiment two involves the selection of knocking wood sounds to act as exteroceptive acoustic feedback. Since the purpose is to test whether sound affects pain intensity ratings, unpleasantness, and heart rate, it would have made sense to choose sounds that would be more likely to elicit such changes, e.g. Taffou et al. (2021), Chen & Wang (2022), Zhou et al. (2022), Tajadura-Jiménez et al. (2010). Whereas I acknowledge that there is a difference in effect sizes between experiment 1 and experiment 2 for the faster acoustic feedback, I am not fully convinced that this difference is due to the nature of the feedback (interoceptive vs. exteroceptive), since a similar difference could arguably be obtained by exteroceptive sound with looming or rough qualities. Since the experiment was already carried out and this hypothesis cannot be tested, I suggest that the researchers moderate the inferences made in the Discussion regarding these results.

Please refer to our previous response for a previous detailed answer to this point in the Public Review (i.e., This could be influenced by the fact that the faster HR exteroceptive cue in experiment 2 also shows a significant modulatory effect [..]). As we describe there, we see little grounds to suspect such a non-specific influence of acoustic parameters, as it is specifically the sensitivity to the change in heart rate (faster vs slower) that is affected by our between-experiment manipulation, not the overall response to the different exteroceptive or interoceptive sounds. Moreover, the specific change induced by the faster interoceptive feedback - a heartrate deceleration - is not consistent with a change in arousal or alertness (which would have predicted an increase in heartrate with increasing arousal). See also Discussion-Accounting for general unspecific contributions.

Additionally, the fact that no significant effects were found for unpleasantness ratings or heart rate (absence of evidence) should not be taken as proof that faster exteroceptive feedback does not induce an effect on these outcomes (evidence of absence). In this case, it could be that there is actually no effect on these variables, or that the experiment was not sufficiently powered to detect those effects. This would depend on the SESOIs for these variables, which as stated before, was not properly justified.

We very much agree that the absence of significant effects should not be interpreted as definitive evidence of absence. Indeed, we were careful not to overinterpret the null findings for heart rate and unpleasantness ratings, and we conducted additional analyses to clarify their interpretation. First, the TOST analysis shows that any effects in Experiment 2 are (significantly) smaller than the smallest effect size that can possibly be detected in our experiment, given the experimental parameters (number of participants, type of test, alpha level). Second, and more importantly, we run between-experiments comparisons (see Results Experiment 2, and Supplementary materials, Cross-experiment analysis between-subjects model) of the crucial difference in the changes induced by faster and slower feedback. This showed that the differences were larger with interoceptive (Experiment 1) than exteroceptive cues (Experiment 2). Thus, even if a smaller than is in principle detectable effect is induced by the exteroceptive cues in Experiment 2, it is smaller than with interoceptive cues in Experiment 1.

To ensure we fully address this point, we have now simplified our main analysis (main manuscript), replicated it with a different analysis (Supplementary material), we motivate more clearly (Methods Experiment 1), why the comparison between faster and slower feedback is crucial, and we make clearer that the difference between these conditions is larger in Experiment 1 than Experiment 2 (Results Experiment 2). Moreover, we went through the manuscript and ensured that our wording does not over-interpret the absence of effects in Experiment 2, as an absence of a difference.

The section "Additional comparison analysis between experiments" encompasses in a way all possible comparisons between levels of the different factors in both experiments. My original suggestion regarding the use of a mixed linear model with covariates is still valid for this case. This analysis also brings into question another aspect of the experimental design: what is the rationale for dividing the study into two experiments, considering that variability and confounding factors would have been much better controlled in a single experimental session that includes all conditions?

We thank the reviewer for their comment. We would like to note, first, that the between-experiment analyses did not encompass all possible comparisons between levels, as it just included faster and slower feedback for the within-experiment comparison Instead, they focus on the specific interaction between faster and slower feedback on the one hand, and interoceptive vs exteroceptive cues on the other. This interaction essentially compares, for each dependent measure (HR, pain unpleasantness, pain intensity), the difference between faster and slower feedback in Experiment 1 with that the same difference in Experiment 2 (and would produce identical p values to a between-experiment t-test). The significant interactions therefore indicate larger effects of interoceptive cues than exteroceptive ones for each of the measures. To make this clearer, we have now exchanged the analysis with between-experiment t-tests of the difference between faster and slower feedback for each measure (Results Experiment 2), producing identical results. Moreover, as suggested, we also now report linear mixed model analyses (see Supplementary Materials), which provide a comprehensive comparison across experiments.

Regarding the experimental design, we appreciate the reviewer’s suggestion regarding a within-subject crossover design. While such an approach indeed offers greater statistical power by reducing interindividual variability (Charness, Gneezy, & Kuhn, 2012), we intentionally chose a between-subjects design due to theoretical and methodological considerations specific to deceptive feedback paradigms. First, carryover effects are a major concern in deception studies. Participants exposed to one type of feedback could develop suspicion or adaptive strategies that would alter their responses in subsequent conditions (Martin & Sayette, 1993). Expectancy effects could thus contaminate results in a crossover design, particularly when feedback manipulation becomes apparent. In line with this idea, past studies on false cardiac feedback (e.g., Valins, 1966; Pennebaker & Lightner, 1980) often employed between-subjects or blocked designs to maintain the ecological validity of the illusion.

Charness, G., Gneezy, U., & Kuhn, M. A. (2012). Experimental methods: Between-subject and within-subject design. Journal of economic behavior & organization, 81(1), 1-8.

Martin, C. S., & Sayette, M. A. (1993). Experimental design in alcohol administration research: limitations and alternatives in the manipulation of dosage-set. Journal of studies on alcohol, 54(6), 750-761.

Pennebaker, J. W., & Lightner, J. M. (1980). Competition of internal and external information in an exercise setting. Journal of personality and social psychology, 39(1), 165.

Valins, S. (1966). Cognitive effects of false heart-rate feedback. Journal of personality and social psychology, 4(4), 400.

References

Chen ZS, Wang J. Pain, from perception to action: A computational perspective. iScience. 2022 Dec 1;26(1):105707. doi: 10.1016/j.isci.2022.105707.

Dienes Z. Obtaining Evidence for No Effect. Collabra: Psychology 2021 Jan 4; 7 (1): 28202. doi: 10.1525/collabra.28202

King MT. A point of minimal important difference (MID): a critique of terminology and methods. Expert Rev Pharmacoecon Outcomes Res. 2011 Apr;11(2):171-84. doi: 10.1586/erp.11.9.

Lakens D. Sample Size Justification. Collabra: Psychology 2022 Jan 5; 8 (1): 33267. doi: 10.1525/collabra.33267

Nosek BA, Ebersole CR, DeHaven AC, Mellor DT. The preregistration revolution. Proc Natl Acad Sci U S A. 2018 Mar 13;115(11):2600-2606. doi: 10.1073/pnas.1708274114.

Sasaki K, Yamada Y. SPARKing: Sample-size planning after the results are known. Front Hum Neurosci. 2023 Feb 22;17:912338. doi: 10.3389/fnhum.2023.912338.

Taffou M, Suied C, Viaud-Delmon I. Auditory roughness elicits defense reactions. Sci Rep. 2021 Jan 13;11(1):956. doi: 10.1038/s41598-020-79767-0.

Tajadura-Jiménez A, Väljamäe A, Asutay E, Västfjäll D. Embodied auditory perception: The emotional impact of approaching and receding sound sources. Emotion. 2010, 10(2), 216-229.https://doi.org/10.1037/a0018422

Zhou W, Ye C, Wang H, Mao Y, Zhang W, Liu A, Yang CL, Li T, Hayashi L, Zhao W, Chen L, Liu Y, Tao W, Zhang Z. Sound induces analgesia through corticothalamic circuits. Science. 2022 Jul 8;377(6602):198-204. doi: 10.1126/science.abn4663.

**Reviewer #3 (Recommendations For The Authors):**

The manuscript would benefit from some spelling- and grammar checking.

Done

Discussion:

The discussion section is rather lengthy and would benefit from some re-structuring, editing, and sub-section headers.

In response, we have restructured and edited the Discussion section to improve clarity and flow.

I personally had a difficult time understanding how the data relates to the rubber hand illusion (l.623-630). I would recommend revising or deleting this section.

We thank the reviewer for this valuable feedback. We have revised the paragraph and made the parallel clearer (lines 731-739).

Other areas are a bit short and might benefit from some elaboration, such as clinical implications. Since they were mentioned in the abstract, I had expected a bit more thorough discussion here (l. 718).

Thank you for this suggestion. We have expanded the discussion to more thoroughly address the clinical implications of our interoceptive pain illusion (See Limitations and Future Directions paragraph).

Further, clarification is needed for the following:

I would like some more details on participant instructions; in particular, the potential difference in instruction between Exp. 1 and 2, if any. In Exp. 1, it says: (l. 280) "Crucially, they were also informed that over the 60 seconds preceding the administration of the shock, they were exposed to acoustic feedback, which was equivalent to their ongoing heart rate". Was there a similar instruction for Exp. 2? If yes, it would suggest a more specific effect of cardiac auditory feedback; if no, the ramifications of this difference in instructions should be more thoroughly discussed.

Thank you for this suggestion. We have clarified this point in the Procedure of Experiment 2 (548-550).
